# Supplementary figures and images for: Hsp70 is phosphorylated in a conserved response to DNA damage and contributes to cell cycle control
Source: eLife. 2026 May 27;15:RP110044. doi: 10.7554/eLife.110044 (PMC13215673; doi:10.7554/eLife.110044)

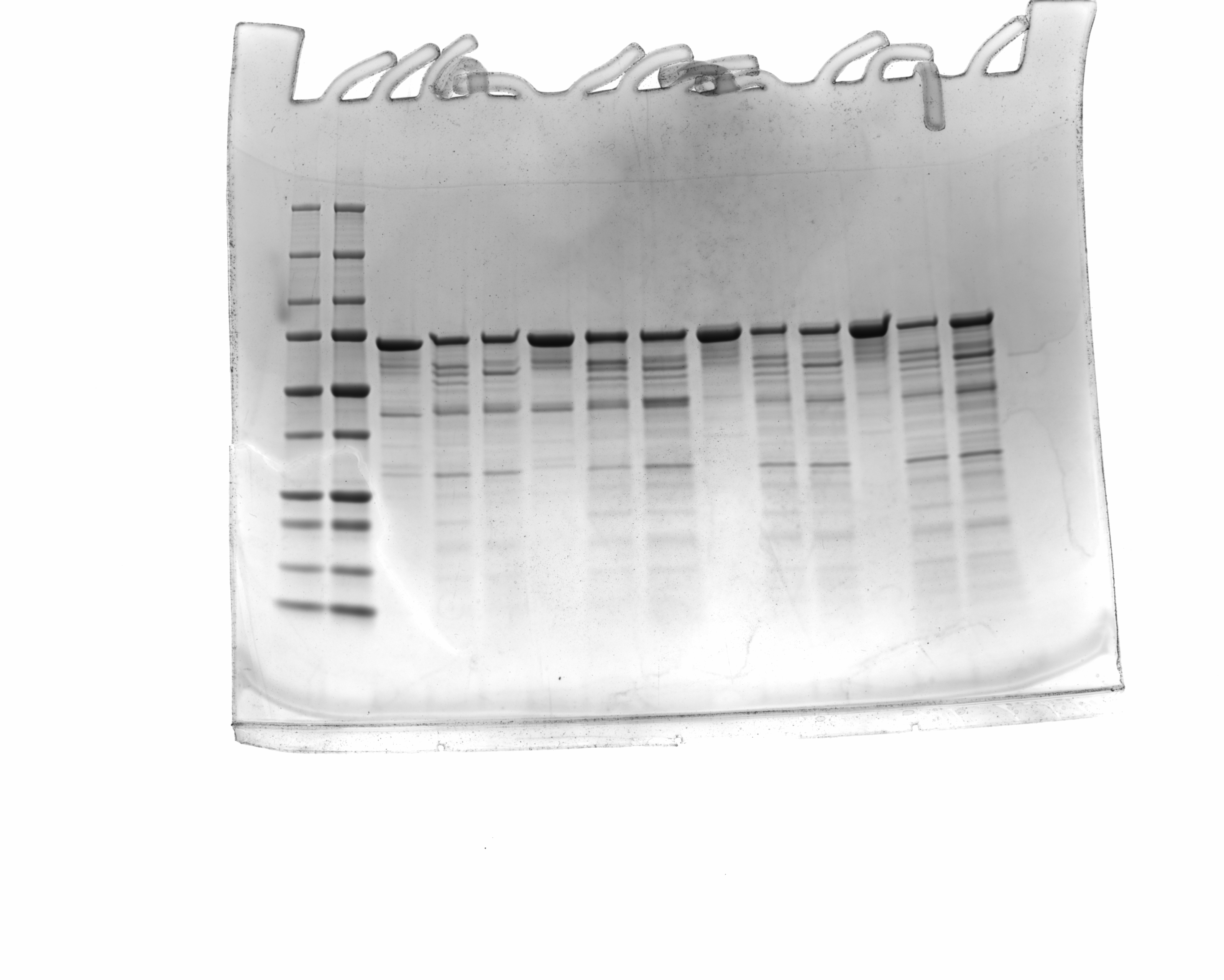

Supplement: Figure 1—source data 1. [file elife-110044-fig1-data1.zip › Figure 1-source data 1/Figure 1-source data 1.tif]

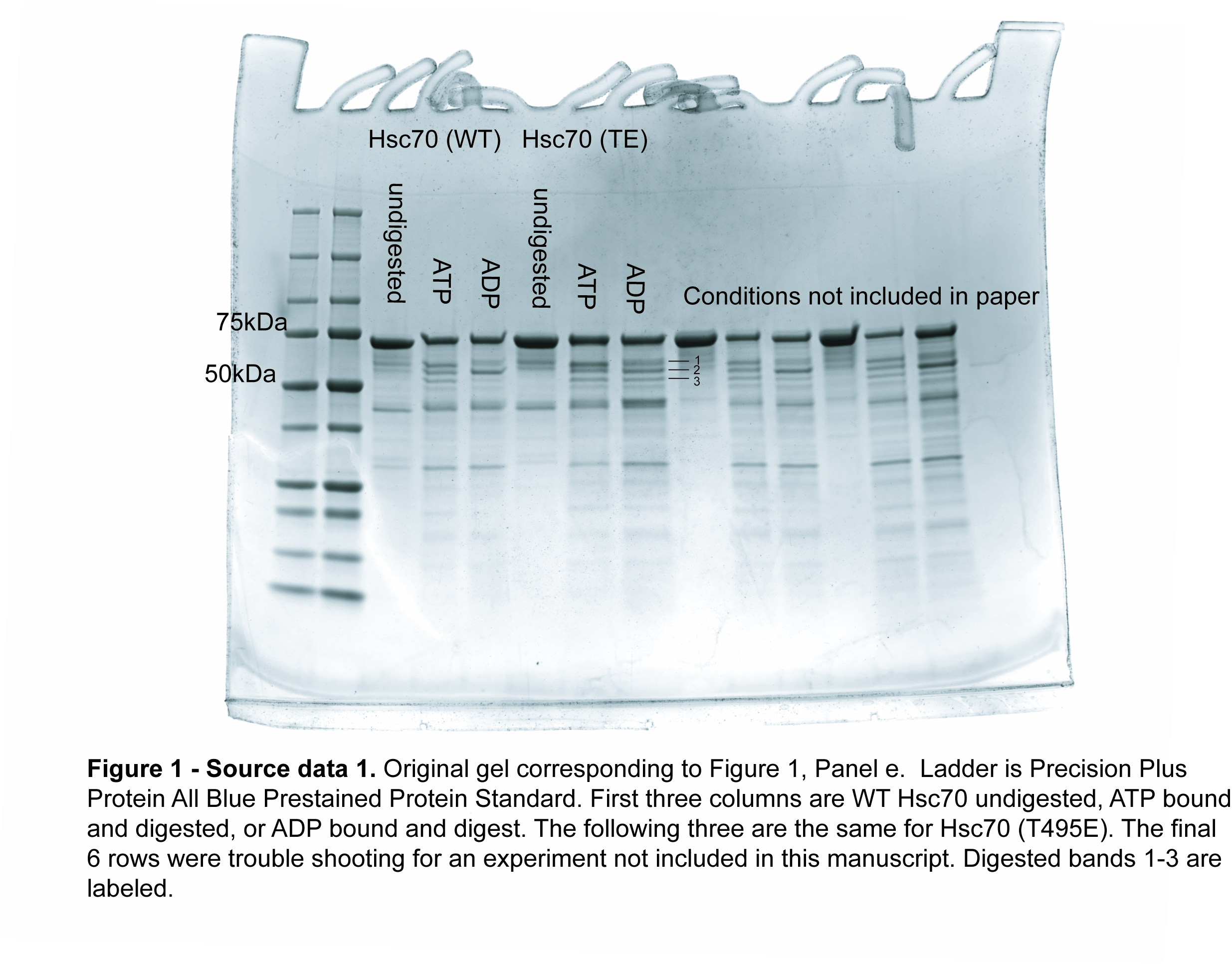

Supplement: Figure 1—source data 2. [file elife-110044-fig1-data2.zip › Figure 1-source data 2/Figure 1-source data 2.tif]

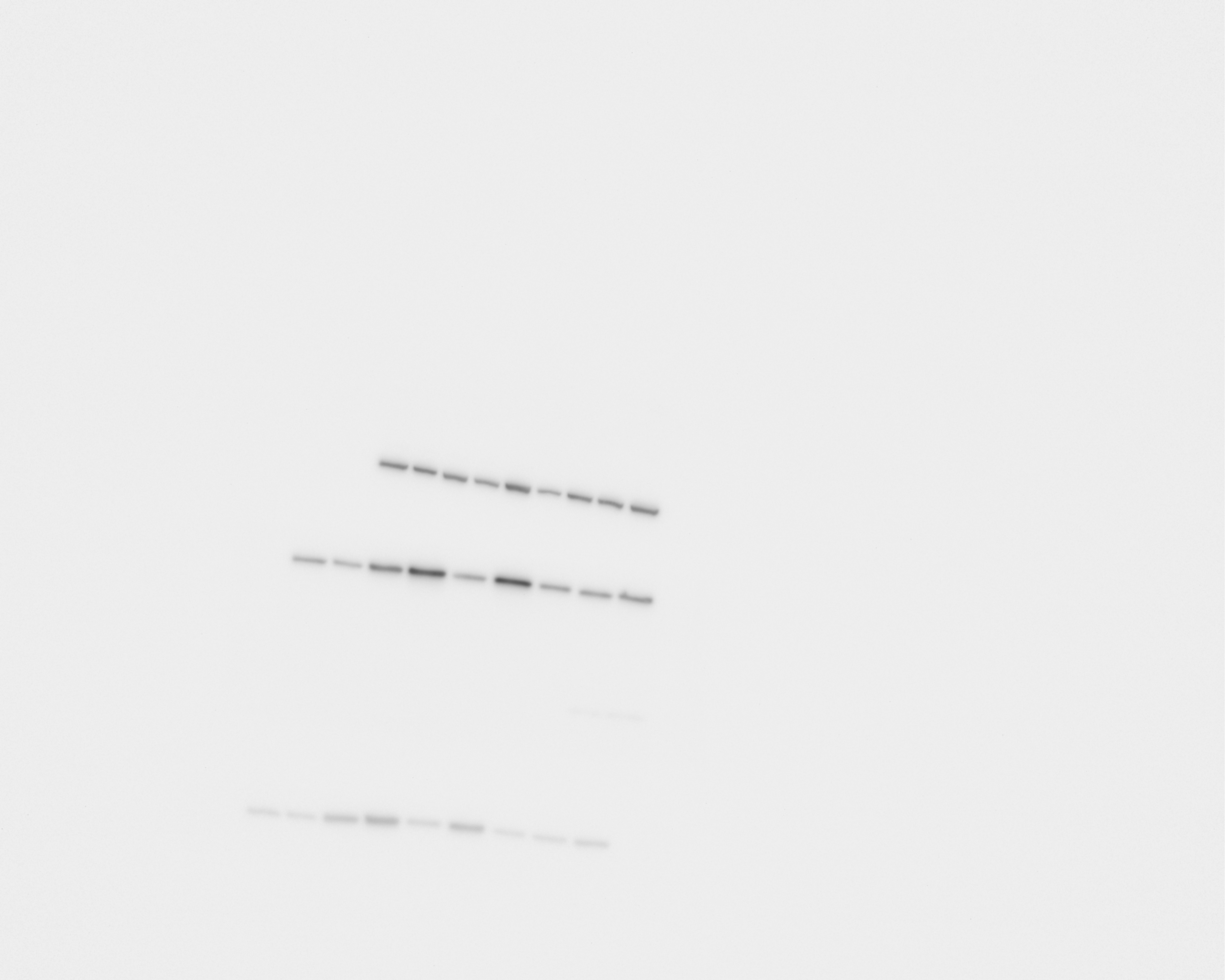

Supplement: Figure 2—source data 1. [file elife-110044-fig2-data1.zip › Figure 2-source data 1/Figure 2f-5.tif]

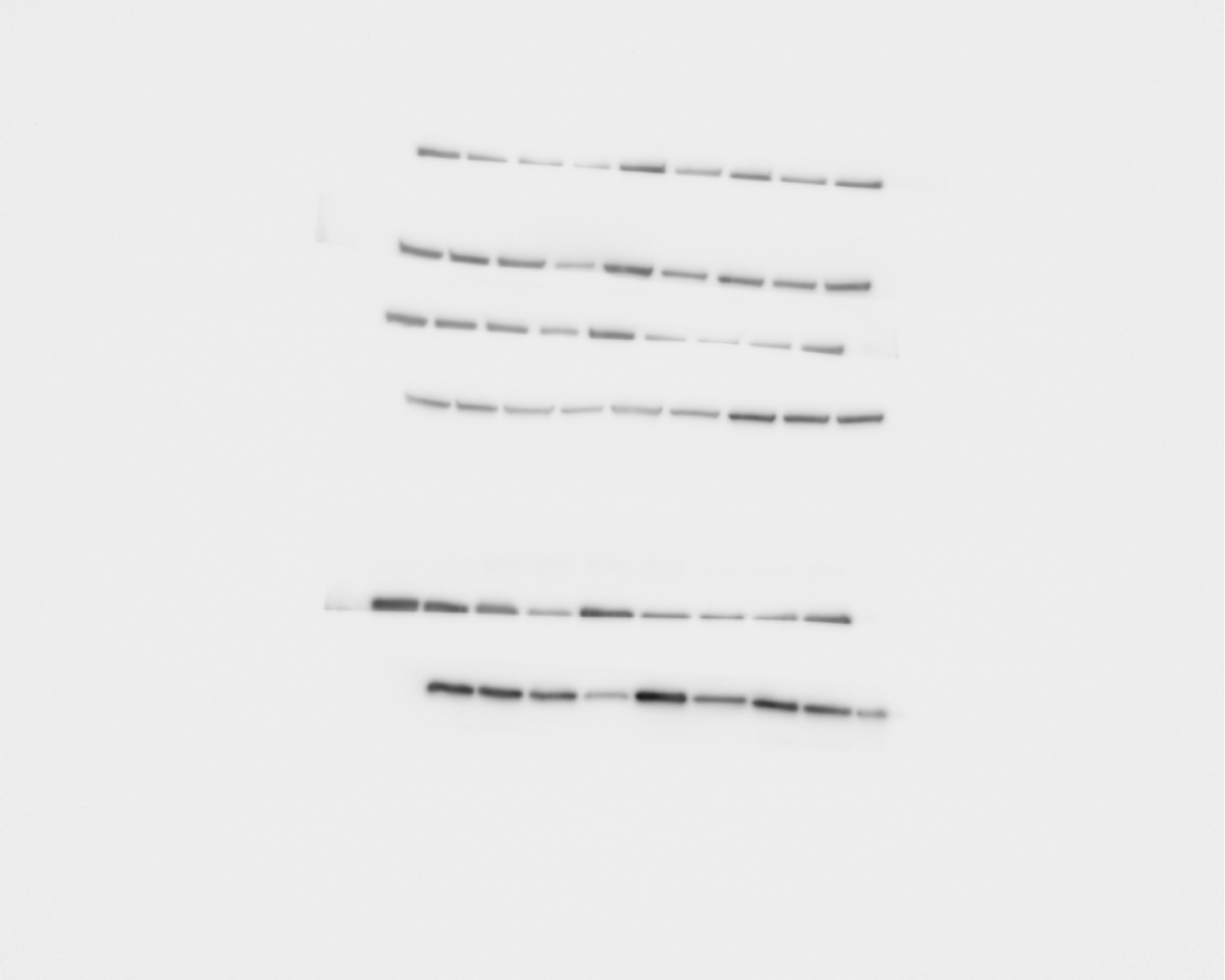

Supplement: Figure 2—source data 1. [file elife-110044-fig2-data1.zip › Figure 2-source data 1/Figure 2f-4.tif]

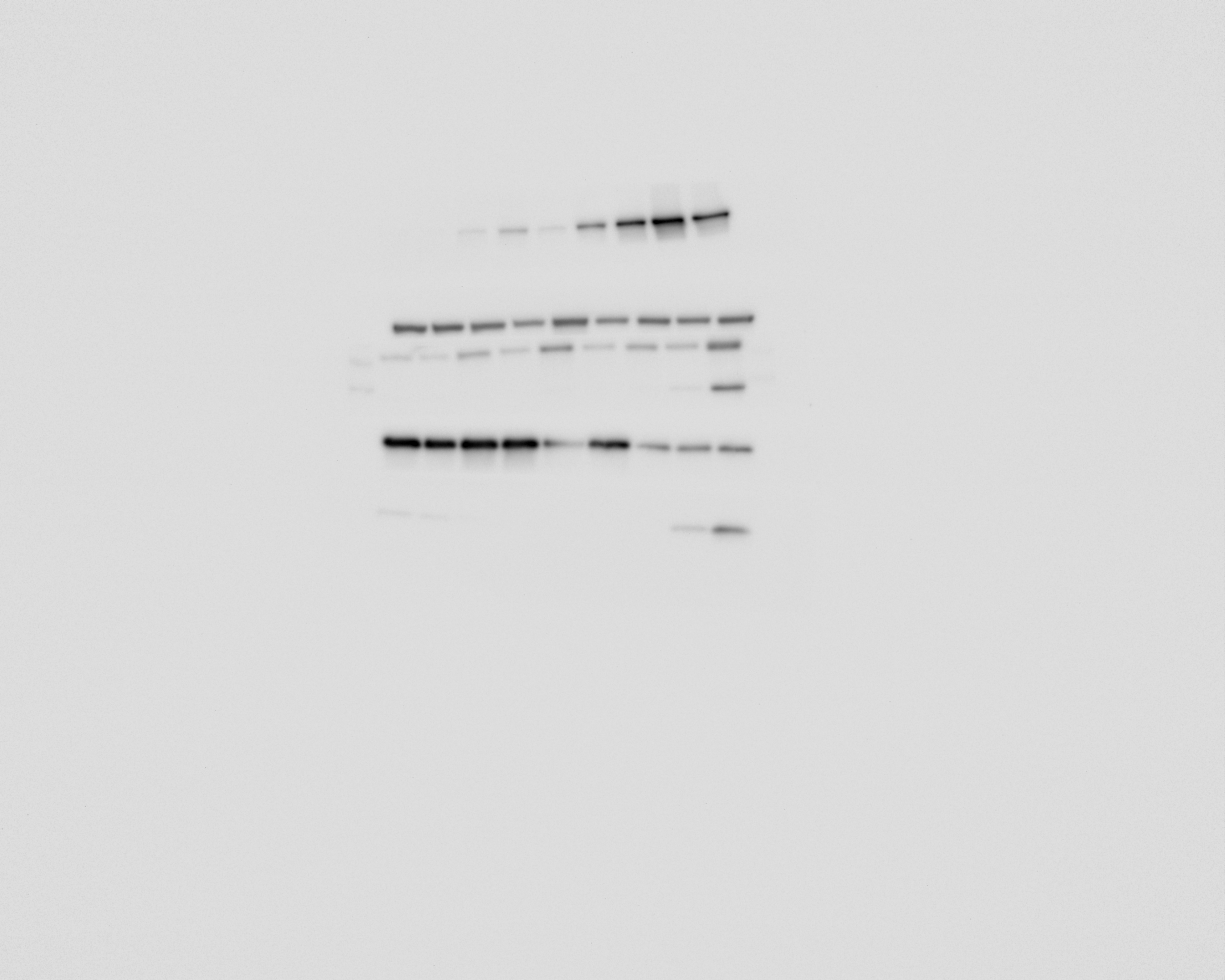

Supplement: Figure 2—source data 1. [file elife-110044-fig2-data1.zip › Figure 2-source data 1/Figure 2f-3.tif]

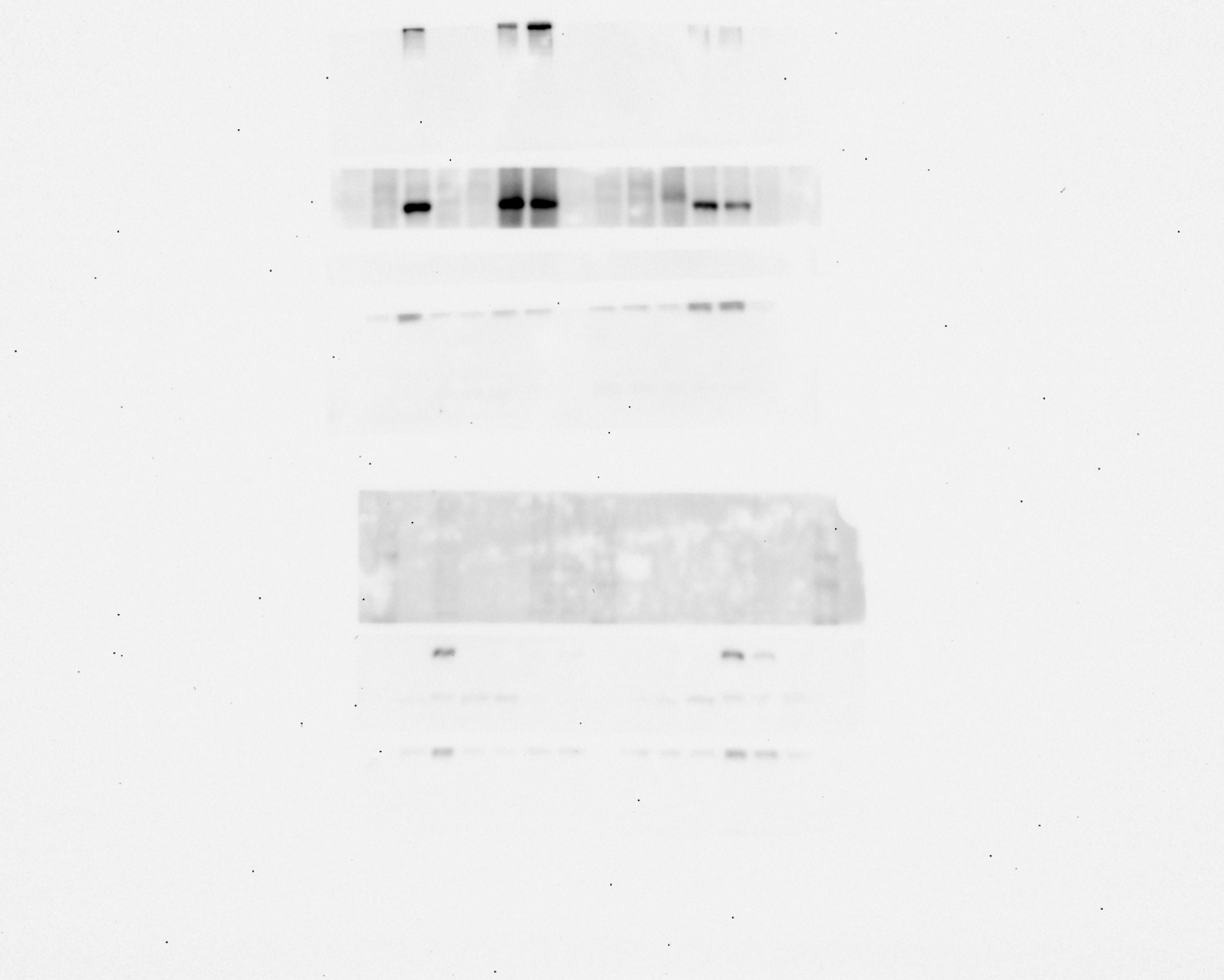

Supplement: Figure 2—source data 1. [file elife-110044-fig2-data1.zip › Figure 2-source data 1/Figure 2d-1.tif]

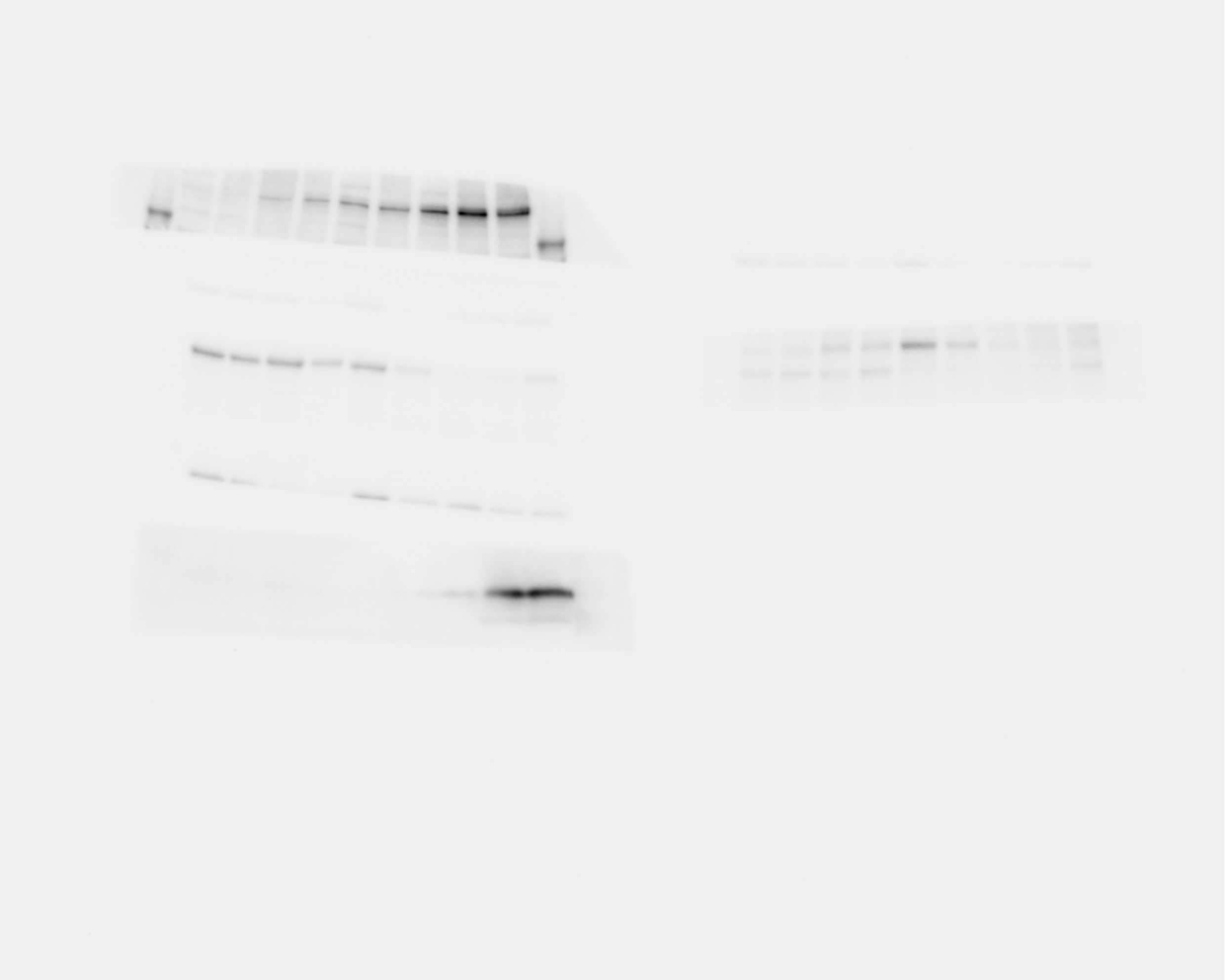

Supplement: Figure 2—source data 1. [file elife-110044-fig2-data1.zip › Figure 2-source data 1/Figure 2f-2.tif]

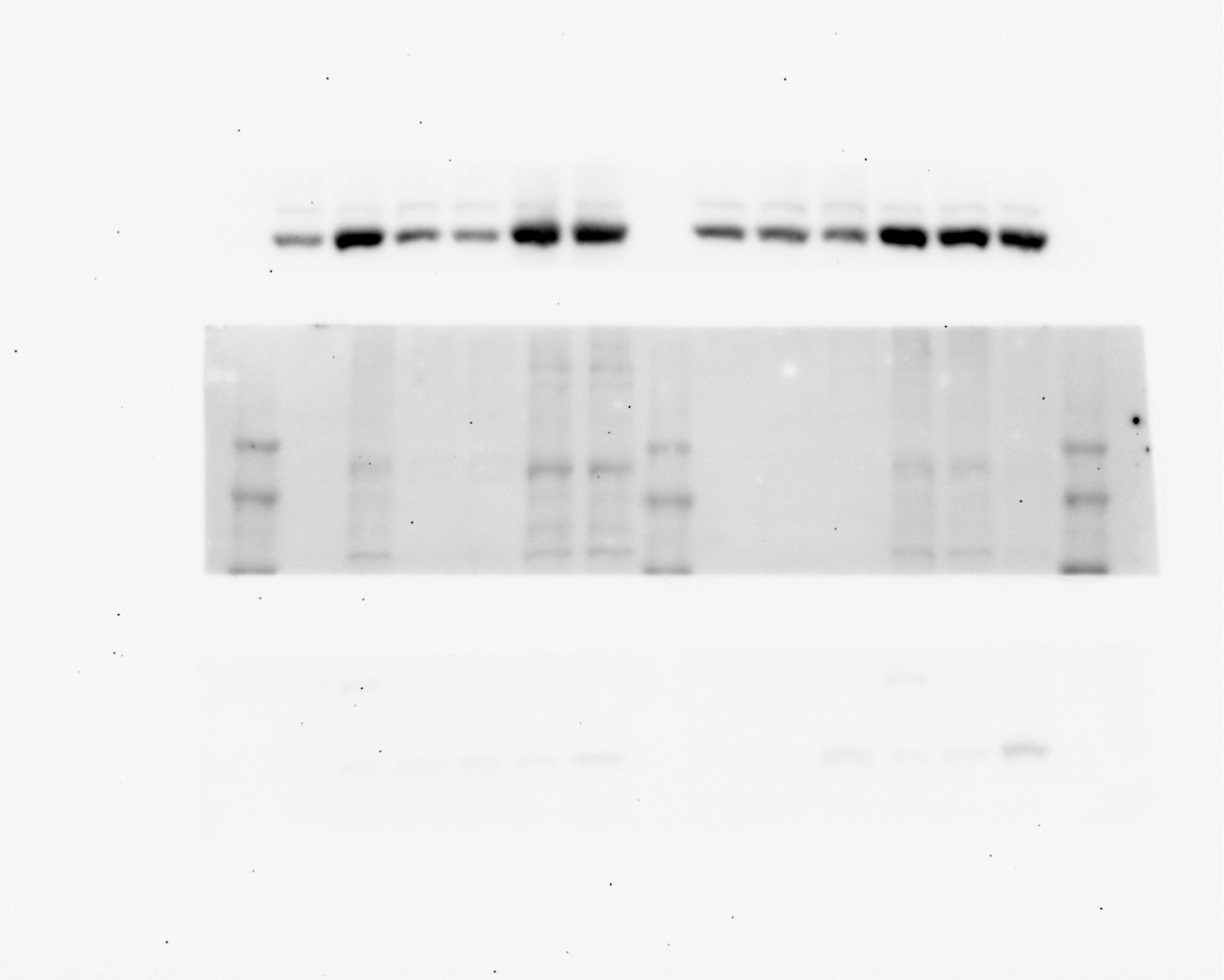

Supplement: Figure 2—source data 1. [file elife-110044-fig2-data1.zip › Figure 2-source data 1/Figure 2d-2.tif]

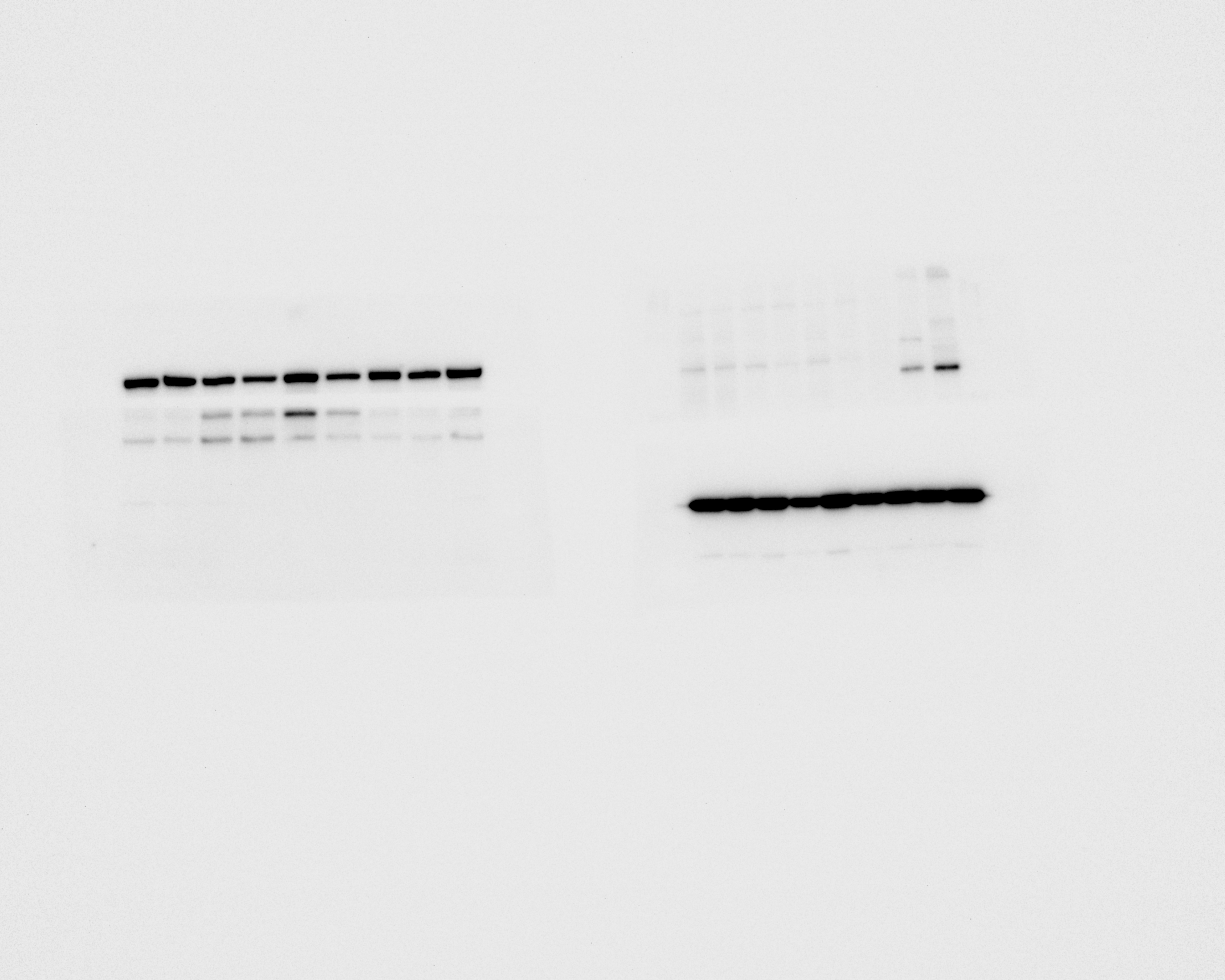

Supplement: Figure 2—source data 1. [file elife-110044-fig2-data1.zip › Figure 2-source data 1/Figure 2f-1.tif]

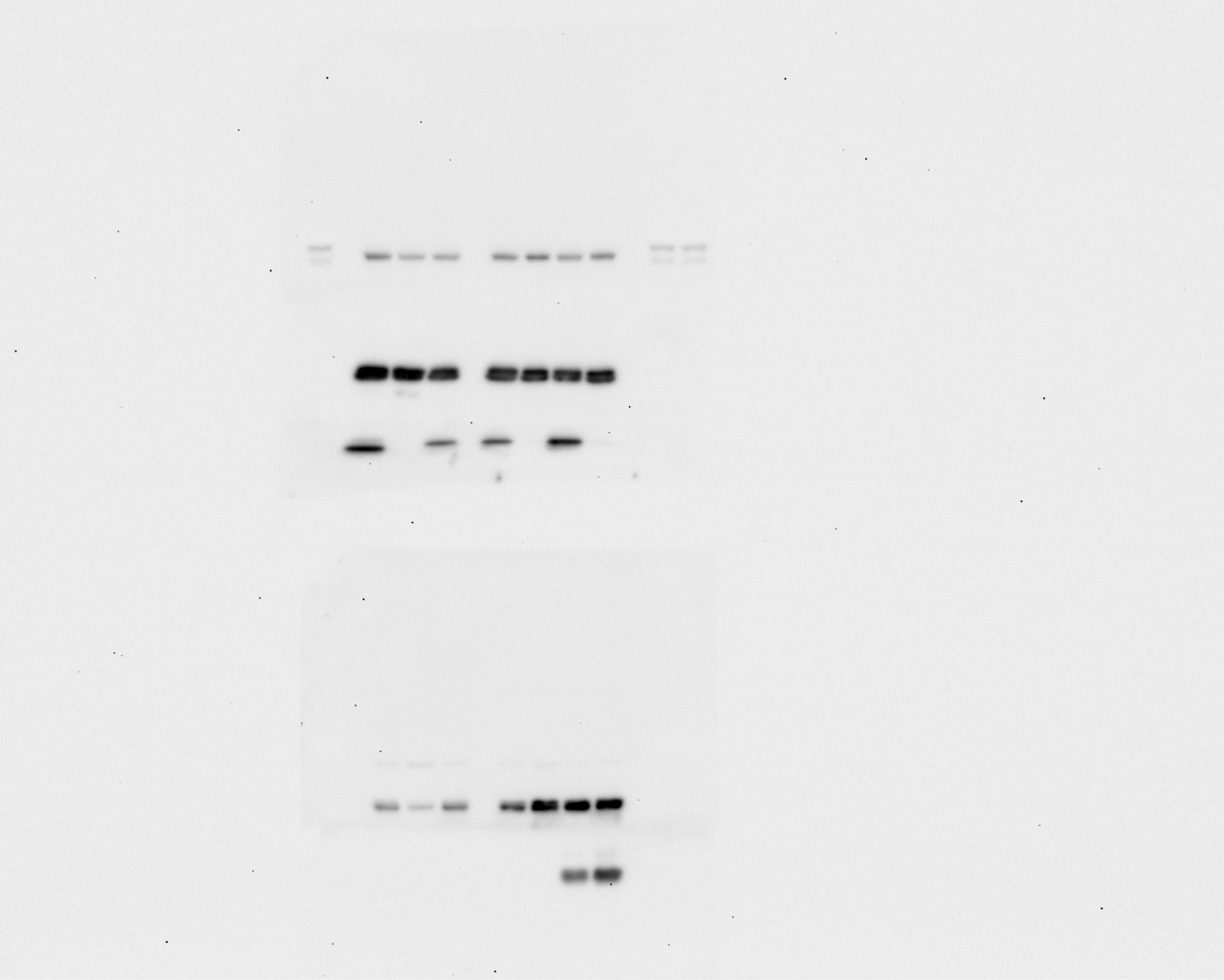

Supplement: Figure 2—source data 1. [file elife-110044-fig2-data1.zip › Figure 2-source data 1/Figure 2c-2.tif]

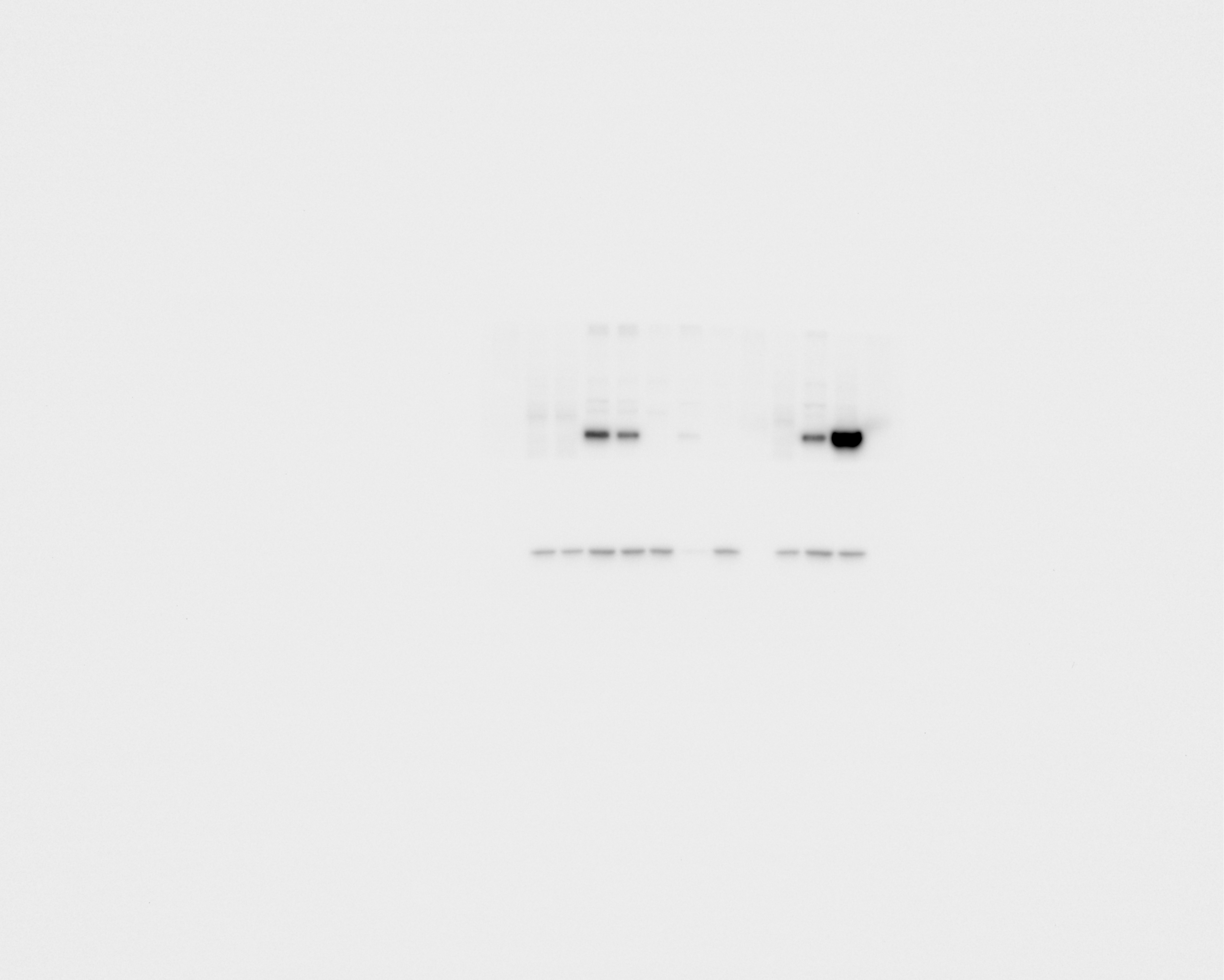

Supplement: Figure 2—source data 1. [file elife-110044-fig2-data1.zip › Figure 2-source data 1/Figure 2a-1.tif]

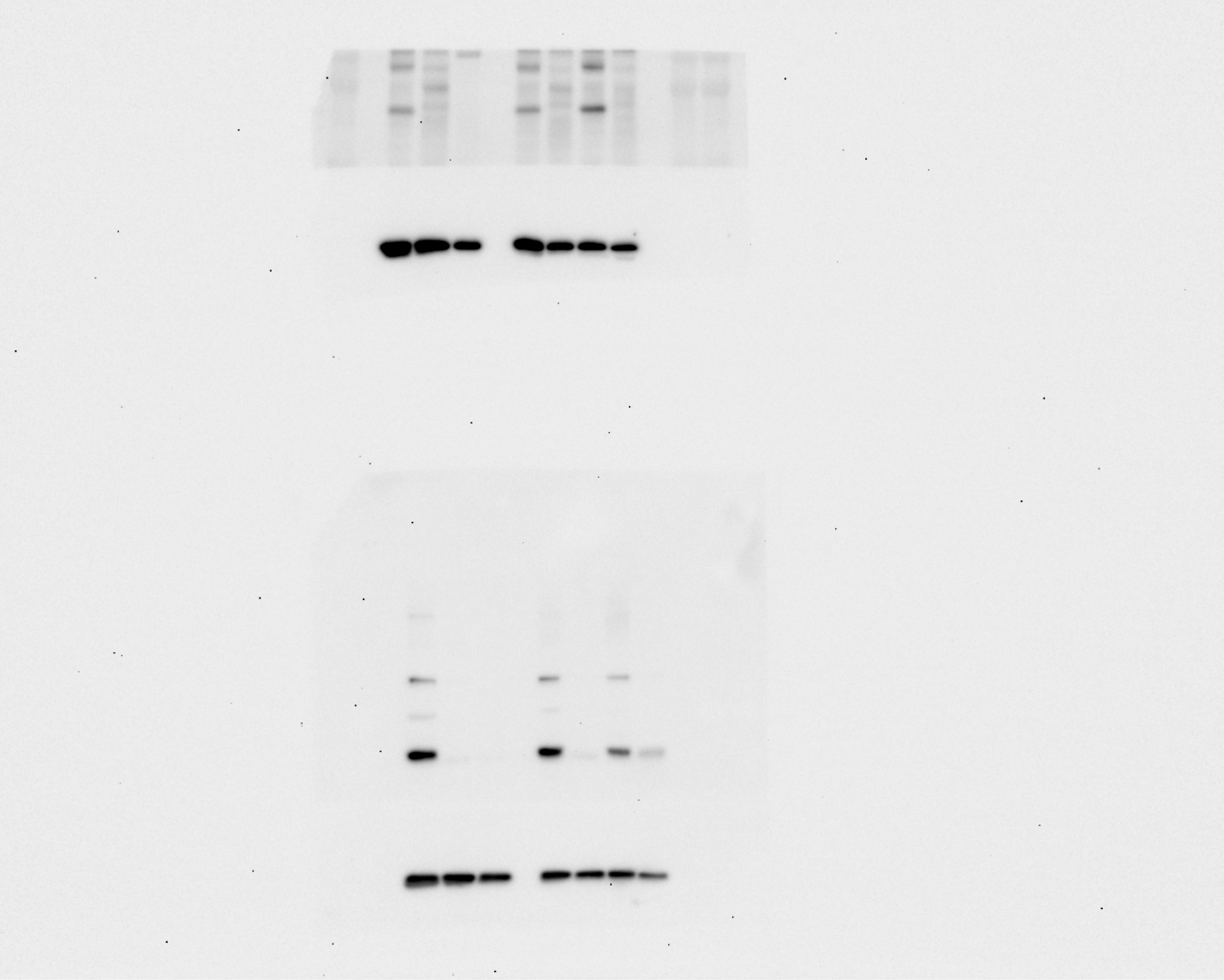

Supplement: Figure 2—source data 1. [file elife-110044-fig2-data1.zip › Figure 2-source data 1/Figure 2c-1.tif]

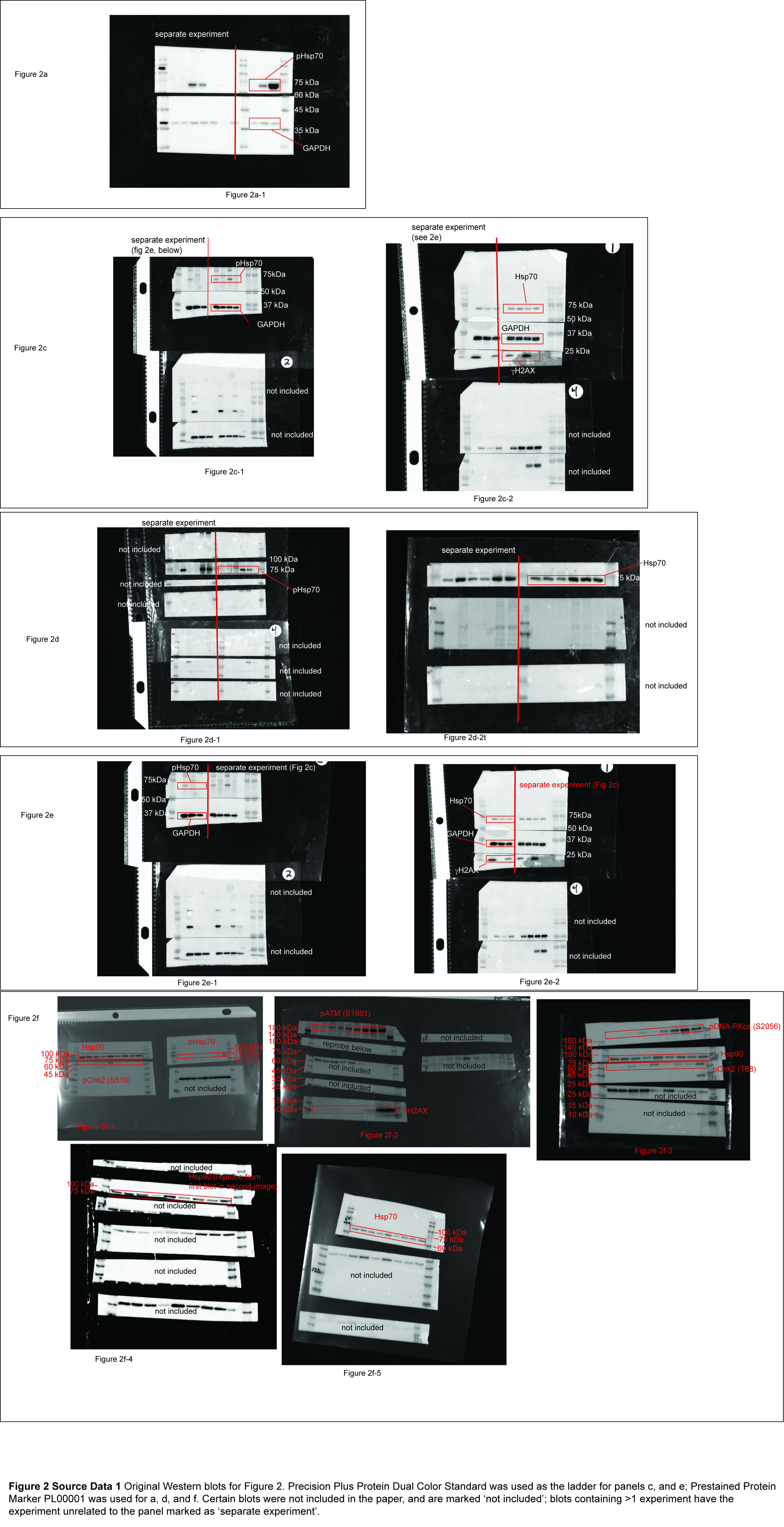

Supplement: Figure 2—source data 2. [file elife-110044-fig2-data2.zip › Figure 2-source data 2/Figure 2 - Source data 2.tif]

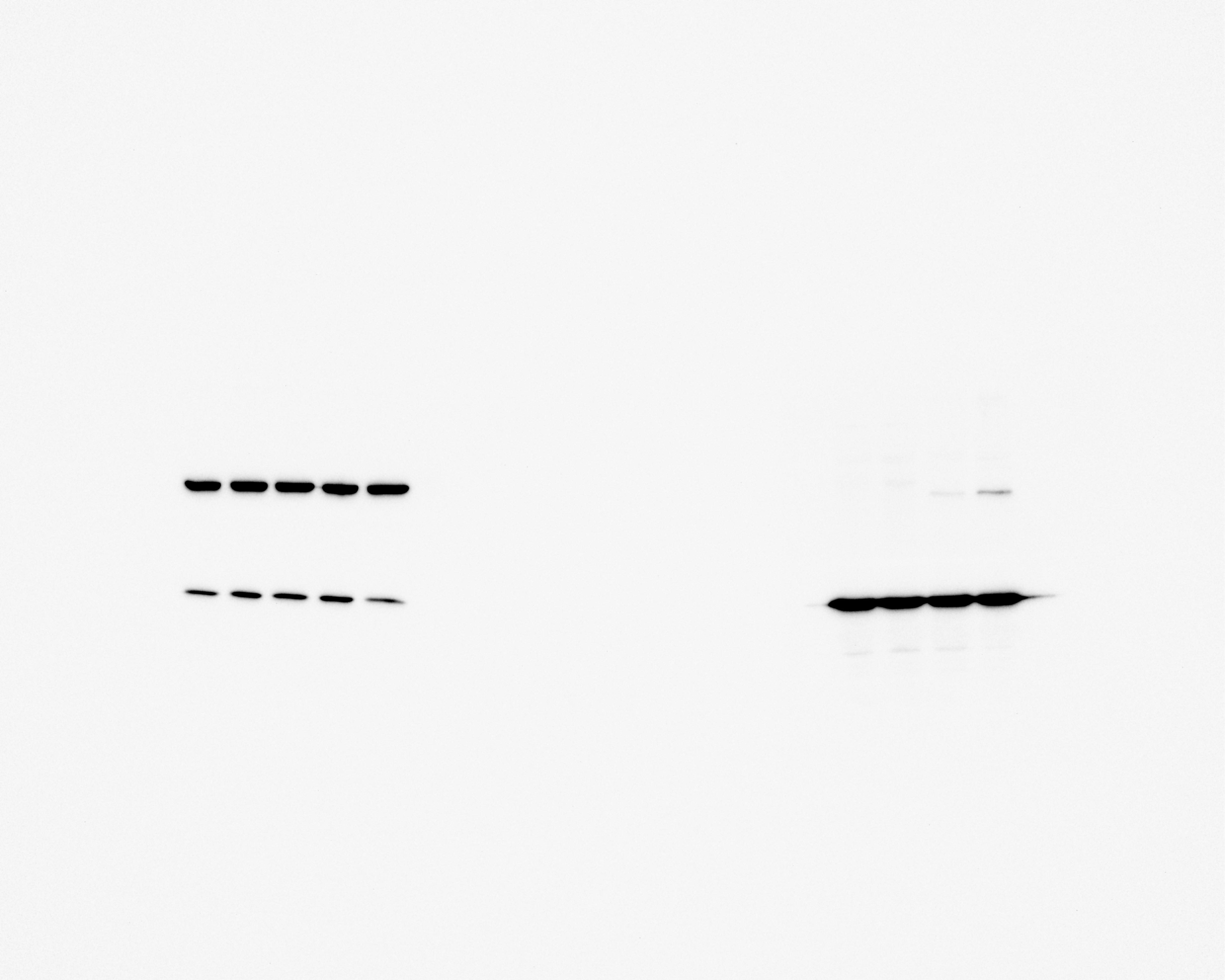

Supplement: Figure 2—figure supplement 1—source data 1. [file elife-110044-fig2-figsupp1-data1.zip › Figure 2 Supplement 1-source data 1/Figure 2 Supplement 1-a1.tif]

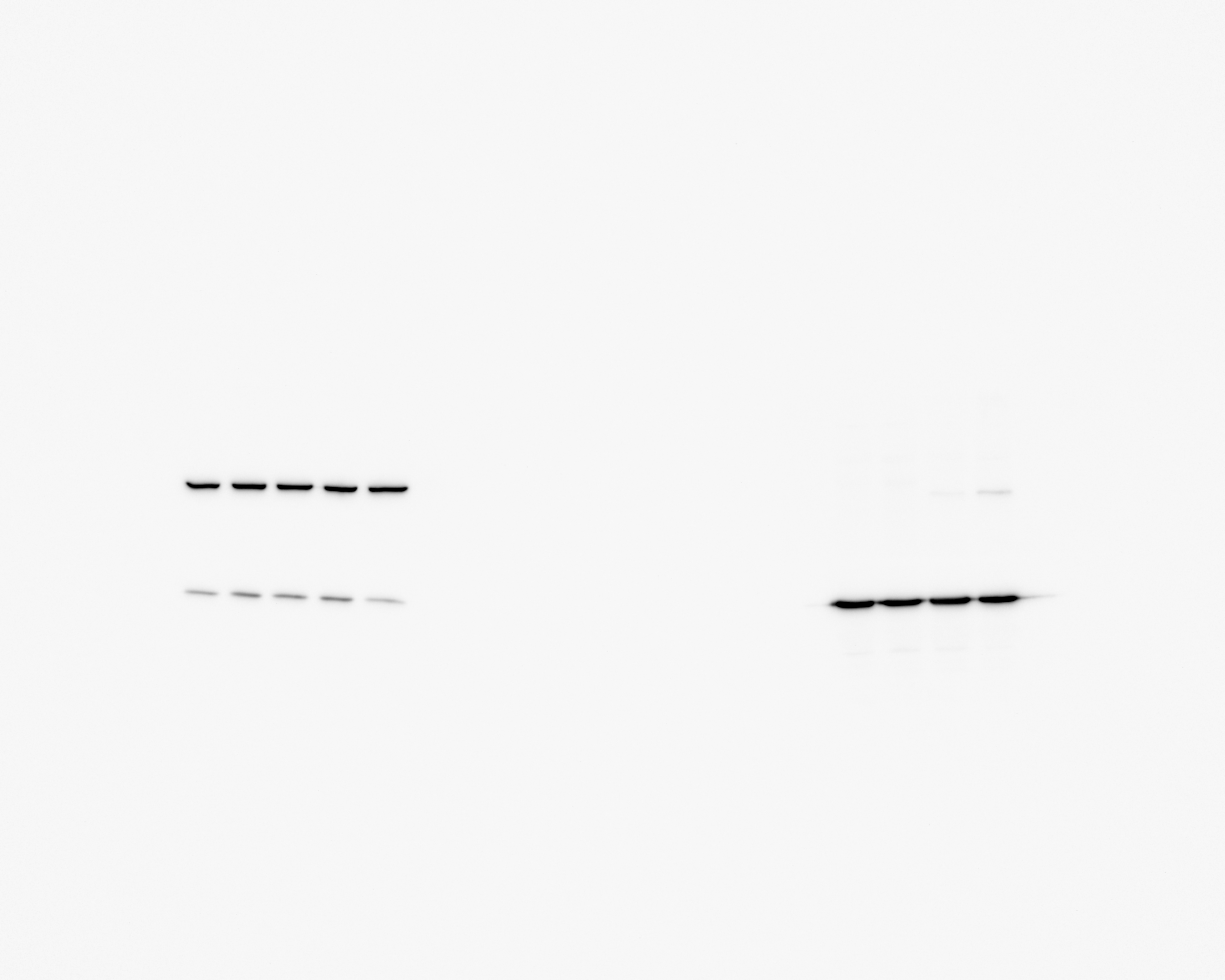

Supplement: Figure 2—figure supplement 1—source data 1. [file elife-110044-fig2-figsupp1-data1.zip › Figure 2 Supplement 1-source data 1/Figure 2 Supplement 1-a2.tif]

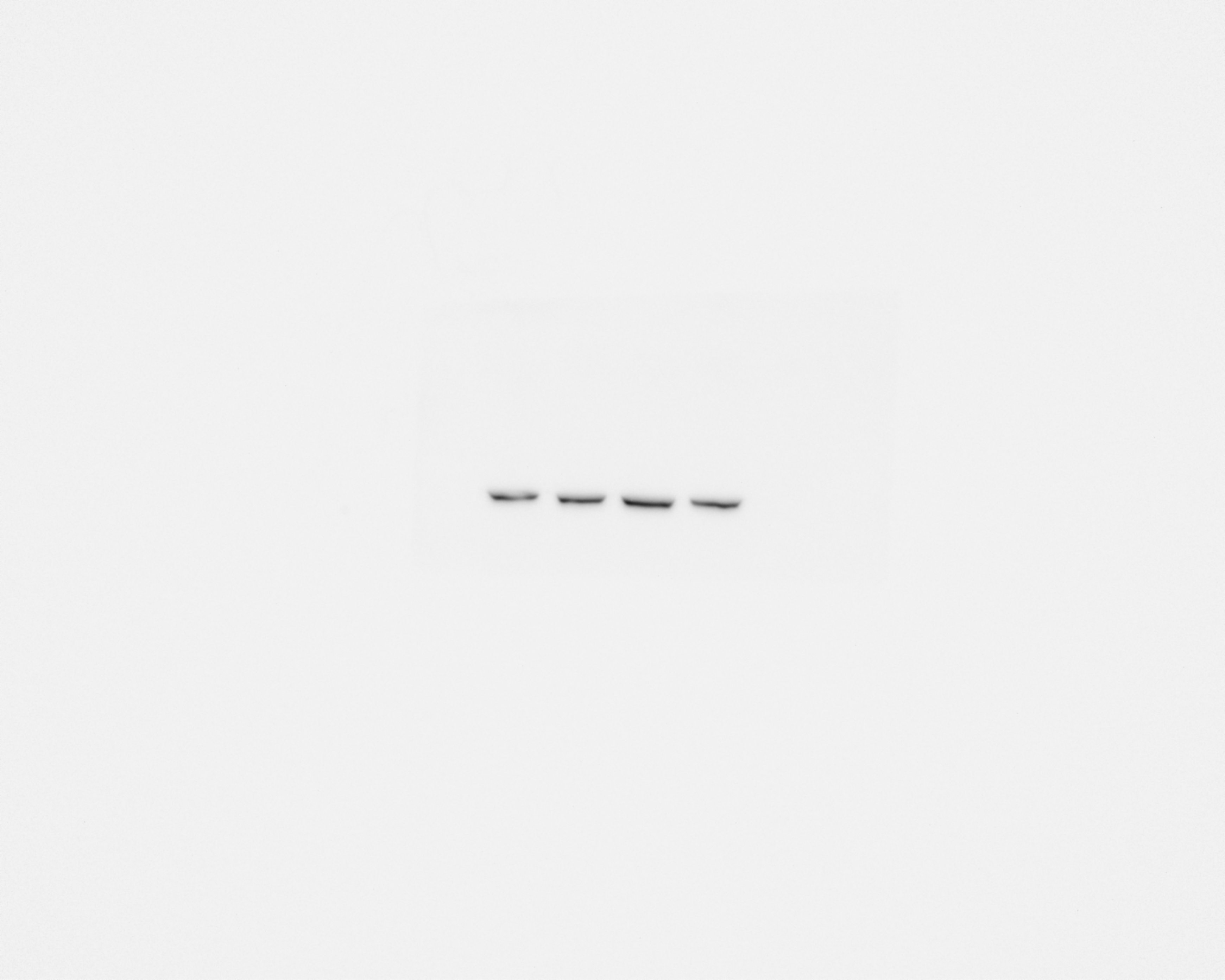

Supplement: Figure 2—figure supplement 1—source data 1. [file elife-110044-fig2-figsupp1-data1.zip › Figure 2 Supplement 1-source data 1/Figure 2 Supplement 1-a3.tif]

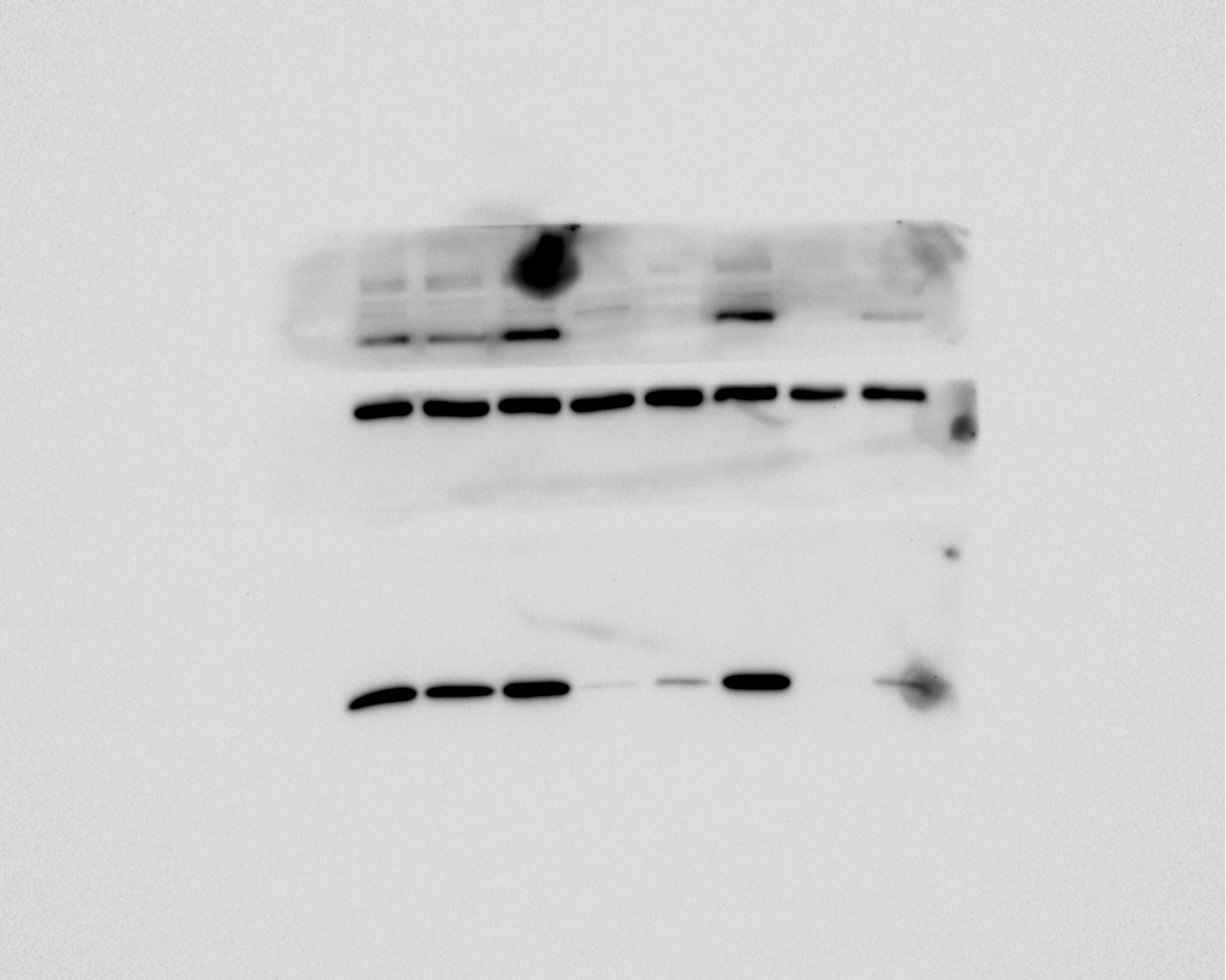

Supplement: Figure 2—figure supplement 1—source data 1. [file elife-110044-fig2-figsupp1-data1.zip › Figure 2 Supplement 1-source data 1/Figure 2 Supplement 1-de-1.tif]

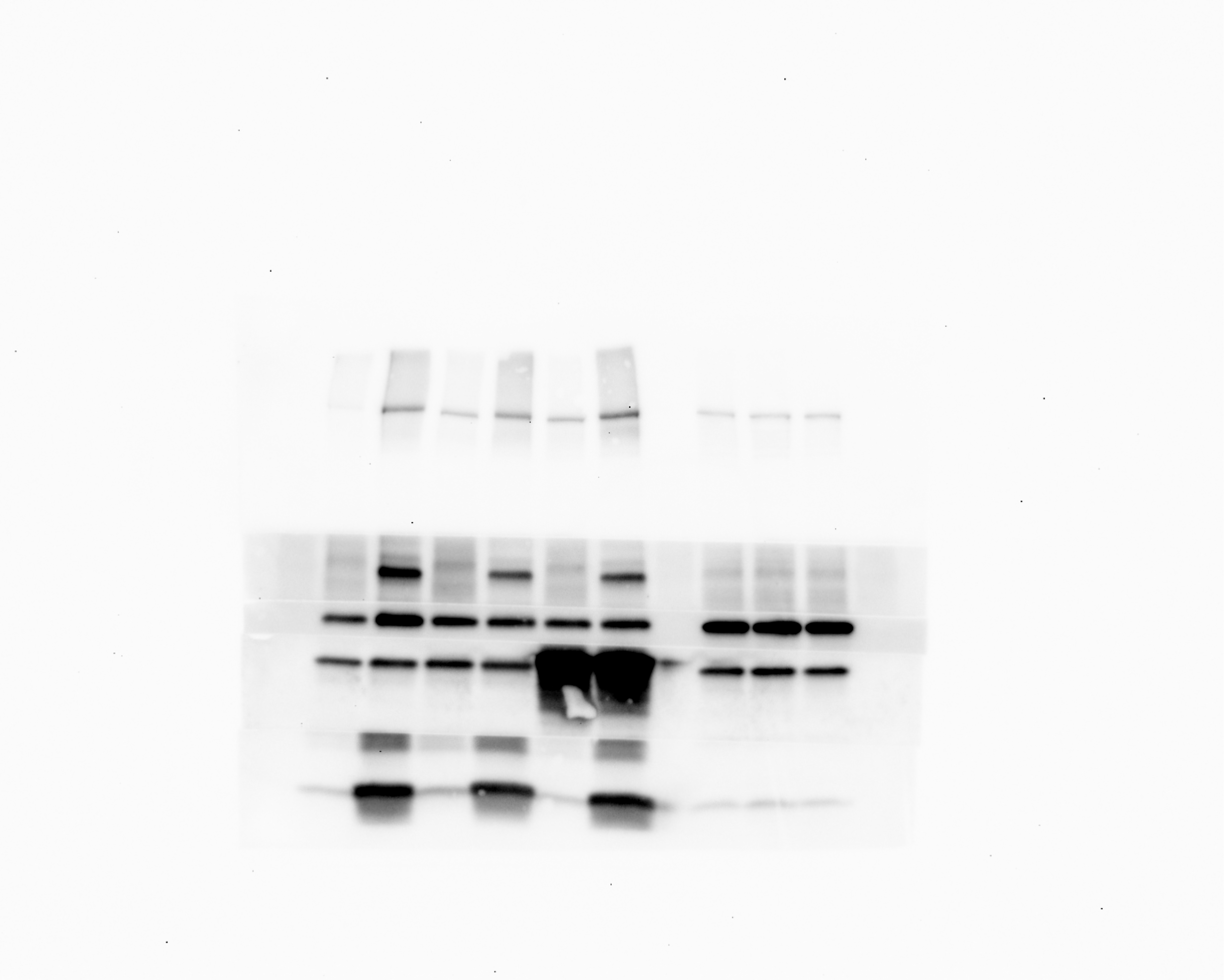

Supplement: Figure 2—figure supplement 1—source data 1. [file elife-110044-fig2-figsupp1-data1.zip › Figure 2 Supplement 1-source data 1/Figure 2 Supplement 1-c1.tif]

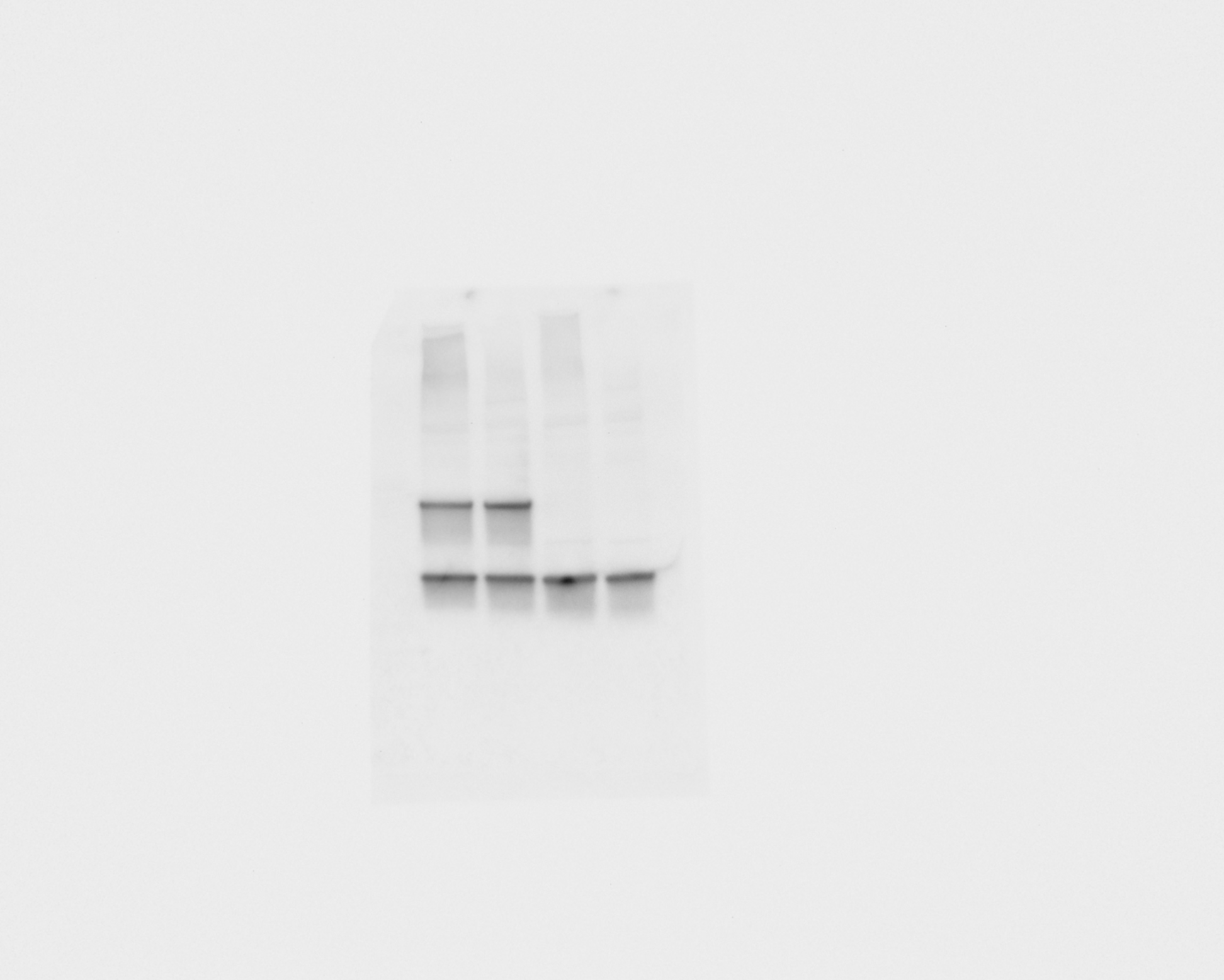

Supplement: Figure 2—figure supplement 1—source data 1. [file elife-110044-fig2-figsupp1-data1.zip › Figure 2 Supplement 1-source data 1/Figure 2 Supplement 1-b2.tif]

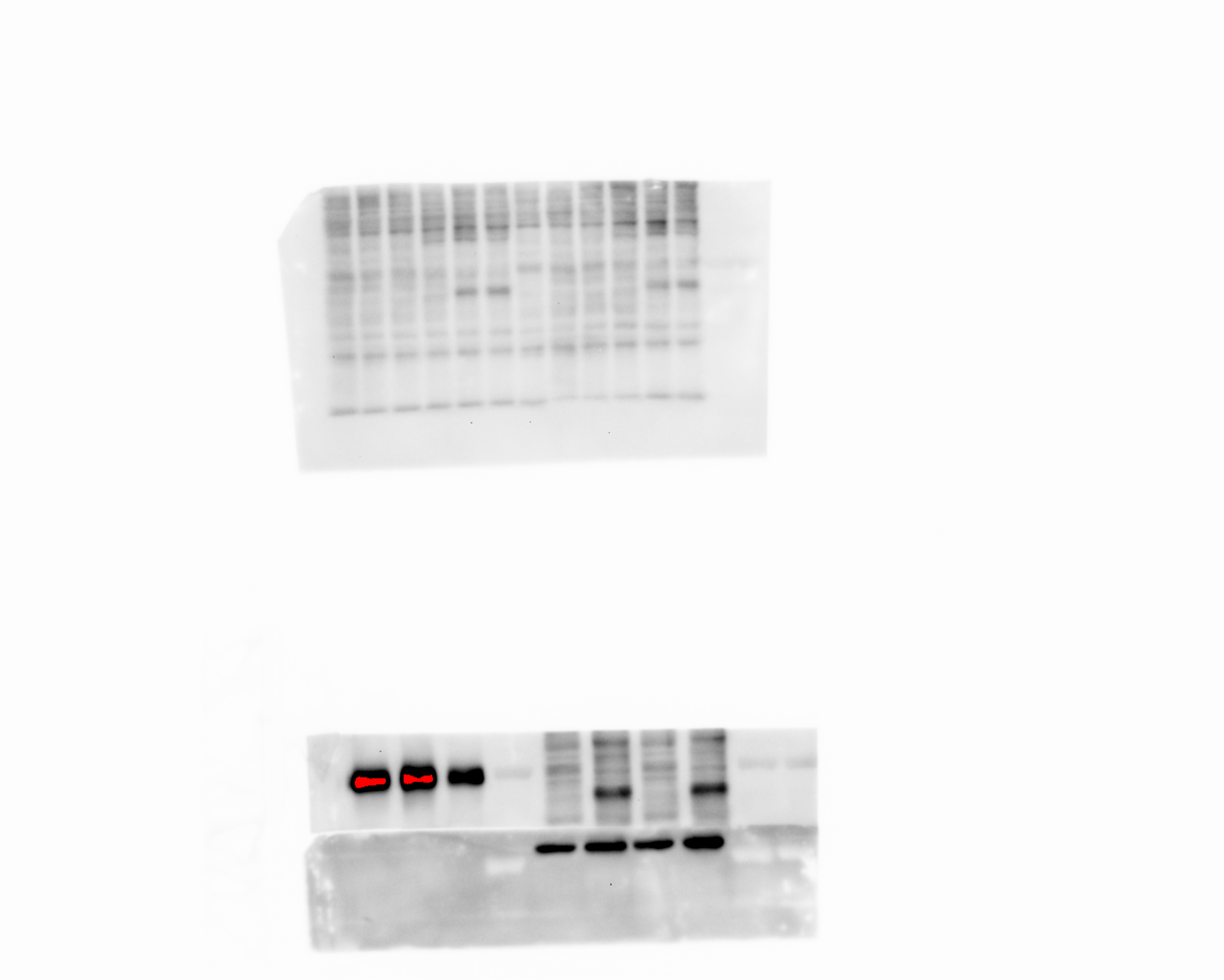

Supplement: Figure 2—figure supplement 1—source data 1. [file elife-110044-fig2-figsupp1-data1.zip › Figure 2 Supplement 1-source data 1/Figure 2 Supplement 1-b1.tif]

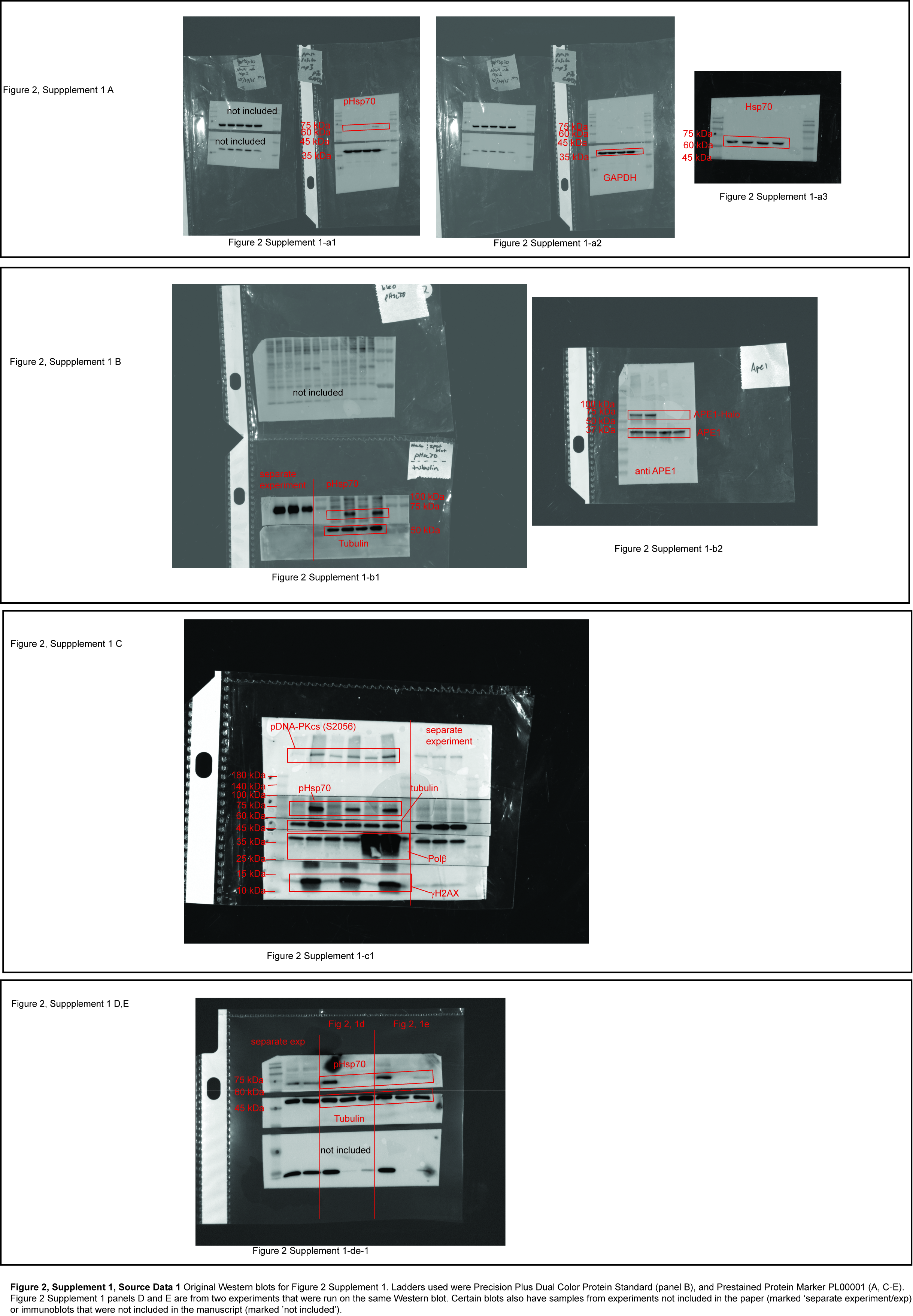

Supplement: Figure 2—figure supplement 1—source data 2. [file elife-110044-fig2-figsupp1-data2.zip › Figure 2 Supplement 1-source data 2/Figure 2 Supplement 1 - Source Data 2.tif]

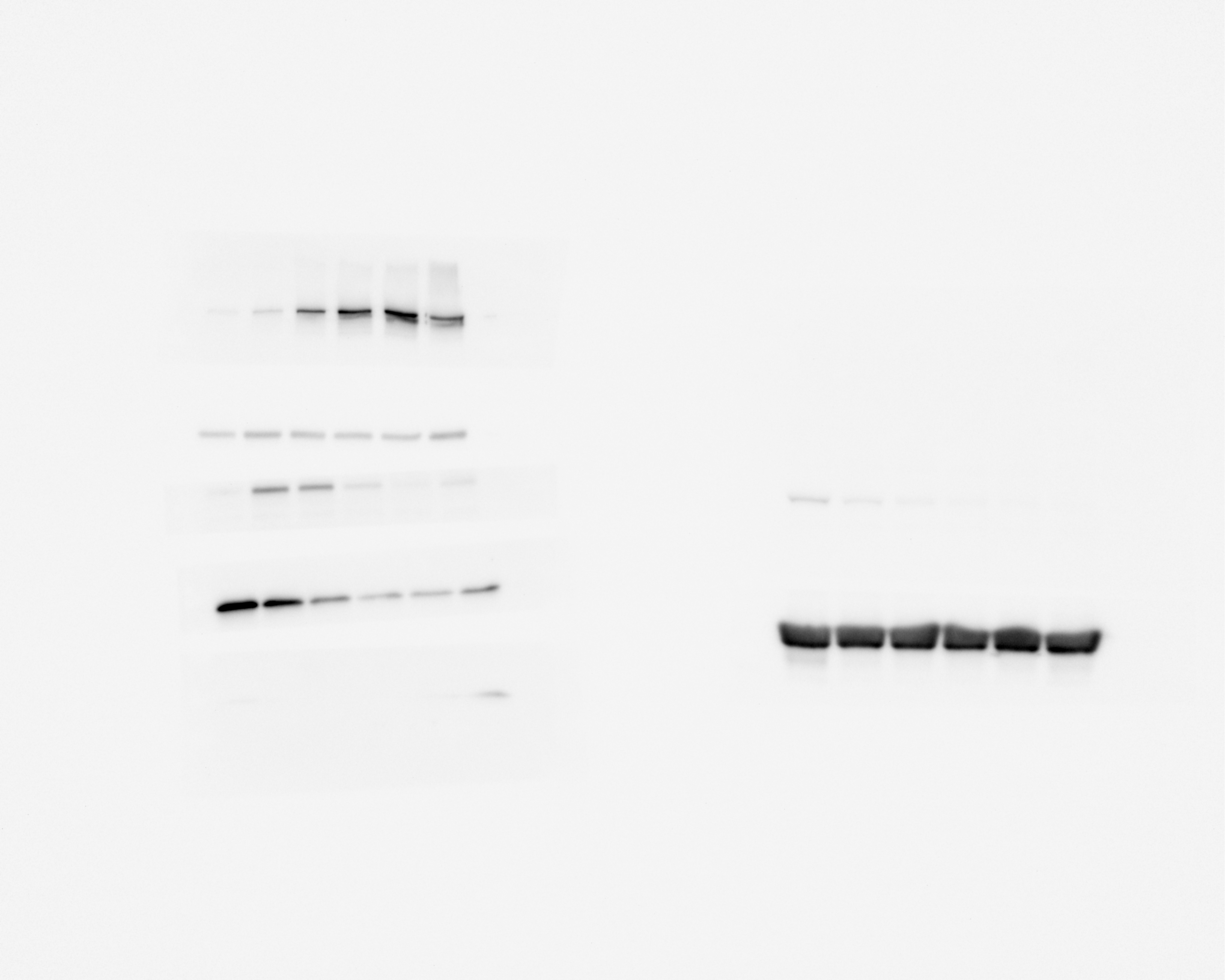

Supplement: Figure 3—source data 1. [file elife-110044-fig3-data1.zip › Figure 3-source data 1/Figure 3d-5.tif]

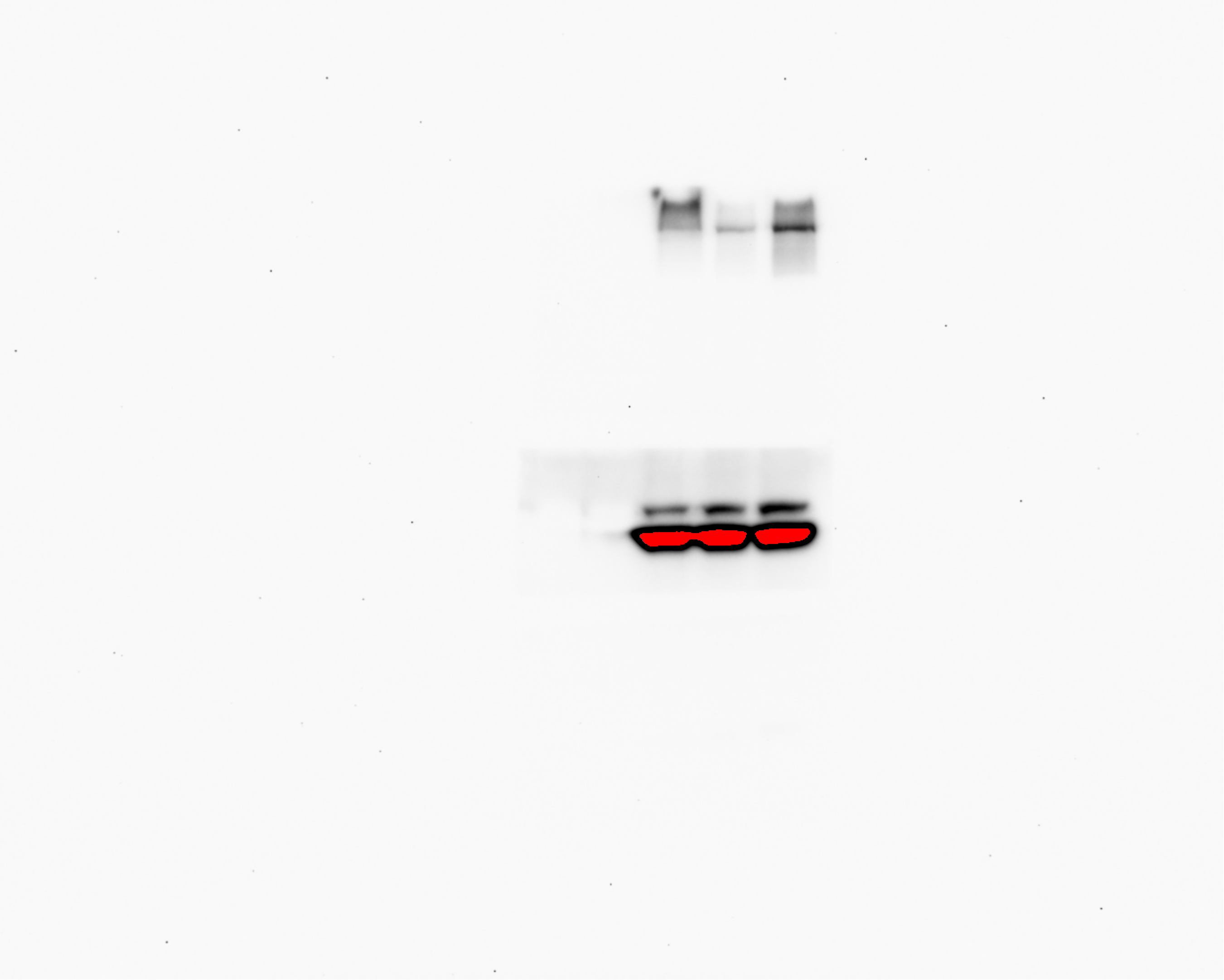

Supplement: Figure 3—source data 1. [file elife-110044-fig3-data1.zip › Figure 3-source data 1/Figure 3b-3.tif]

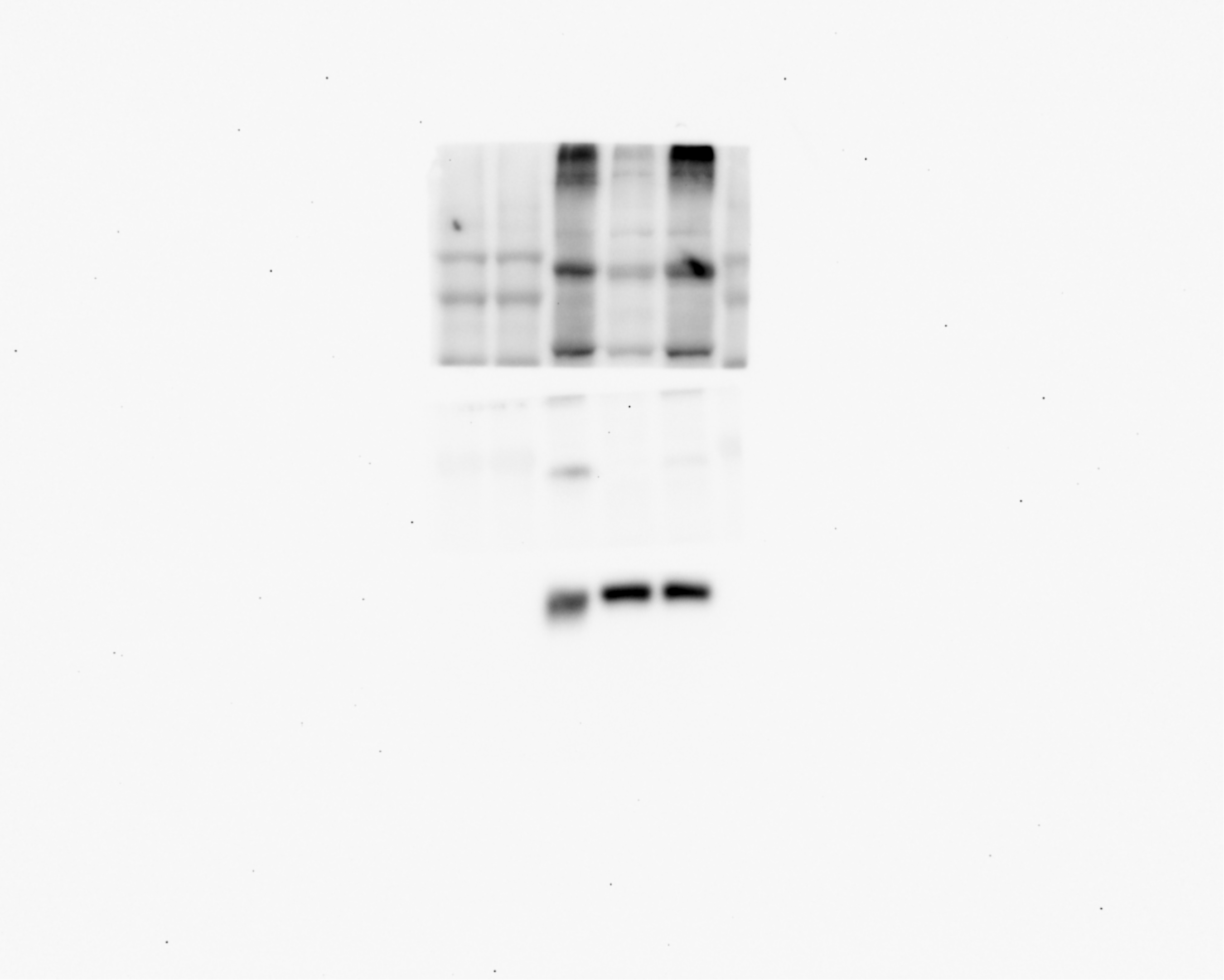

Supplement: Figure 3—source data 1. [file elife-110044-fig3-data1.zip › Figure 3-source data 1/Figure 3b-2.tif]

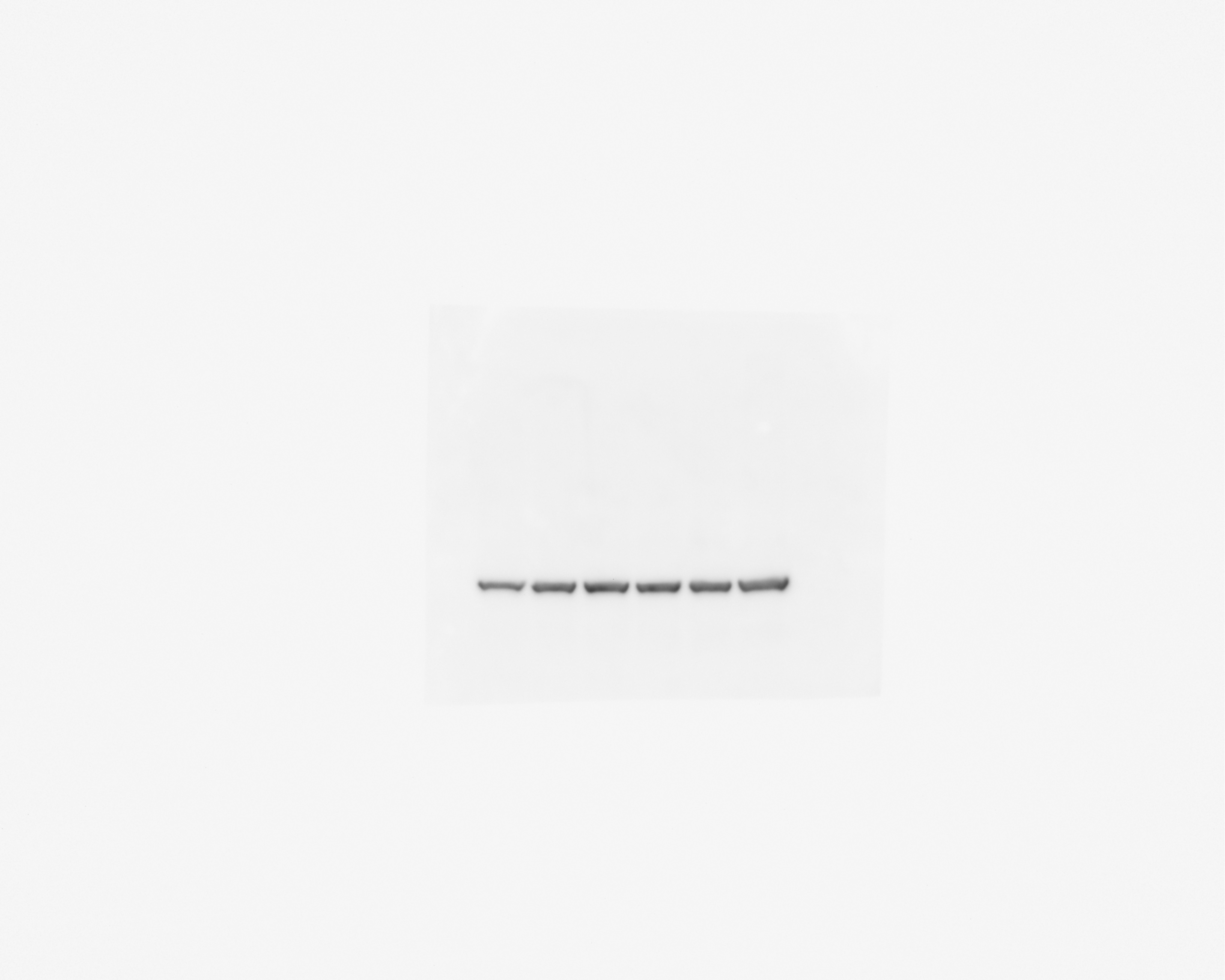

Supplement: Figure 3—source data 1. [file elife-110044-fig3-data1.zip › Figure 3-source data 1/Figure 3d-4.tif]

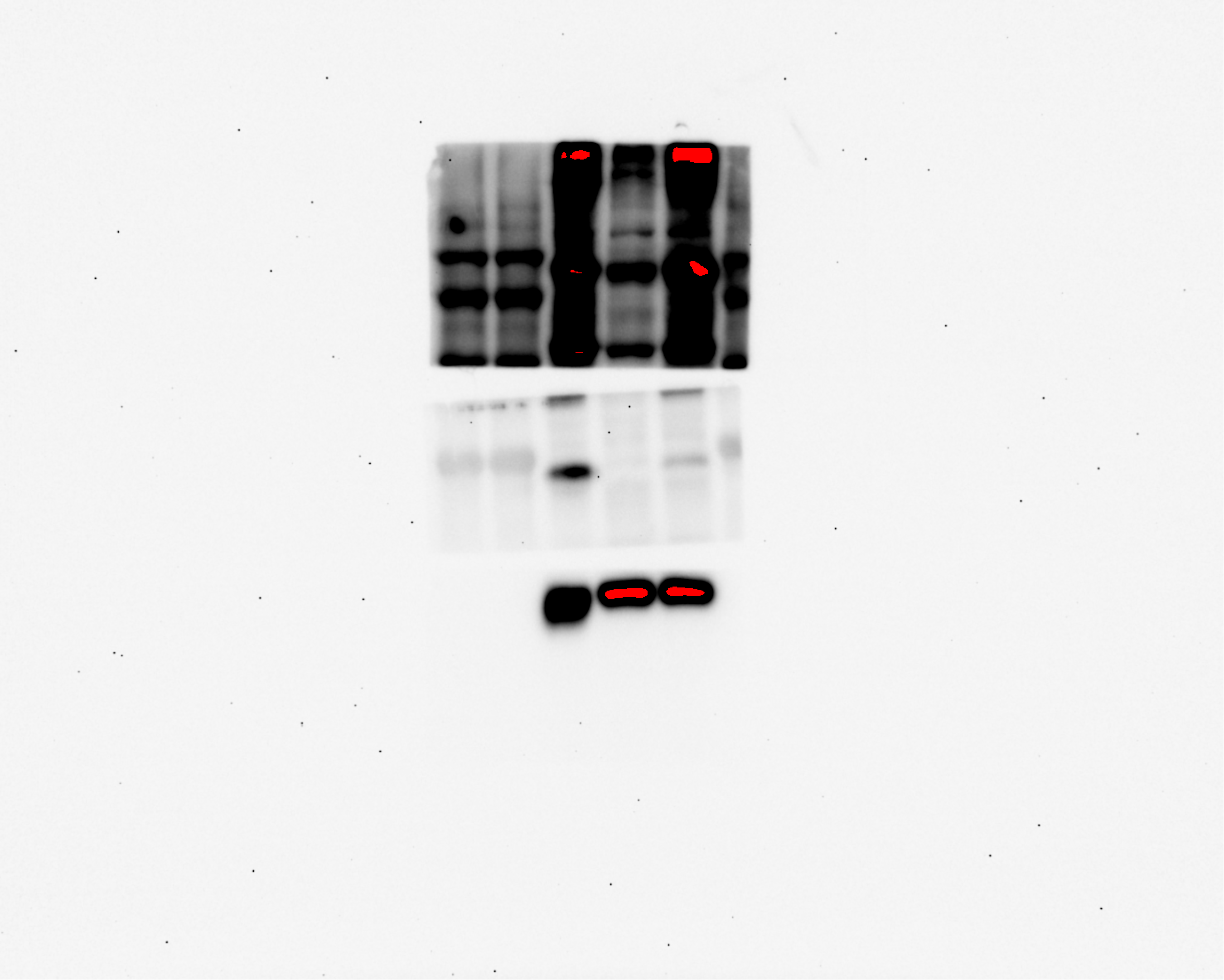

Supplement: Figure 3—source data 1. [file elife-110044-fig3-data1.zip › Figure 3-source data 1/Figure 3b-1.tif]

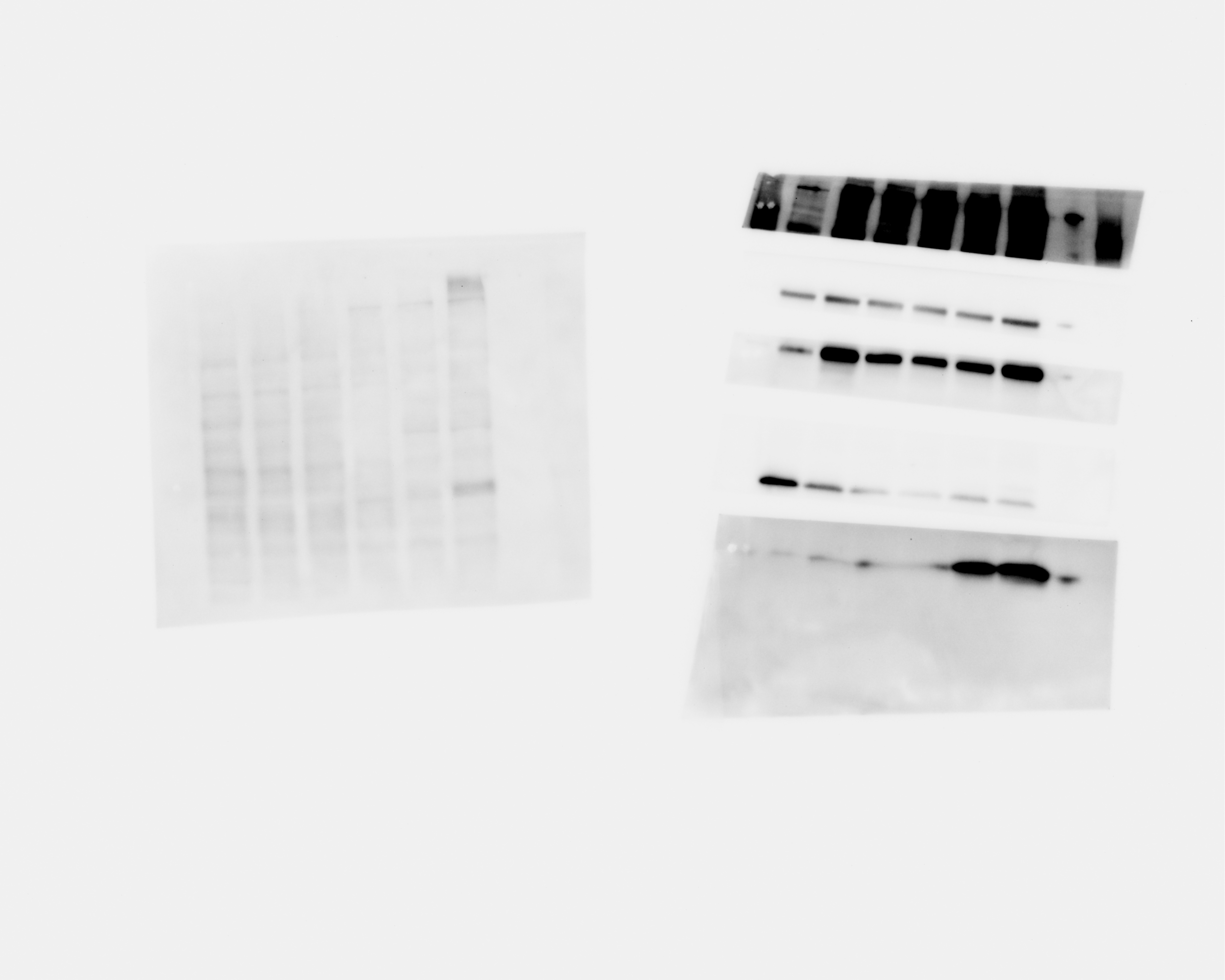

Supplement: Figure 3—source data 1. [file elife-110044-fig3-data1.zip › Figure 3-source data 1/Figure 3d-3.tif]

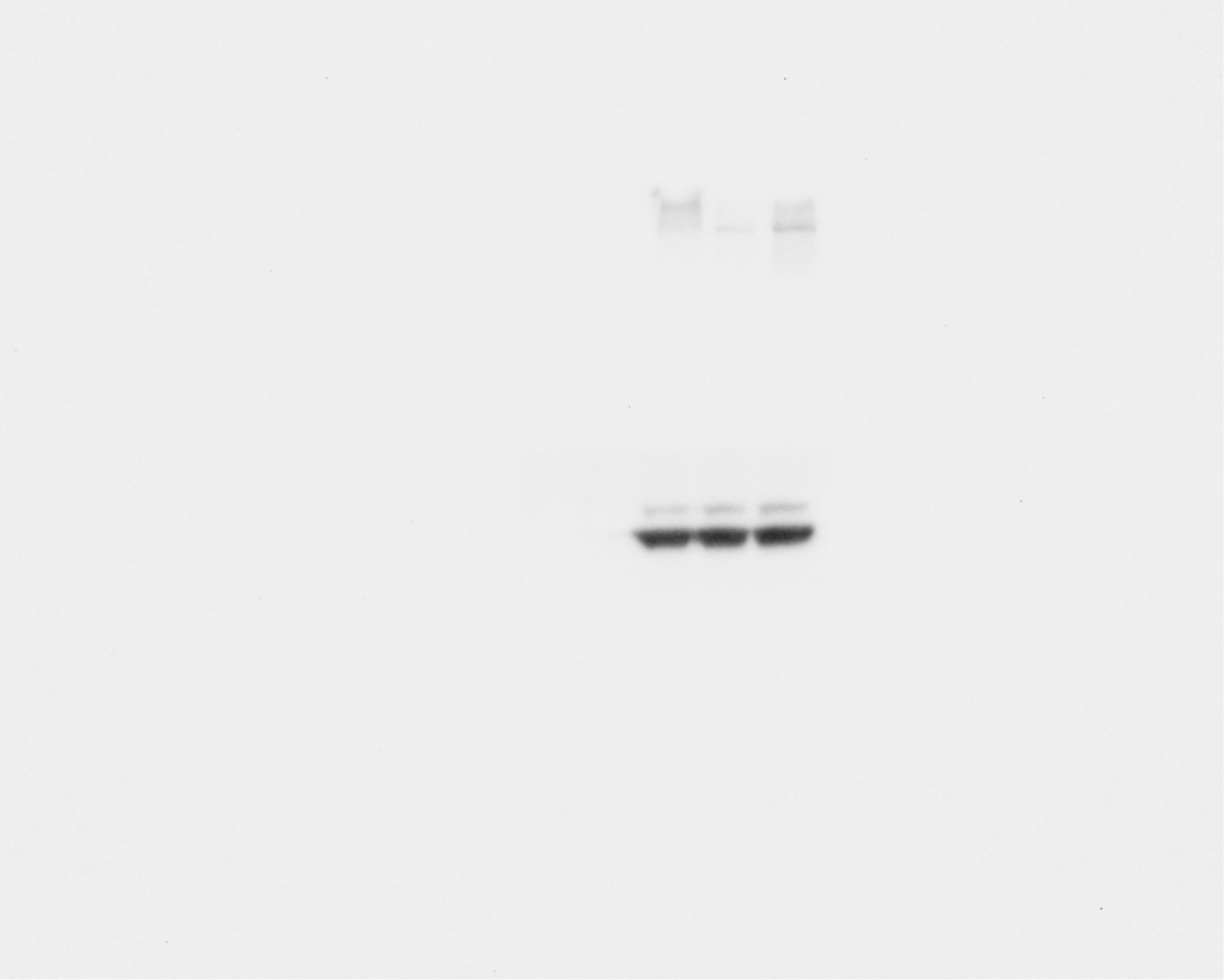

Supplement: Figure 3—source data 1. [file elife-110044-fig3-data1.zip › Figure 3-source data 1/Figure 3b-4.tif]

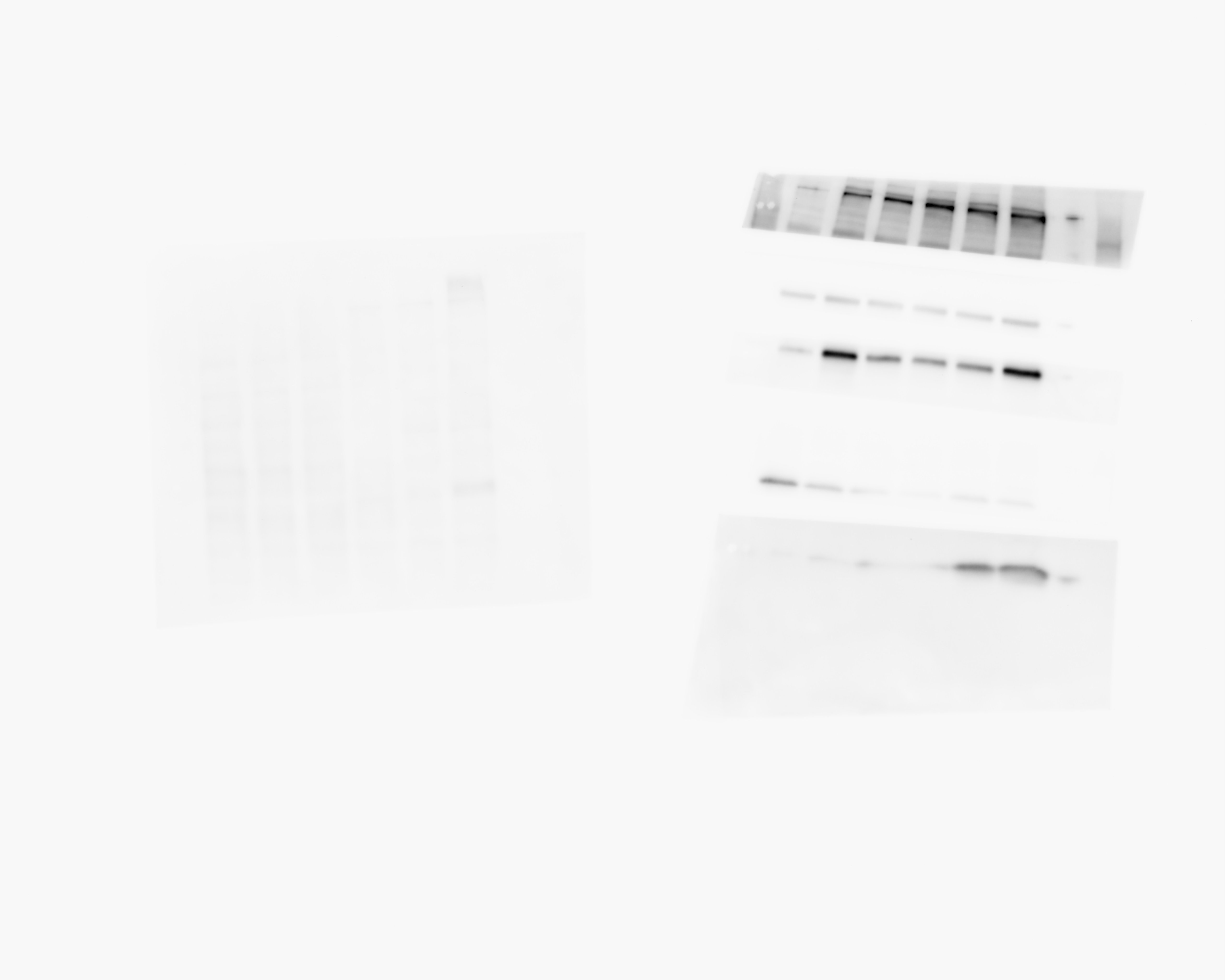

Supplement: Figure 3—source data 1. [file elife-110044-fig3-data1.zip › Figure 3-source data 1/Figure 3d-2.tif]

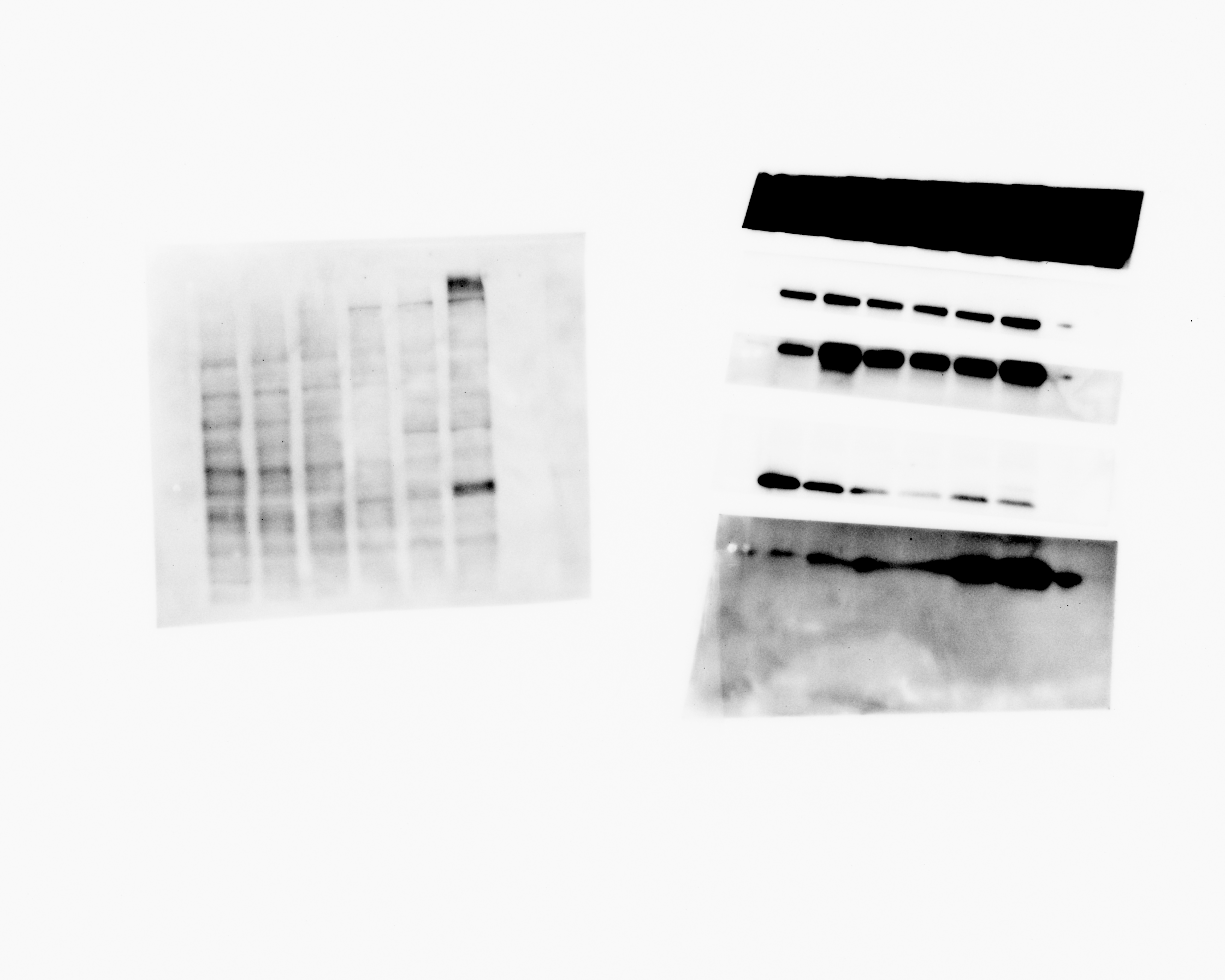

Supplement: Figure 3—source data 1. [file elife-110044-fig3-data1.zip › Figure 3-source data 1/Figure 3d-1.tif]

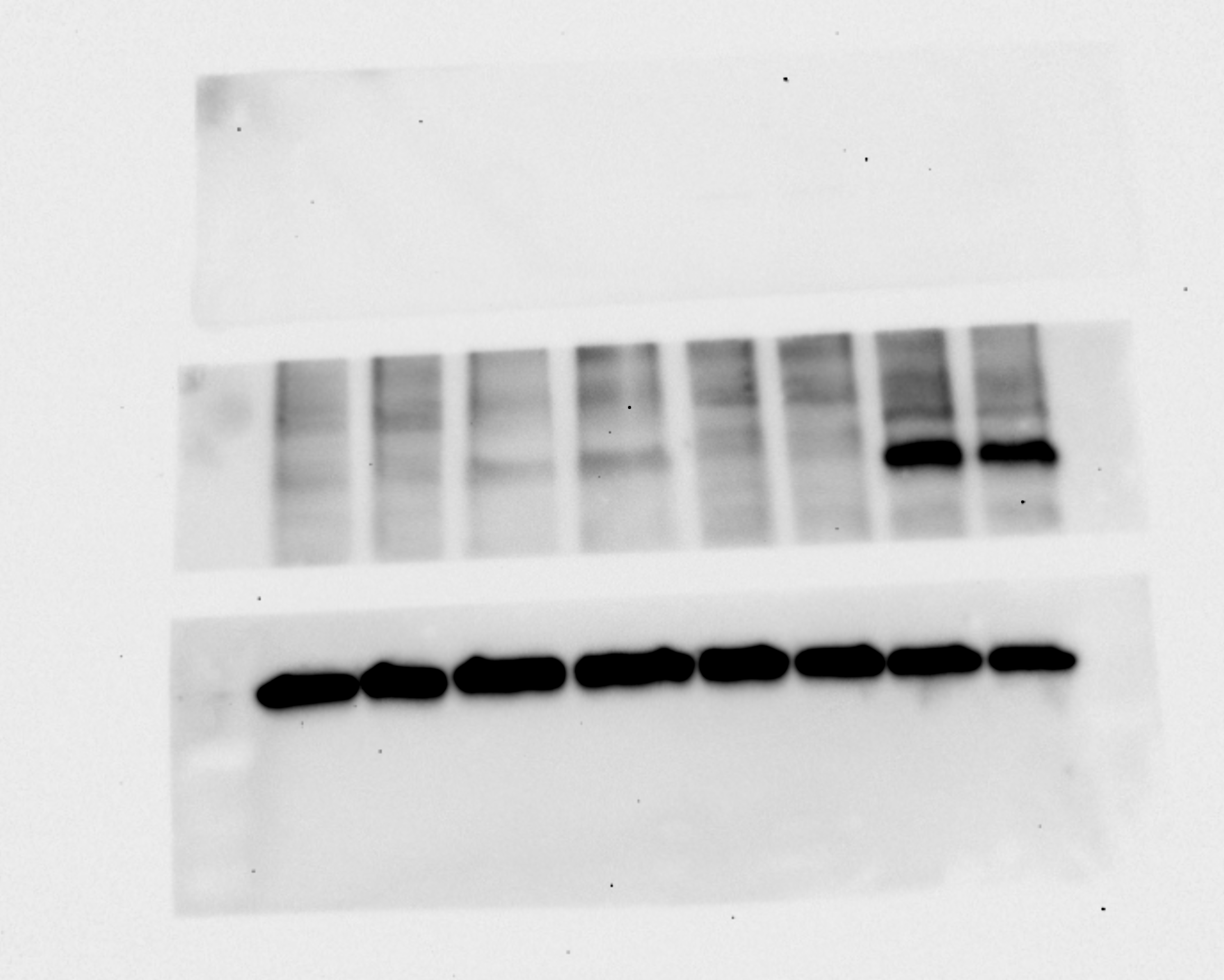

Supplement: Figure 3—source data 1. [file elife-110044-fig3-data1.zip › Figure 3-source data 1/Figure 3a-2.tif]

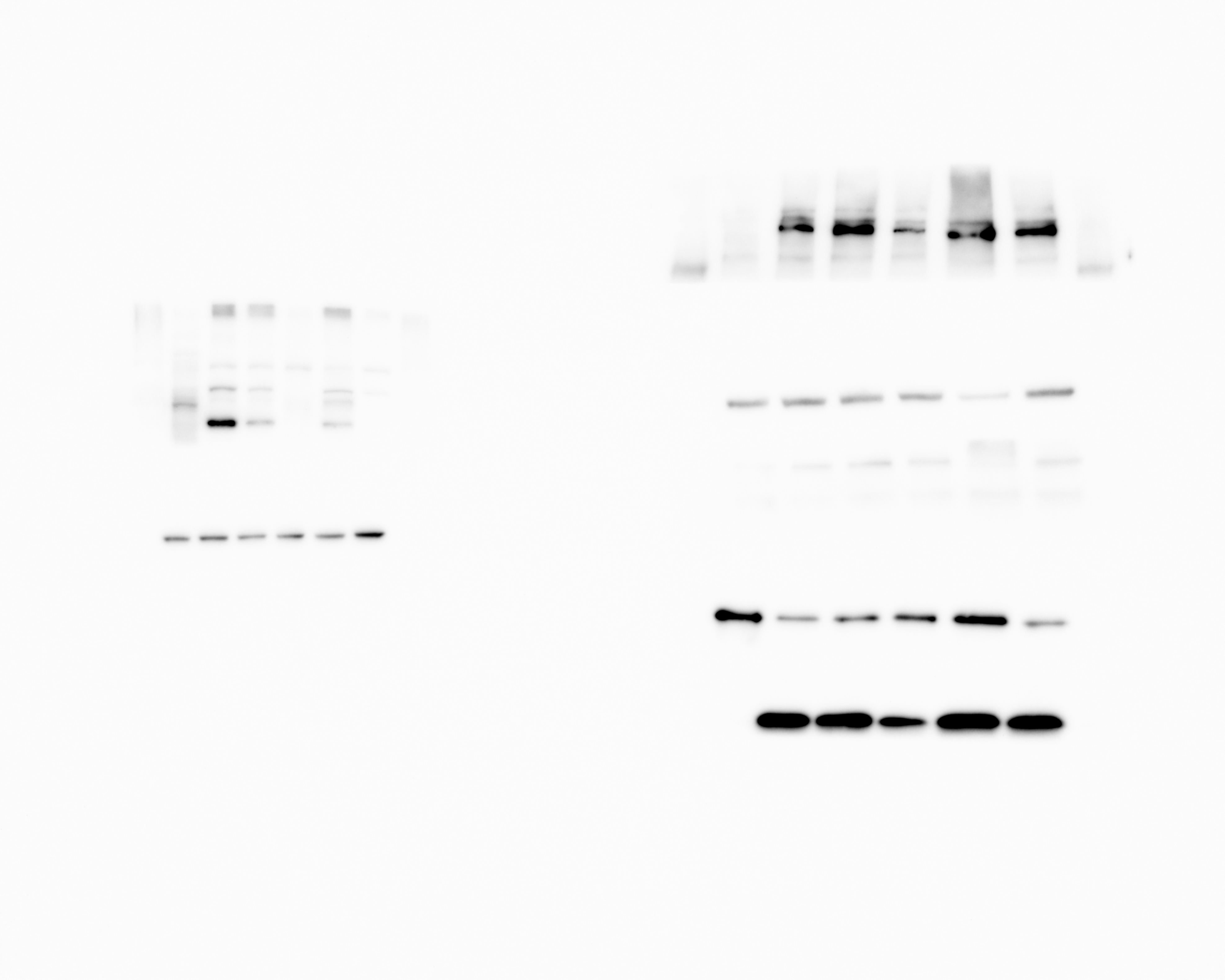

Supplement: Figure 3—source data 1. [file elife-110044-fig3-data1.zip › Figure 3-source data 1/Figure 3c-1.tif]

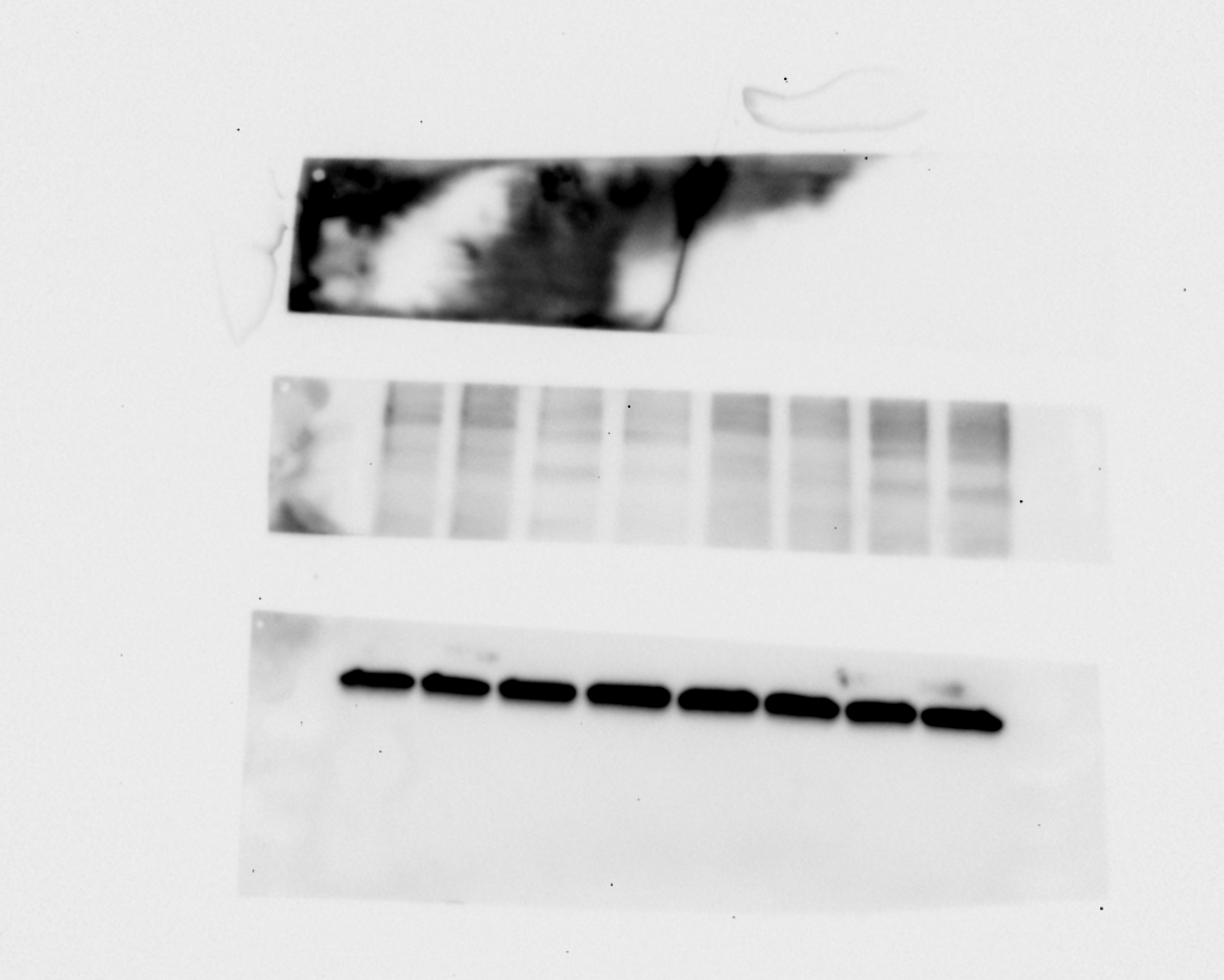

Supplement: Figure 3—source data 1. [file elife-110044-fig3-data1.zip › Figure 3-source data 1/Figure 3a-1.tif]

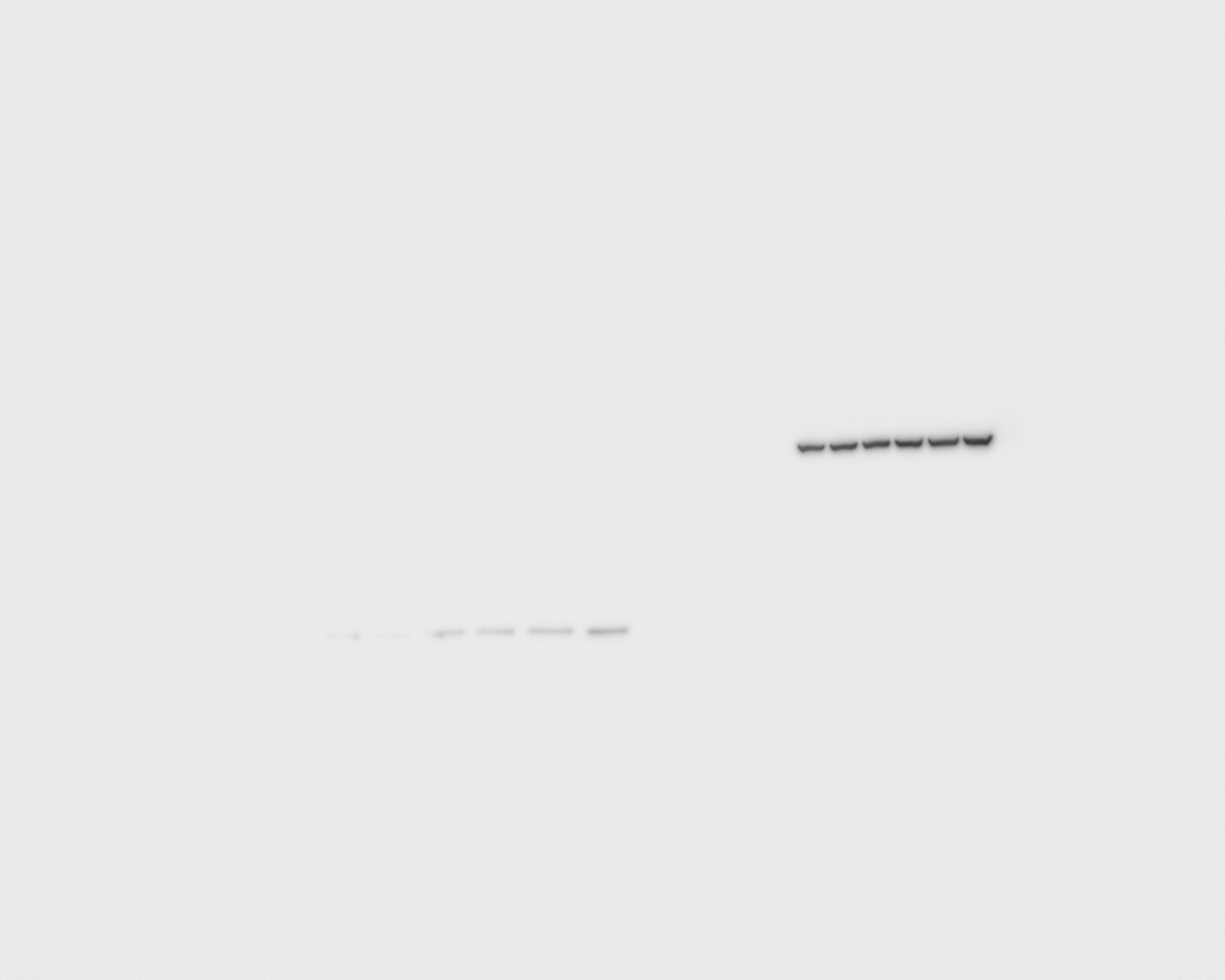

Supplement: Figure 3—source data 1. [file elife-110044-fig3-data1.zip › Figure 3-source data 1/Figure 3c-2.tif]

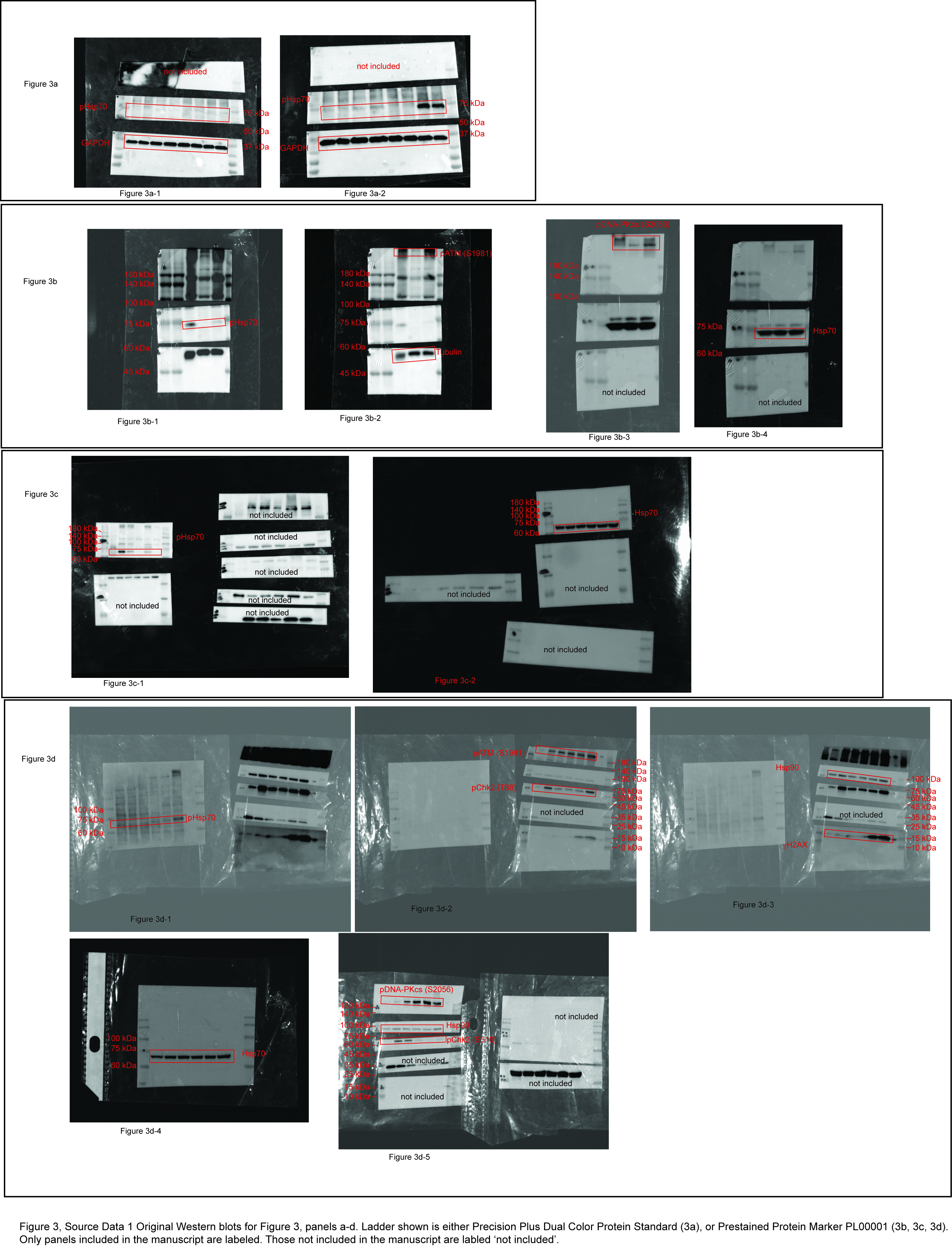

Supplement: Figure 3—source data 2. [file elife-110044-fig3-data2.zip › Figure 3-source data 2/Fig3-source data 2.tif]

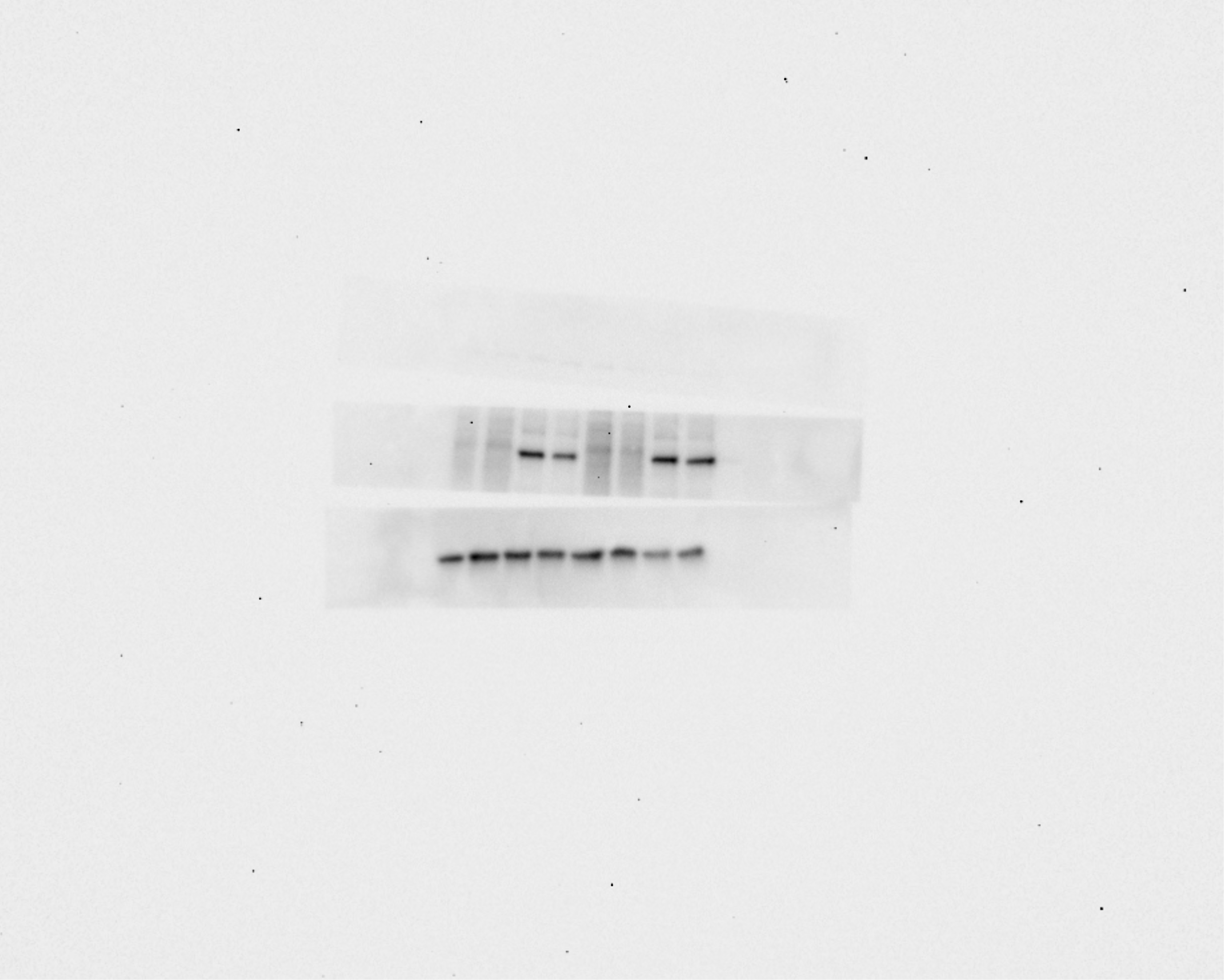

Supplement: Figure 3—figure supplement 1—source data 1. [file elife-110044-fig3-figsupp1-data1.zip › Figure 3 Supplement 1-source data 1/Figure 3 Supplement 1-a1.tif]

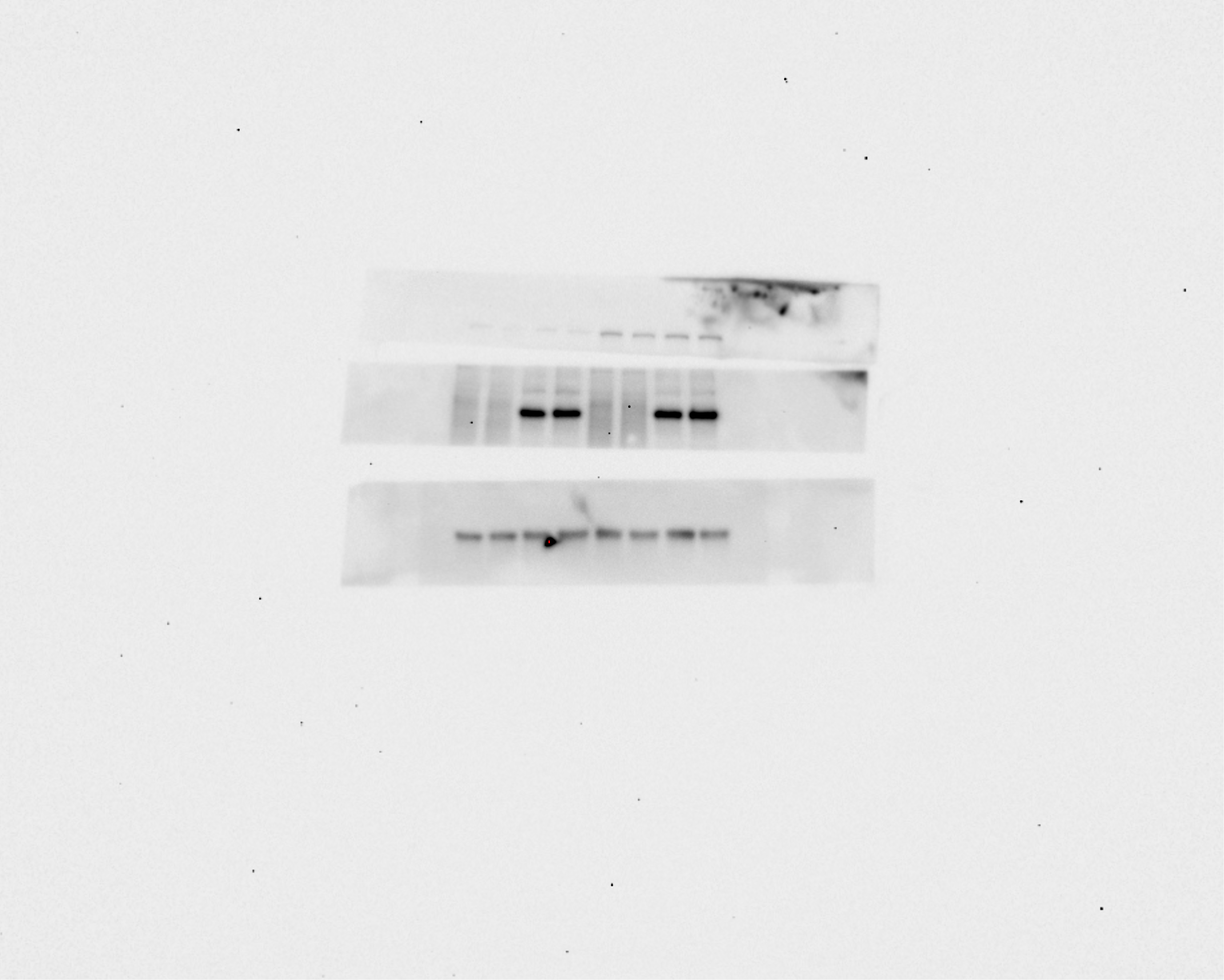

Supplement: Figure 3—figure supplement 1—source data 1. [file elife-110044-fig3-figsupp1-data1.zip › Figure 3 Supplement 1-source data 1/Figure 3 Supplement 1-a2.tif]

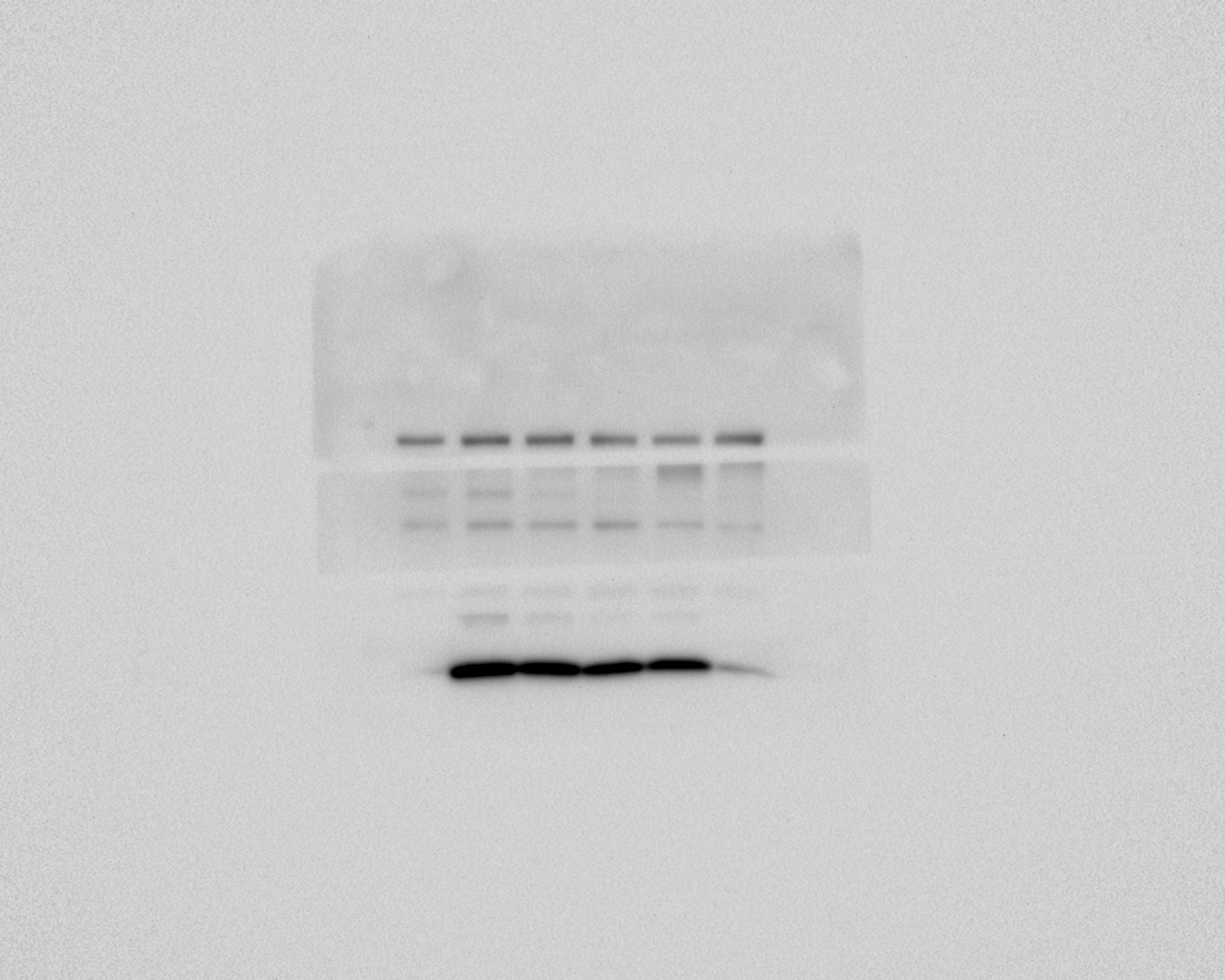

Supplement: Figure 3—figure supplement 1—source data 1. [file elife-110044-fig3-figsupp1-data1.zip › Figure 3 Supplement 1-source data 1/Figure 3 Supplement 1-d5.tif]

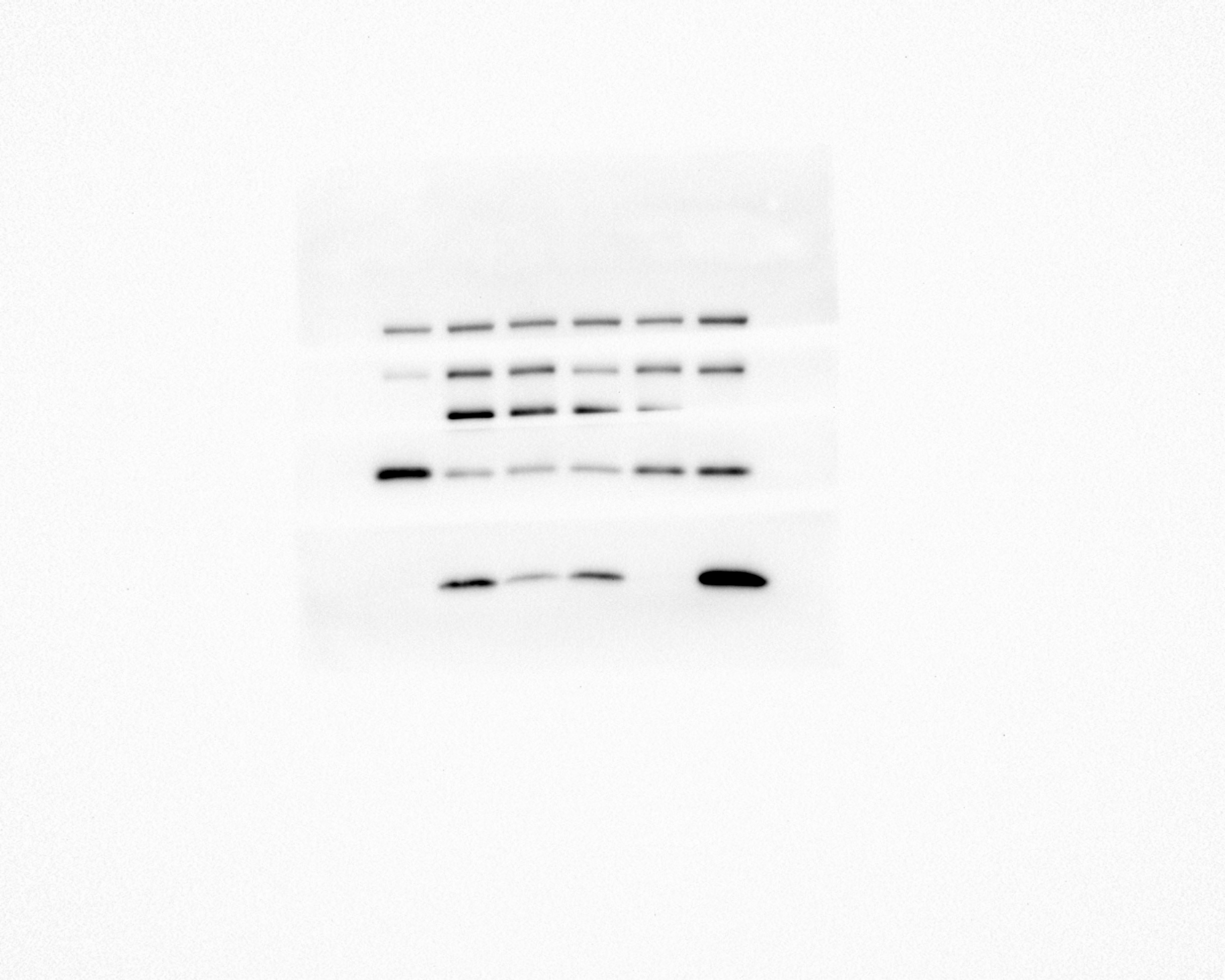

Supplement: Figure 3—figure supplement 1—source data 1. [file elife-110044-fig3-figsupp1-data1.zip › Figure 3 Supplement 1-source data 1/Figure 3 Supplement 1-d4.tif]

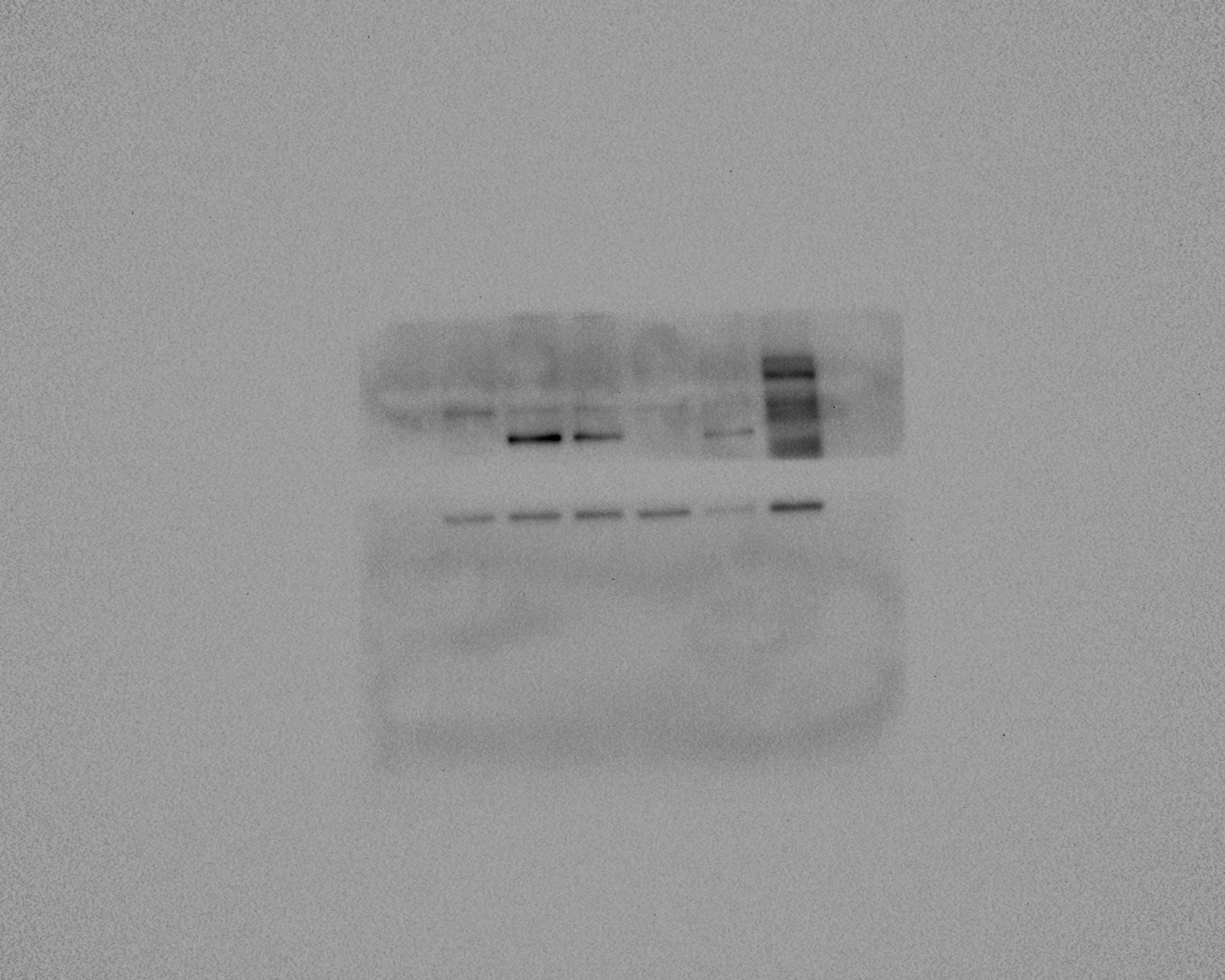

Supplement: Figure 3—figure supplement 1—source data 1. [file elife-110044-fig3-figsupp1-data1.zip › Figure 3 Supplement 1-source data 1/Figure 3 Supplement 1-d1.tif]

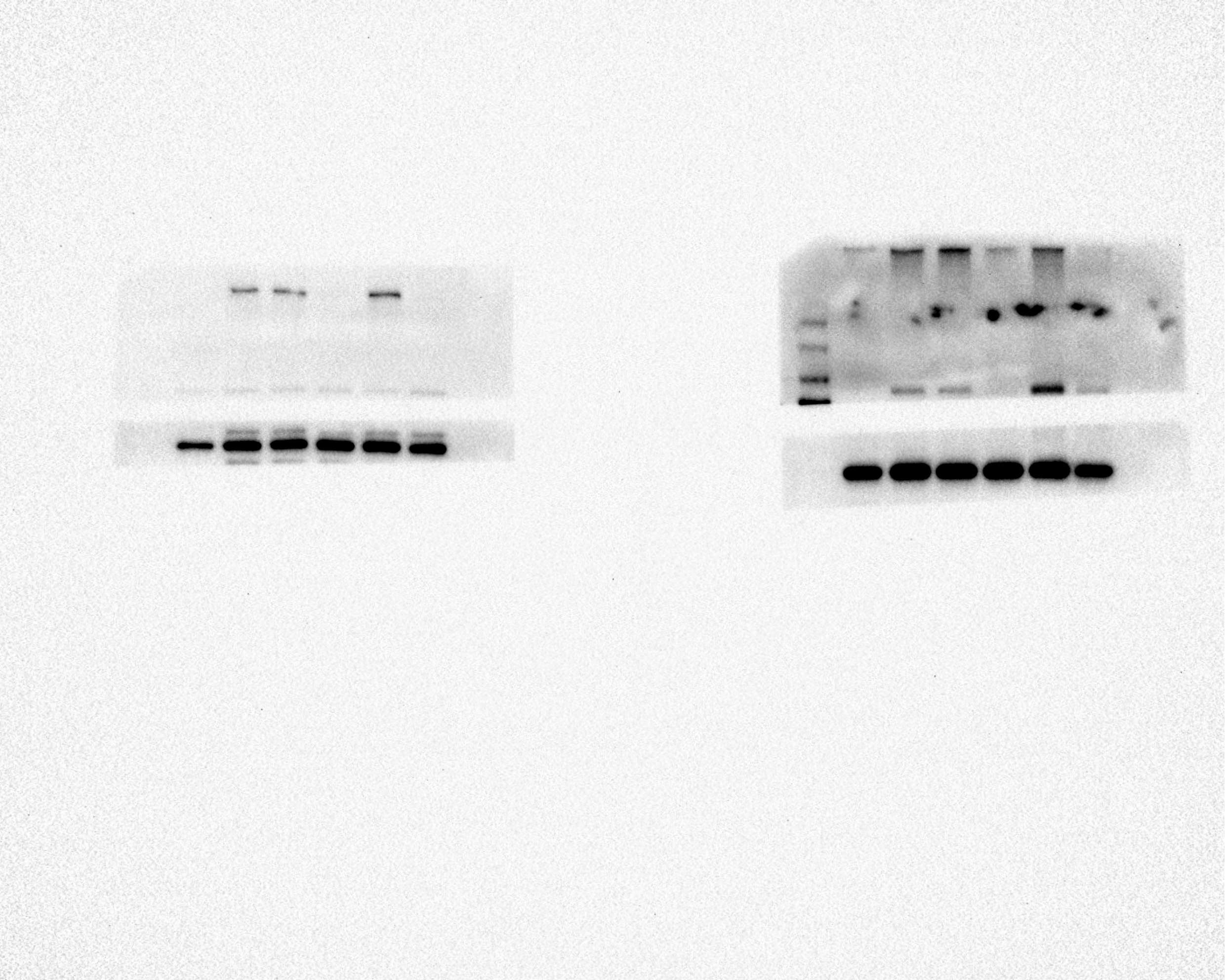

Supplement: Figure 3—figure supplement 1—source data 1. [file elife-110044-fig3-figsupp1-data1.zip › Figure 3 Supplement 1-source data 1/Figure 3 Supplement 1-d3.tif]

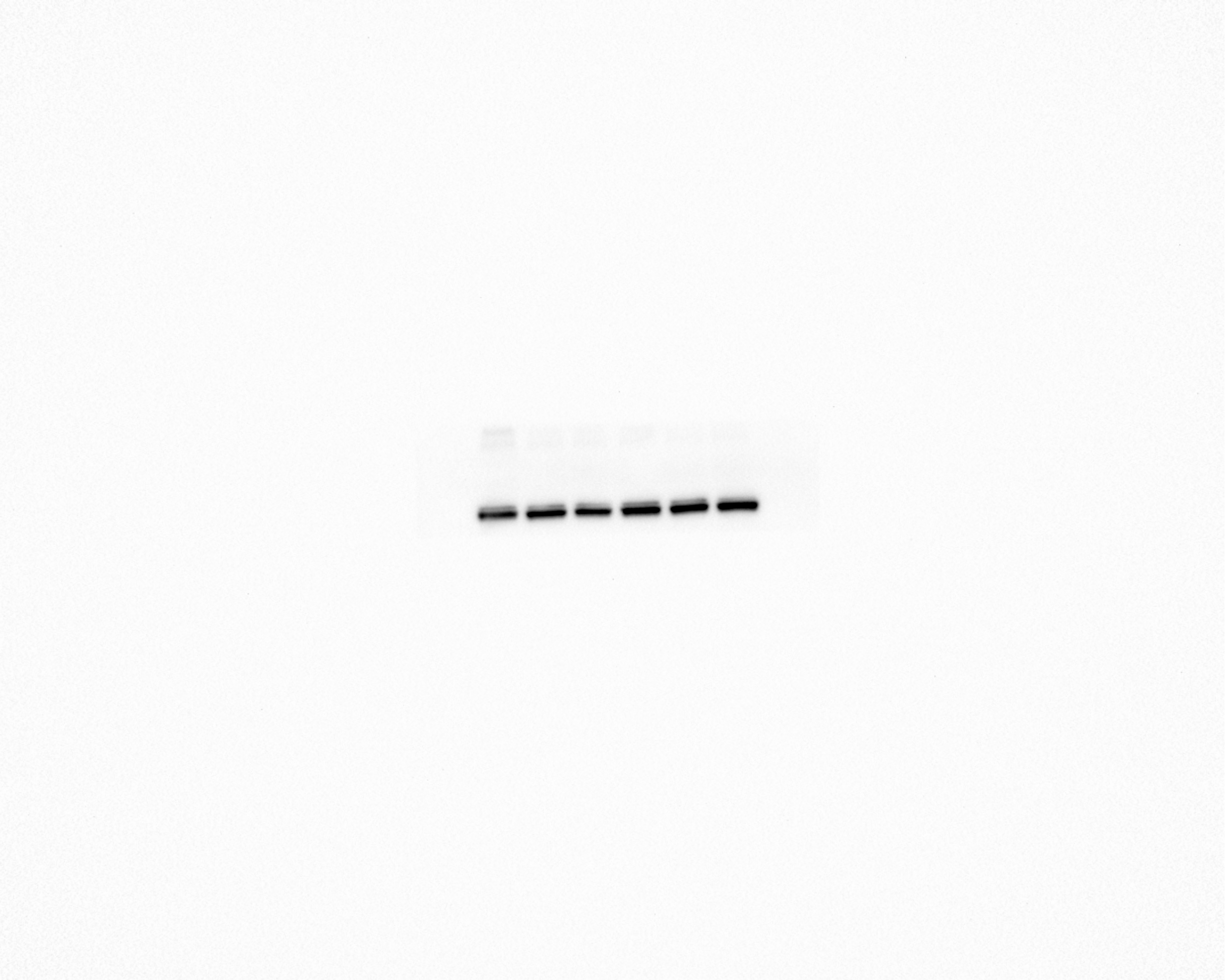

Supplement: Figure 3—figure supplement 1—source data 1. [file elife-110044-fig3-figsupp1-data1.zip › Figure 3 Supplement 1-source data 1/Figure 3 Supplement 1-d2.tif]

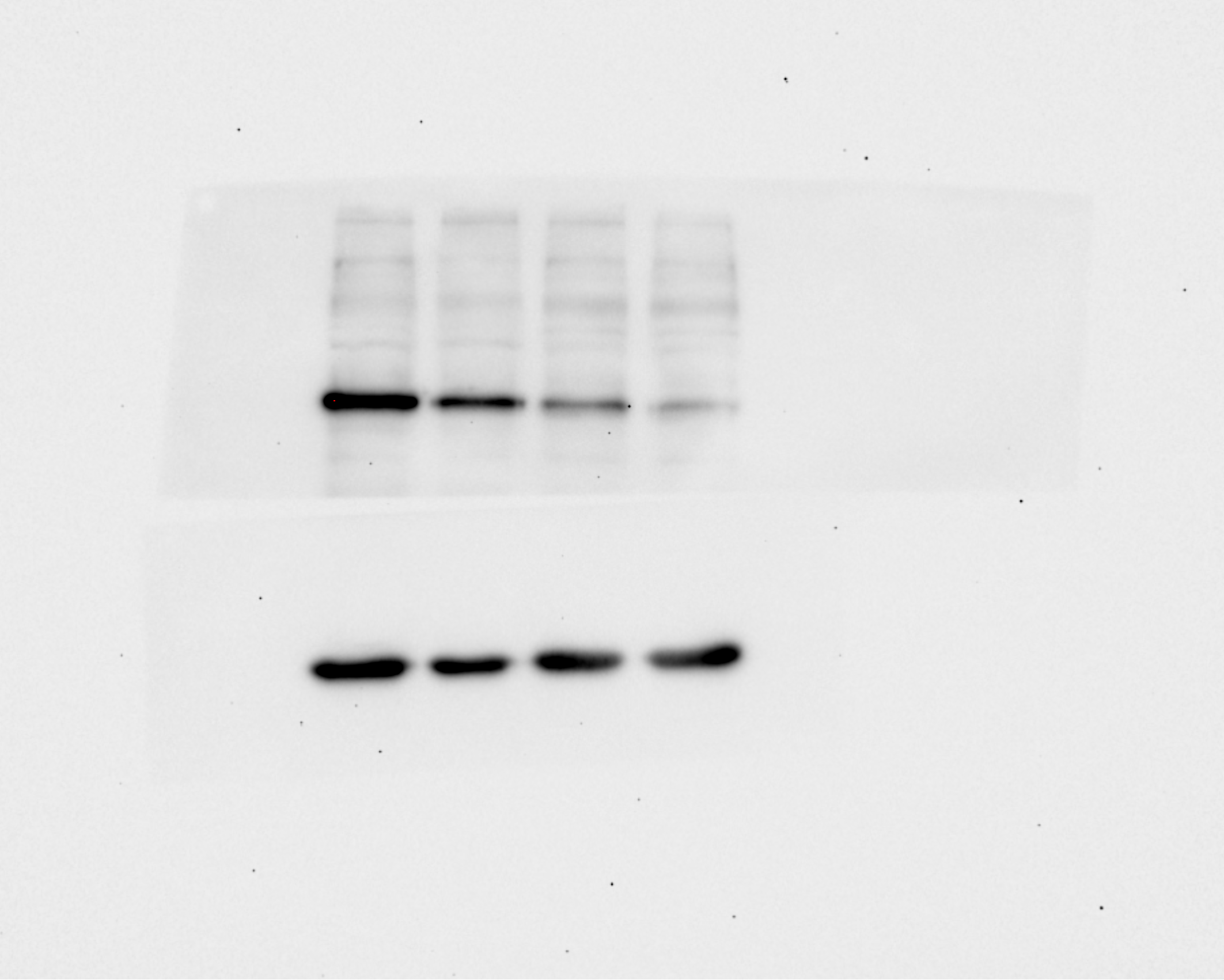

Supplement: Figure 3—figure supplement 1—source data 1. [file elife-110044-fig3-figsupp1-data1.zip › Figure 3 Supplement 1-source data 1/Figure 3 Supplement 1-c1.tif]

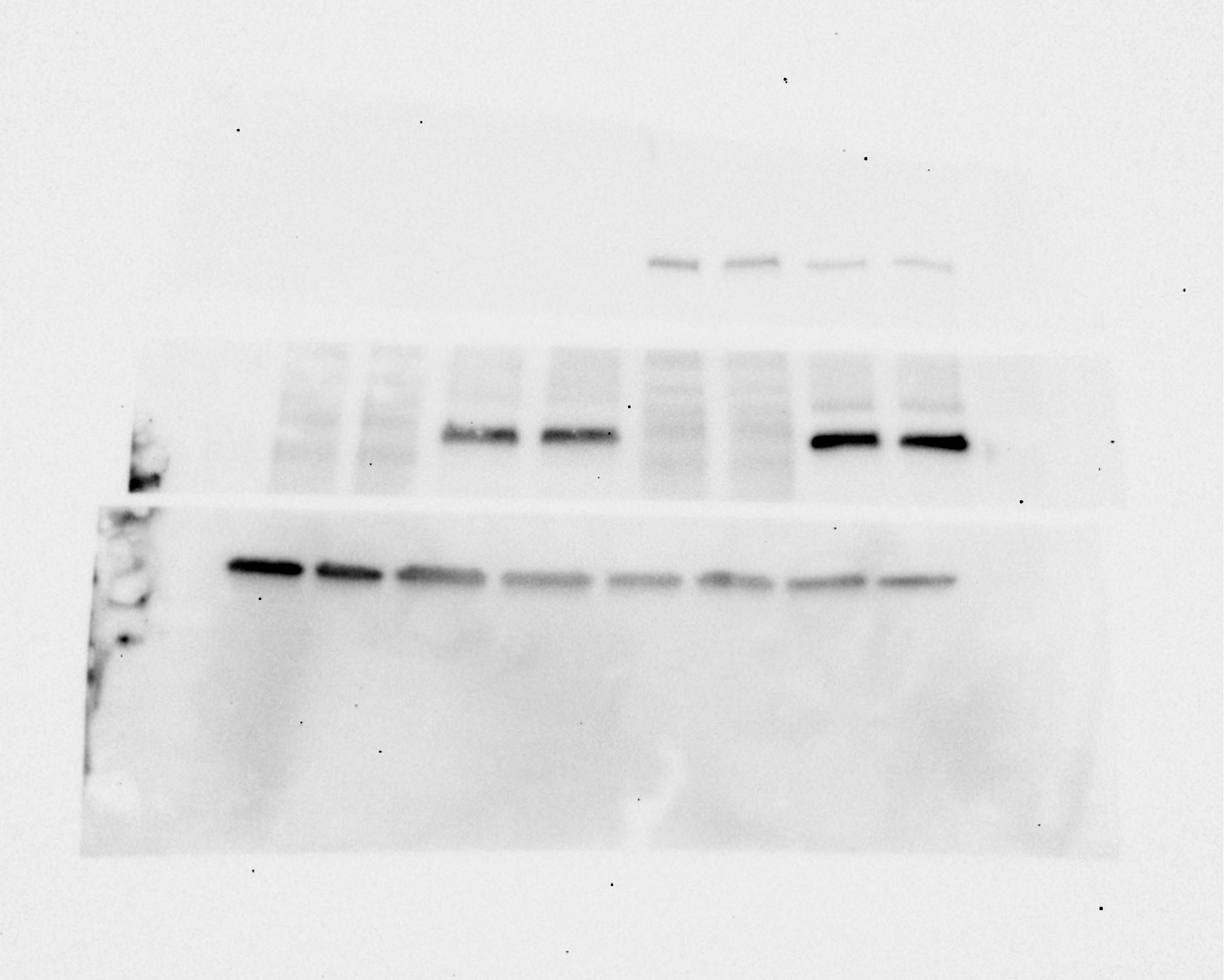

Supplement: Figure 3—figure supplement 1—source data 1. [file elife-110044-fig3-figsupp1-data1.zip › Figure 3 Supplement 1-source data 1/Figure 3 Supplement 1-b2.tif]

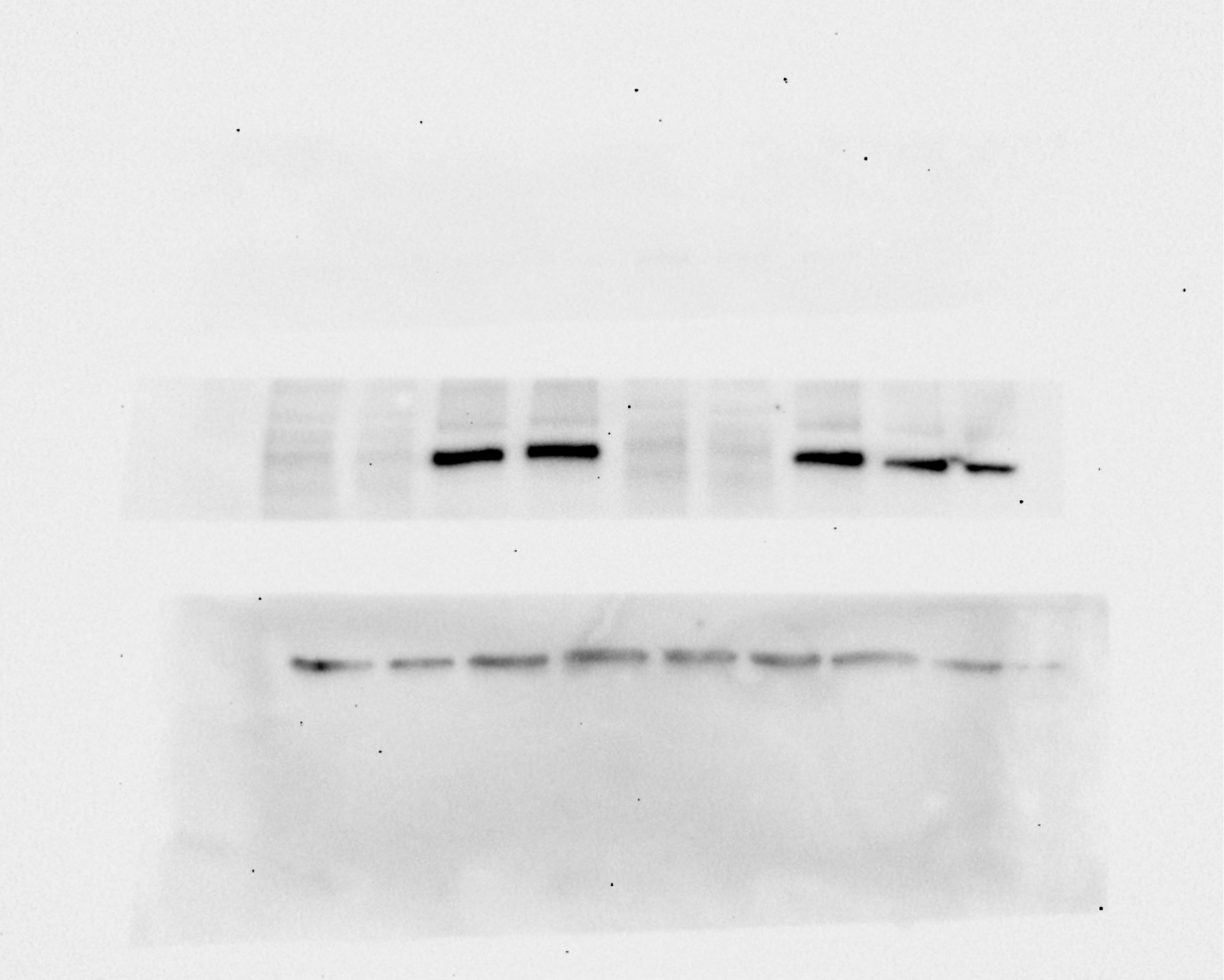

Supplement: Figure 3—figure supplement 1—source data 1. [file elife-110044-fig3-figsupp1-data1.zip › Figure 3 Supplement 1-source data 1/Figure 3 Supplement 1-b1.tif]

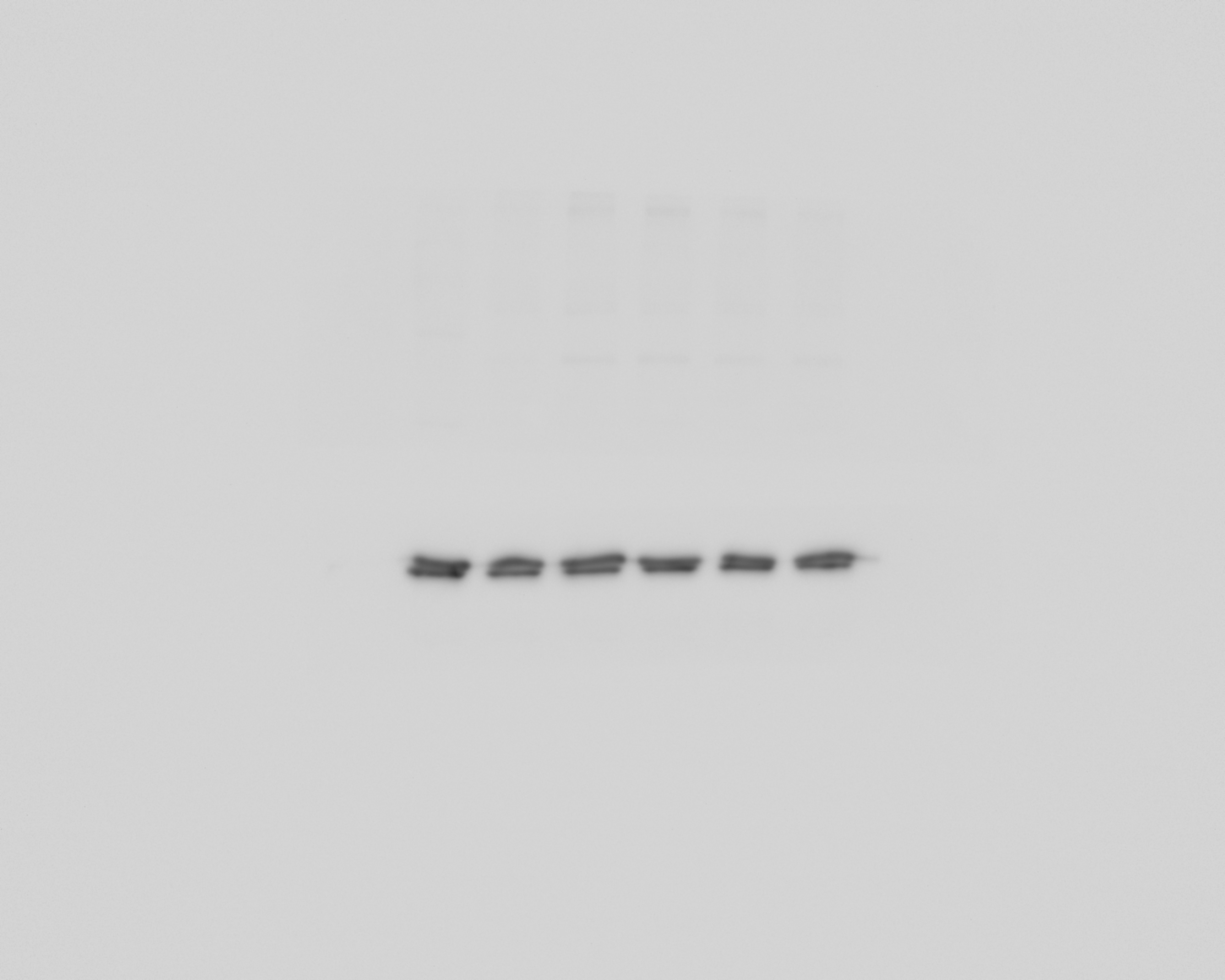

Supplement: Figure 4—source data 1. [file elife-110044-fig4-data1.zip › Figure 4-source data 1/Figure 4a-2.tif]

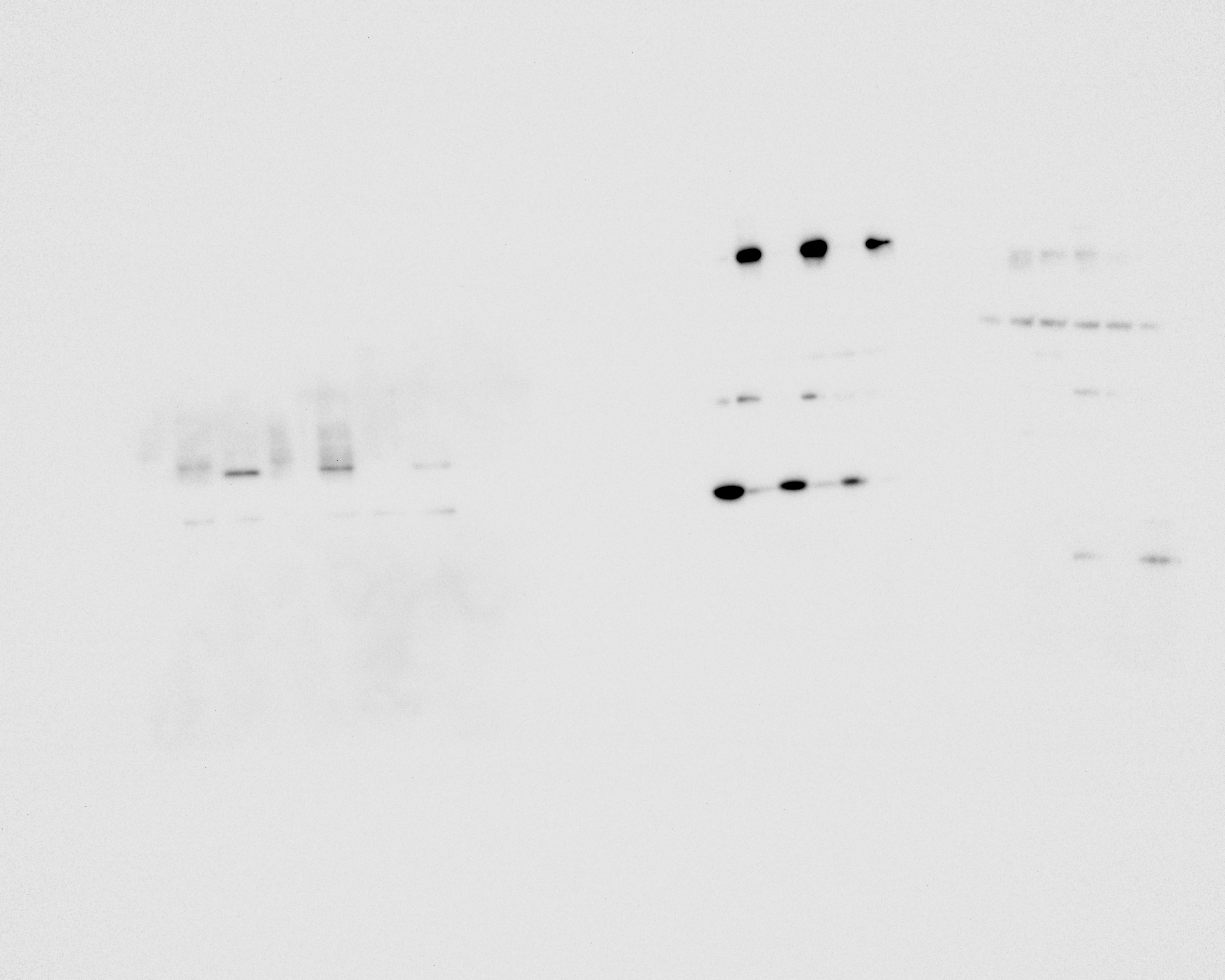

Supplement: Figure 4—source data 1. [file elife-110044-fig4-data1.zip › Figure 4-source data 1/Figure 4c-1.tif]

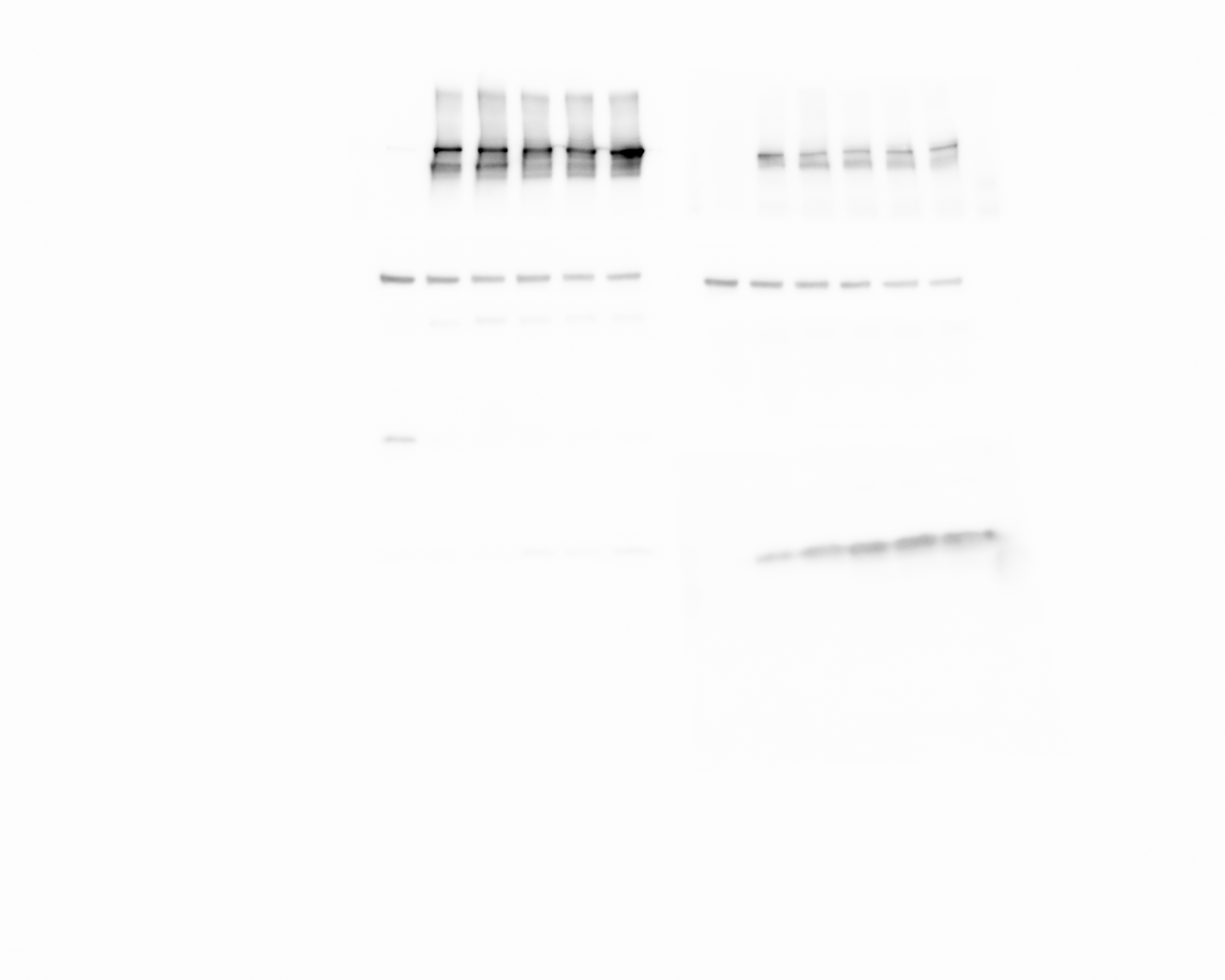

Supplement: Figure 4—source data 1. [file elife-110044-fig4-data1.zip › Figure 4-source data 1/Figure 4a-3.tif]

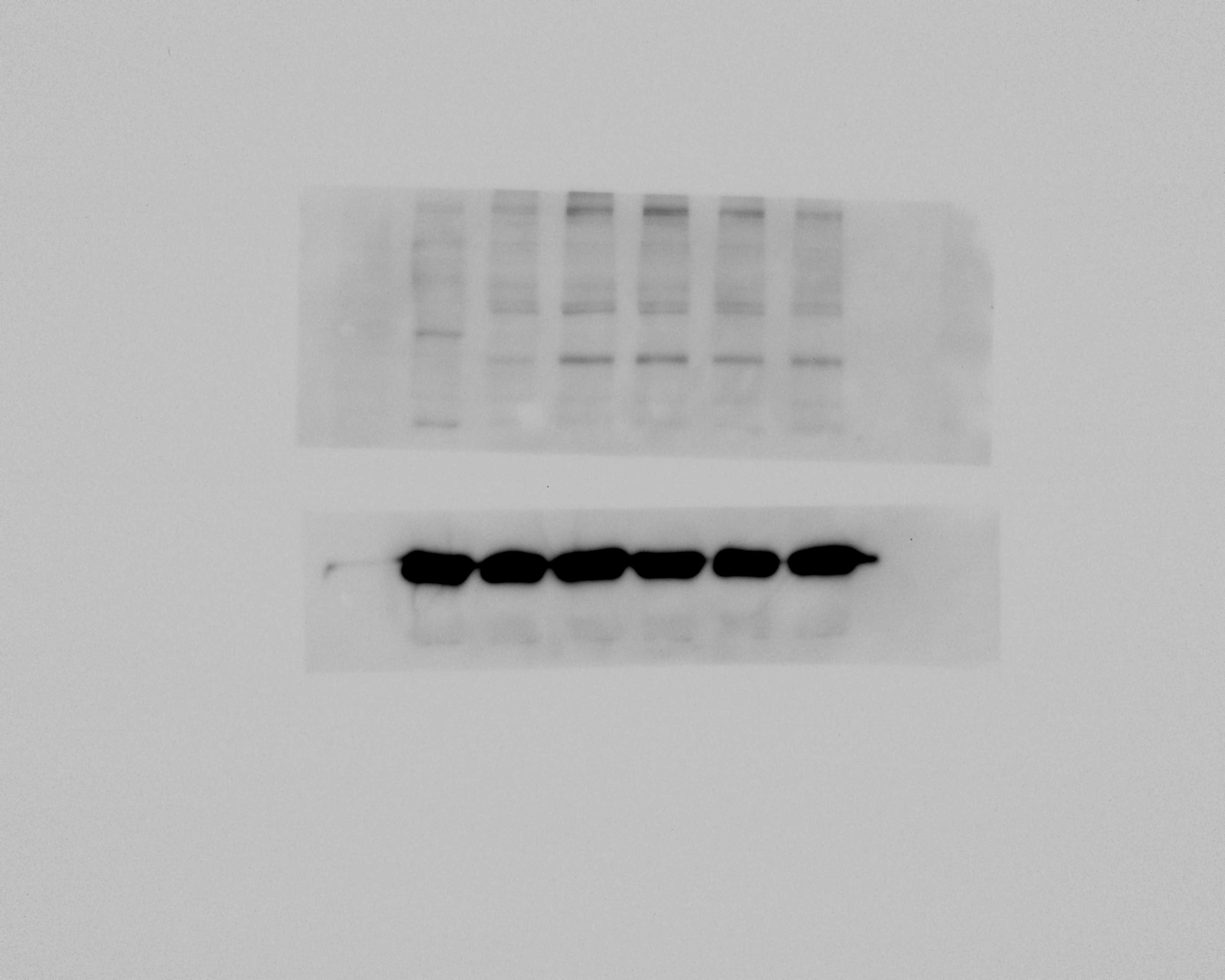

Supplement: Figure 4—source data 1. [file elife-110044-fig4-data1.zip › Figure 4-source data 1/Figure 4a-1.tif]

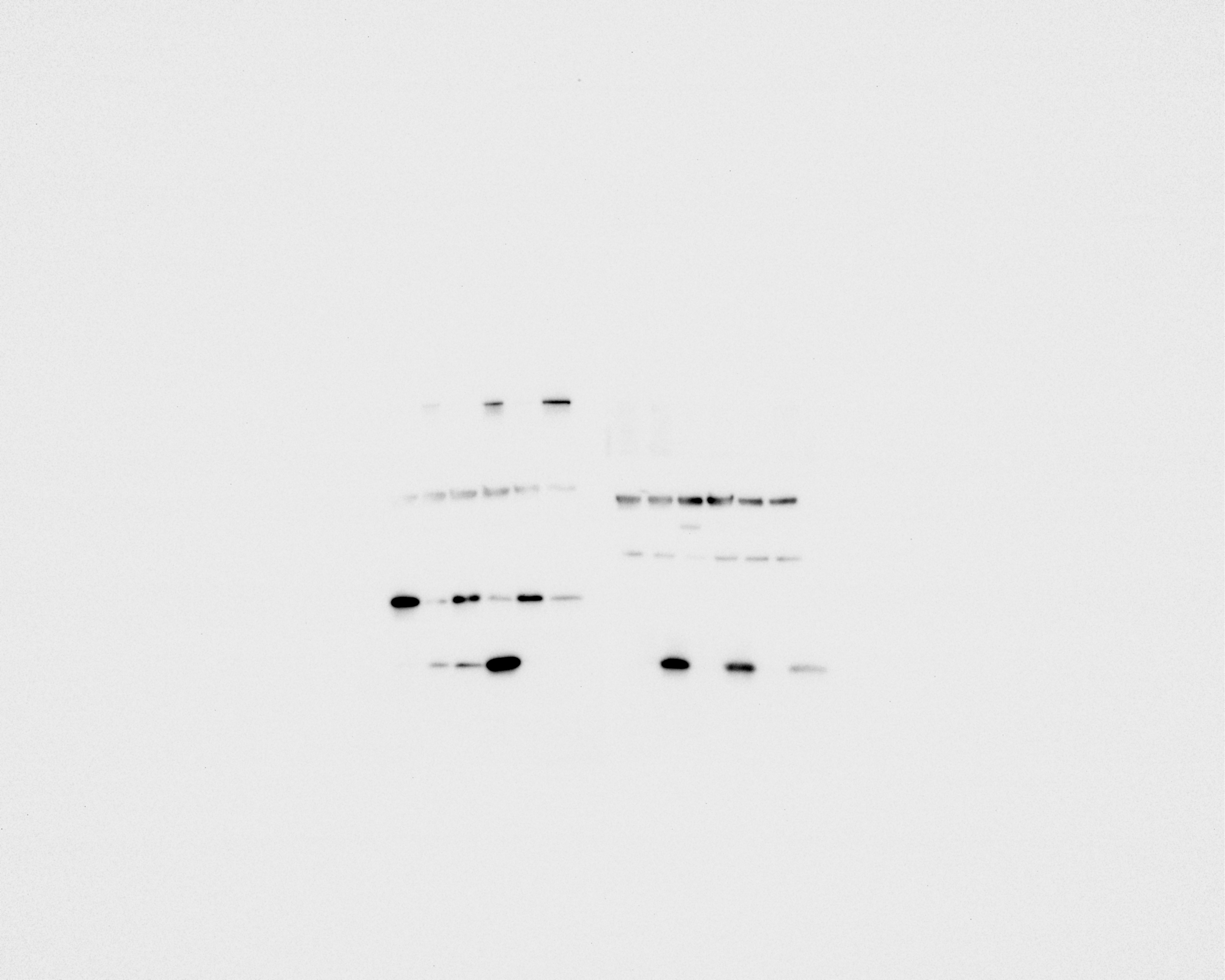

Supplement: Figure 4—source data 1. [file elife-110044-fig4-data1.zip › Figure 4-source data 1/Figure 4c-3.tif]

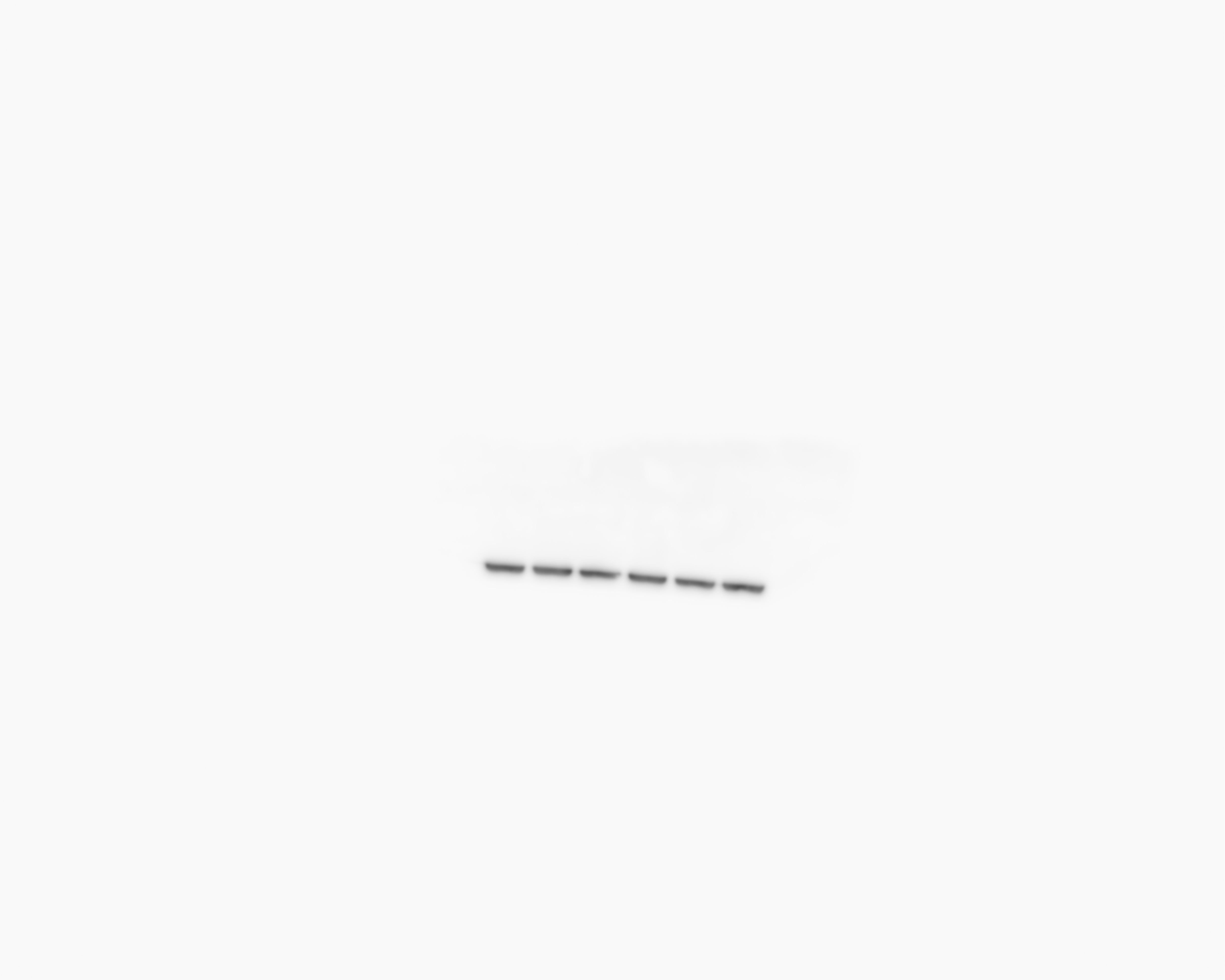

Supplement: Figure 4—source data 1. [file elife-110044-fig4-data1.zip › Figure 4-source data 1/Figure 4c-2.tif]

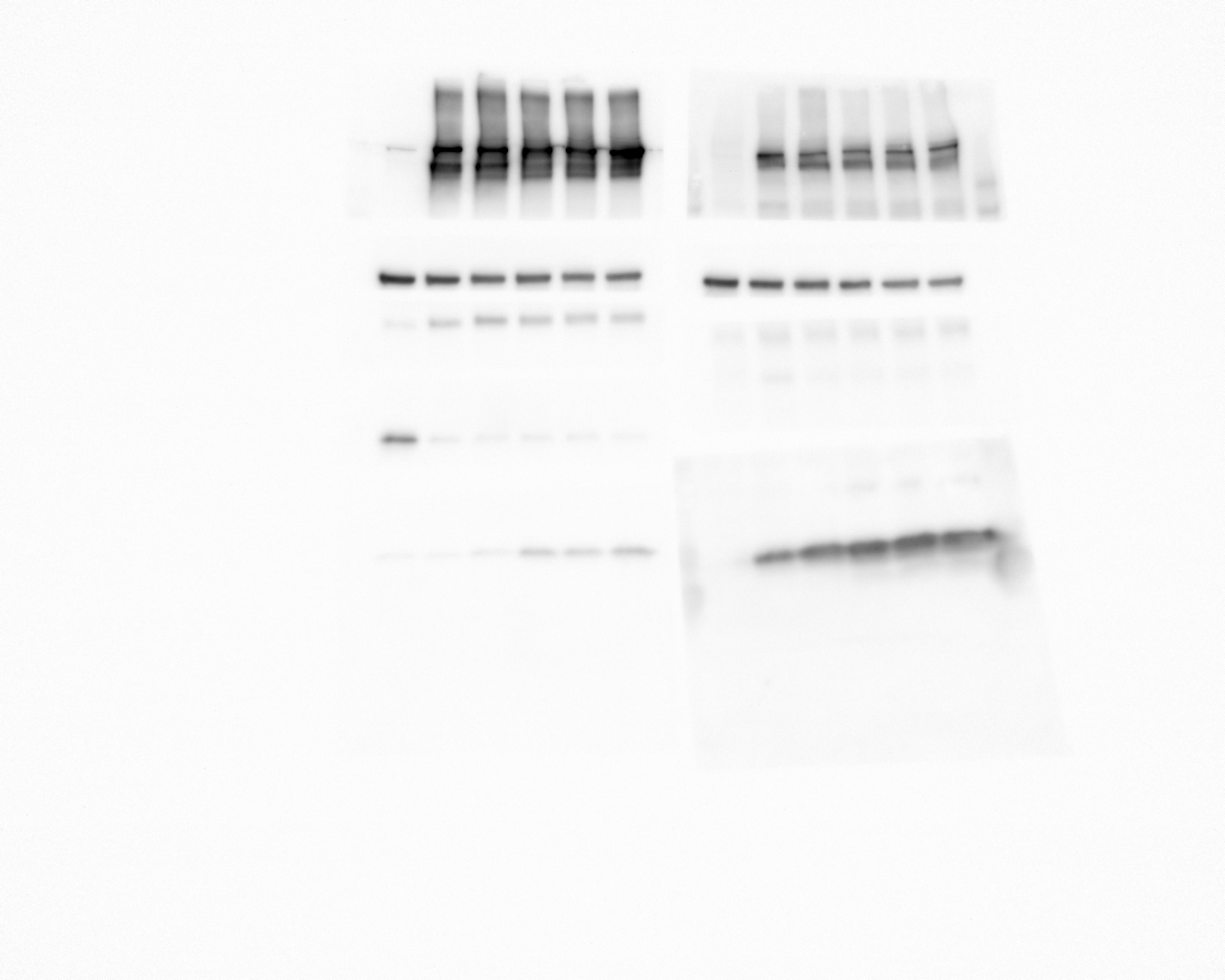

Supplement: Figure 4—source data 1. [file elife-110044-fig4-data1.zip › Figure 4-source data 1/Figure 4a-4.tif]

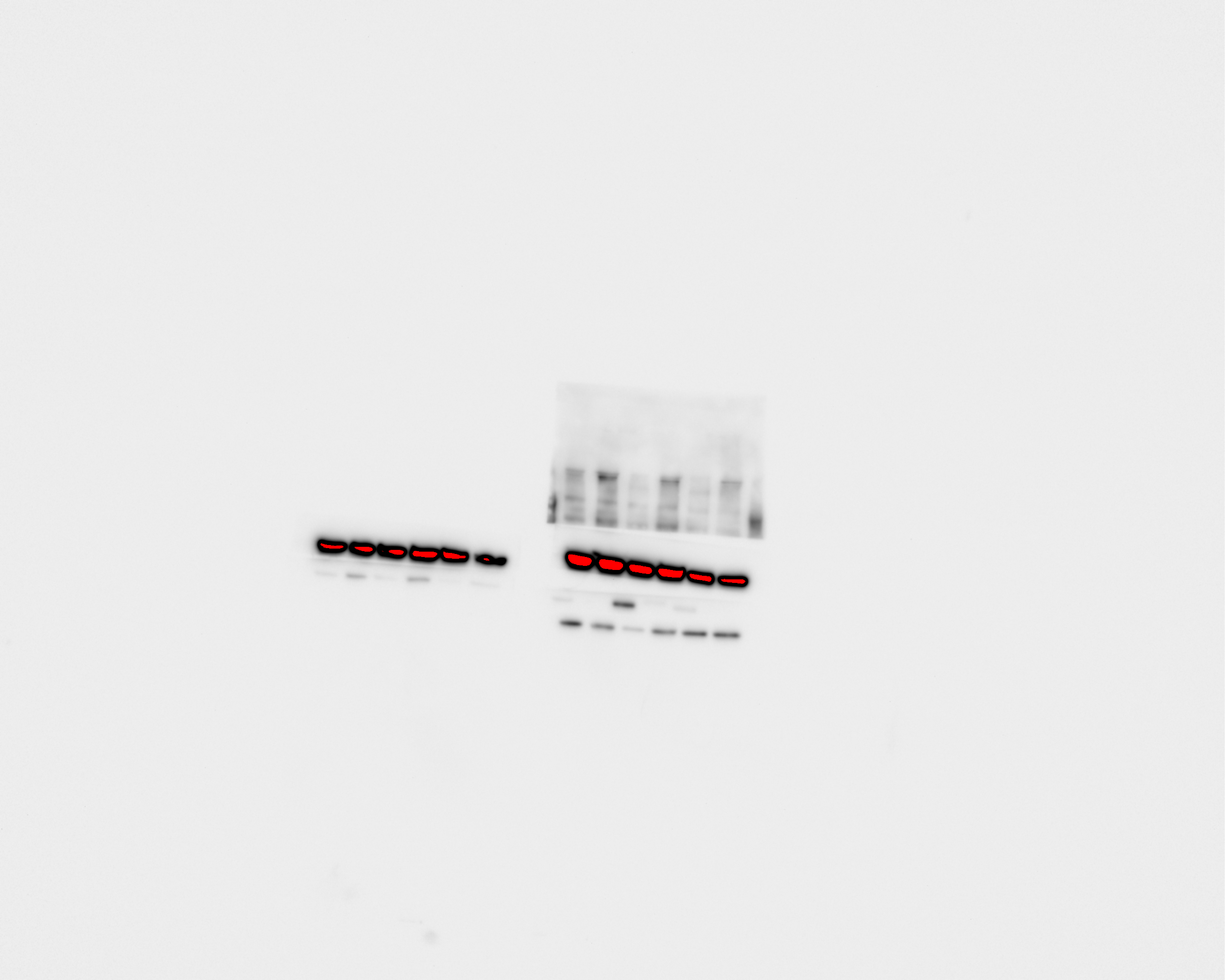

Supplement: Figure 4—source data 1. [file elife-110044-fig4-data1.zip › Figure 4-source data 1/Figure 4c-6.tif]

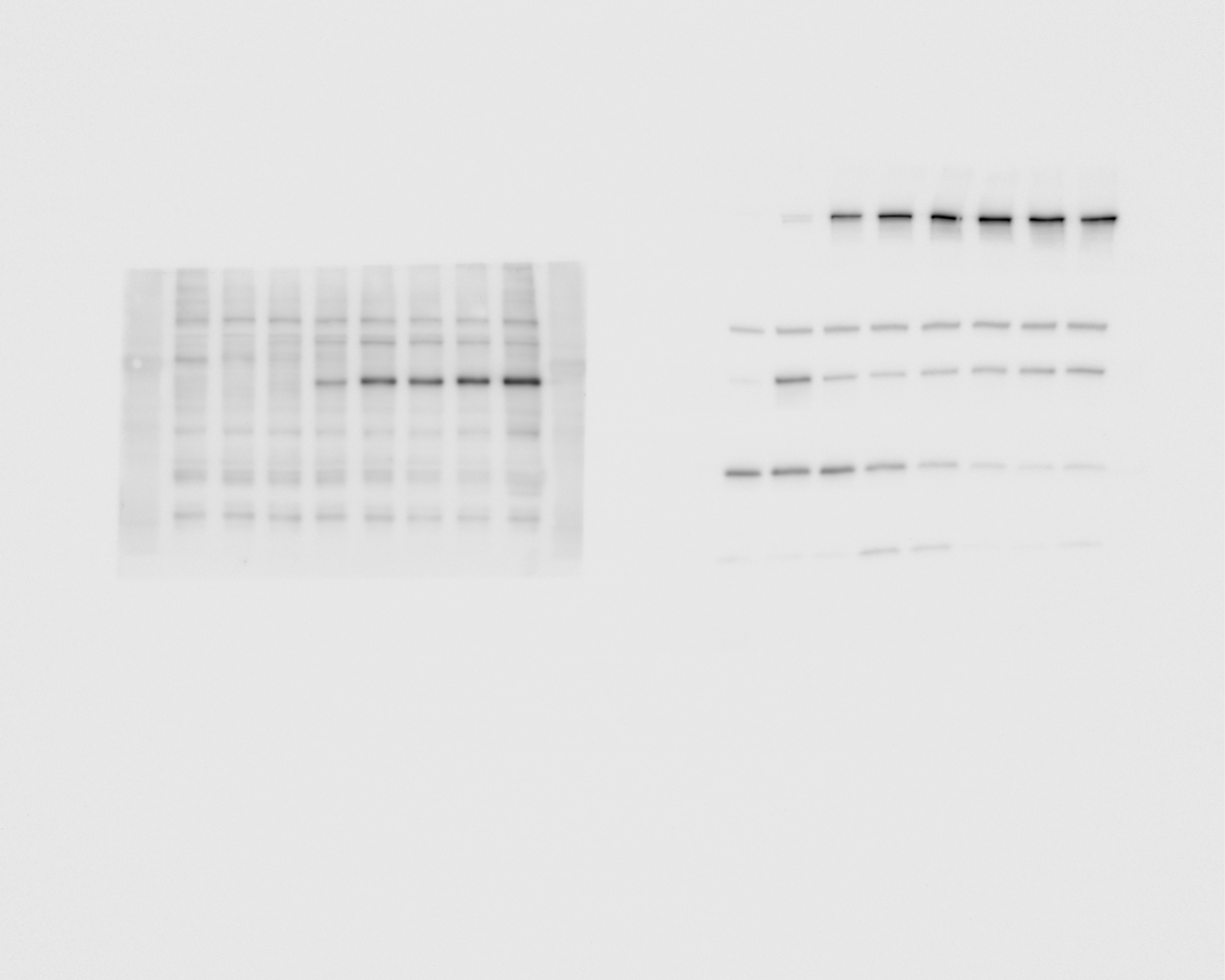

Supplement: Figure 4—source data 1. [file elife-110044-fig4-data1.zip › Figure 4-source data 1/Figure 4e-1.tif]

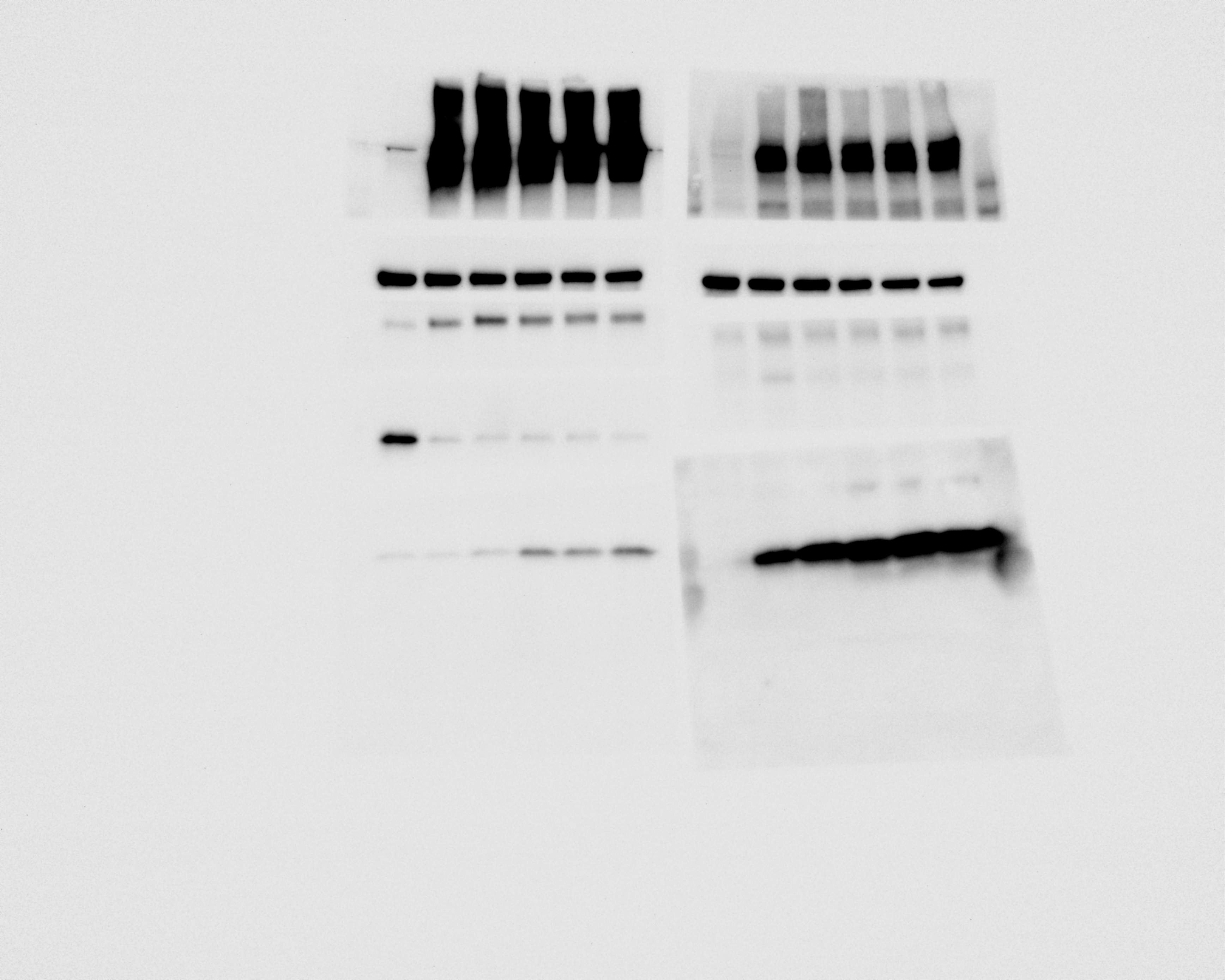

Supplement: Figure 4—source data 1. [file elife-110044-fig4-data1.zip › Figure 4-source data 1/Figure 4a-5.tif]

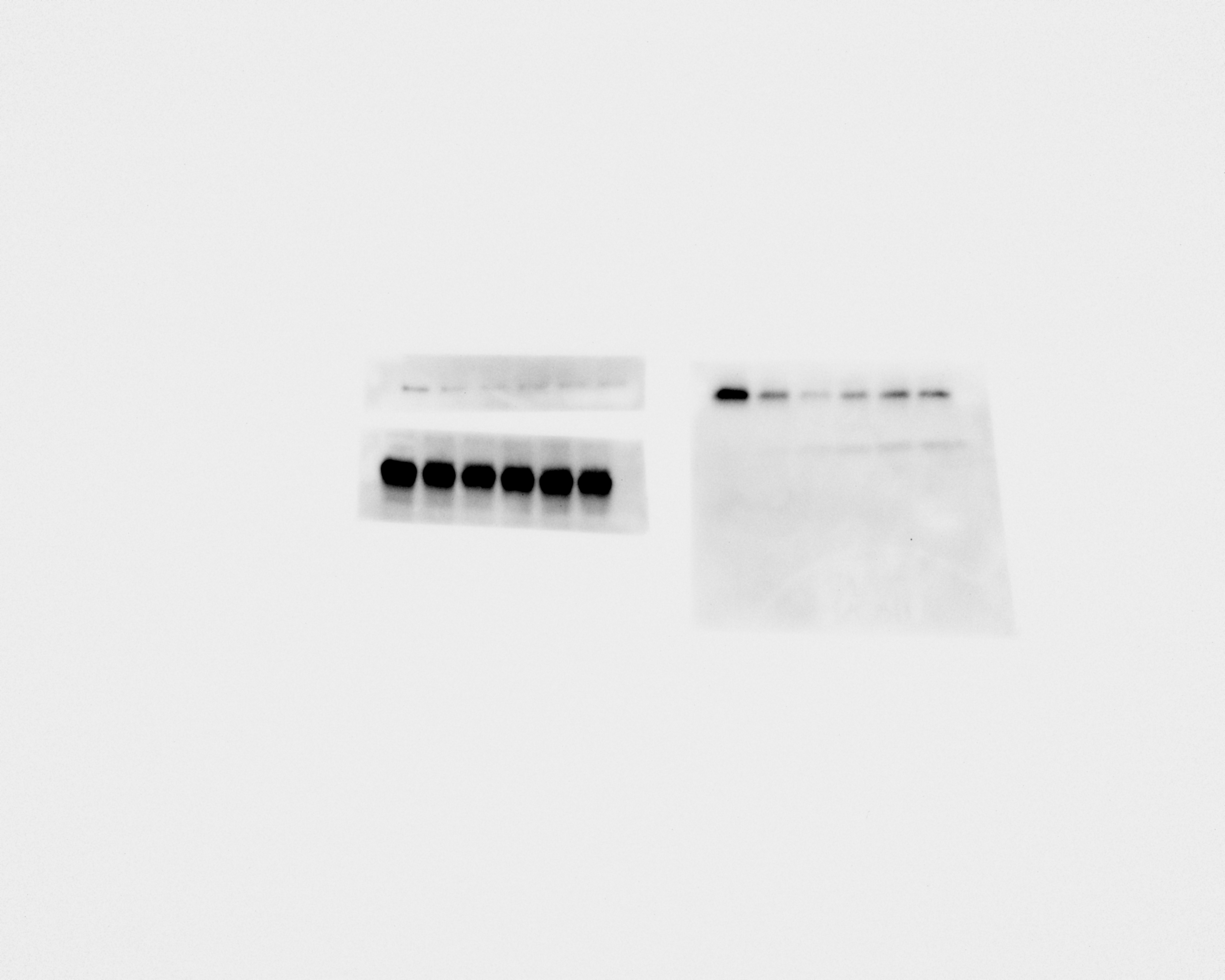

Supplement: Figure 4—source data 1. [file elife-110044-fig4-data1.zip › Figure 4-source data 1/Figure 4a-7.tif]

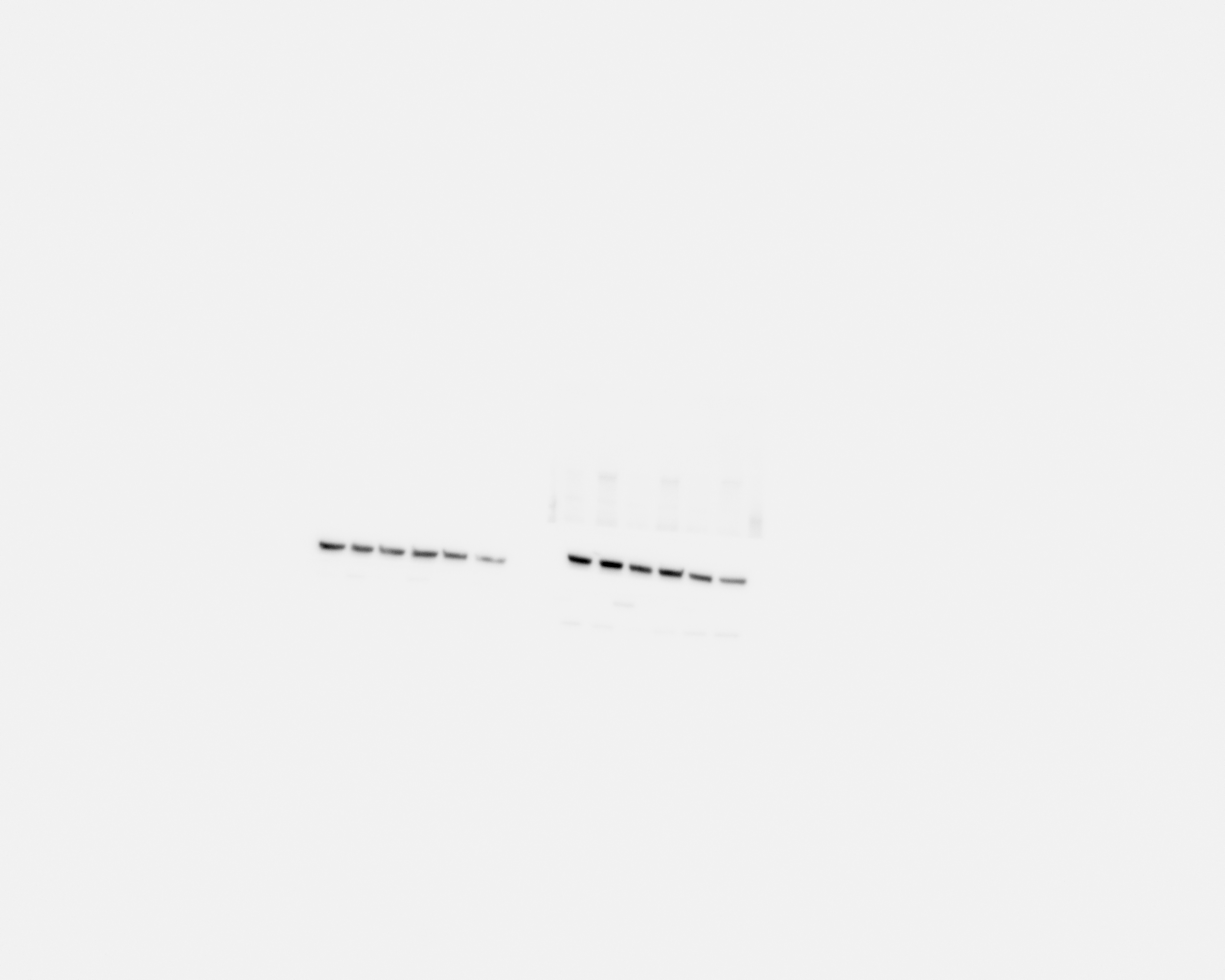

Supplement: Figure 4—source data 1. [file elife-110044-fig4-data1.zip › Figure 4-source data 1/Figure 4c-5.tif]

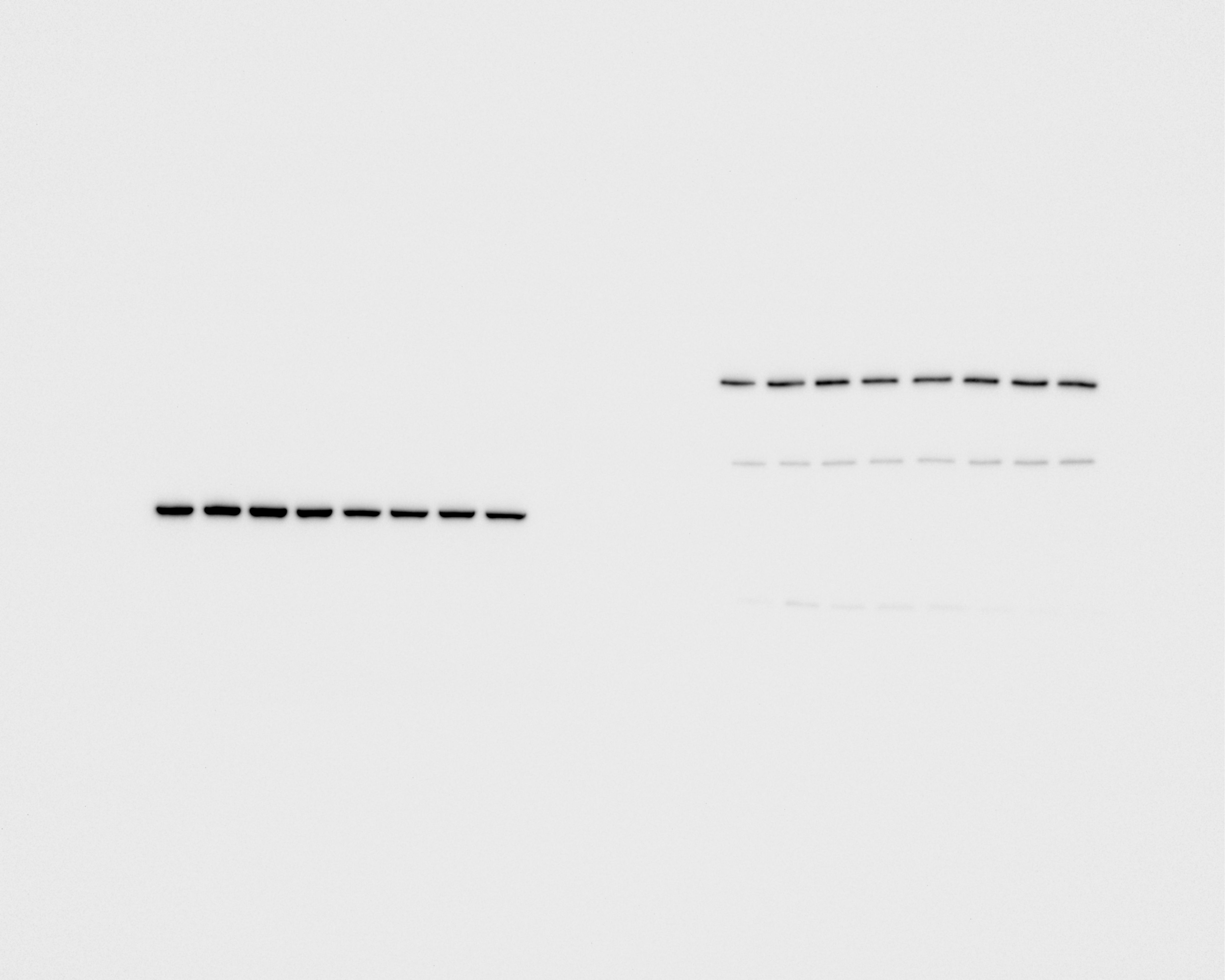

Supplement: Figure 4—source data 1. [file elife-110044-fig4-data1.zip › Figure 4-source data 1/Figure 4e-3.tif]

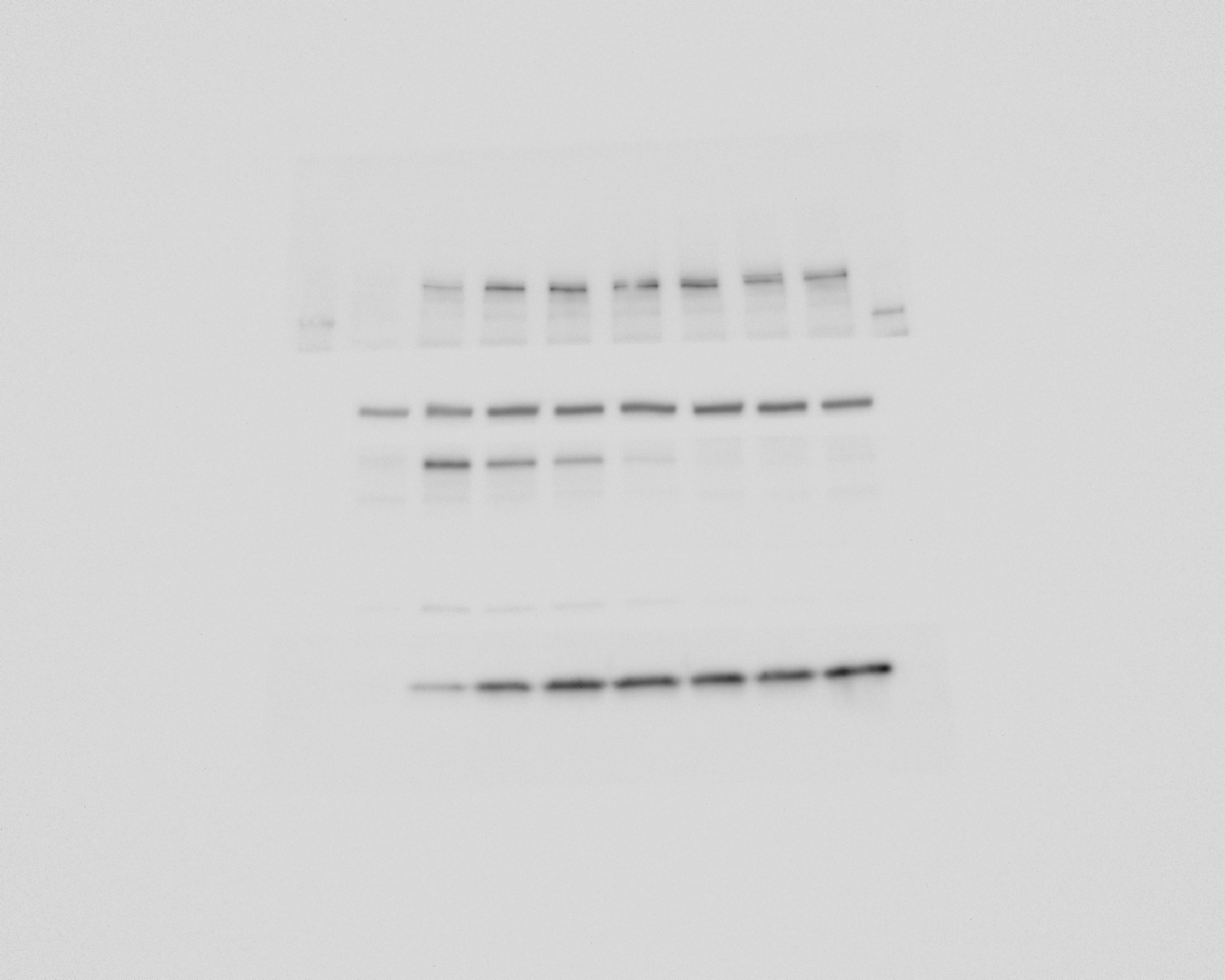

Supplement: Figure 4—source data 1. [file elife-110044-fig4-data1.zip › Figure 4-source data 1/Figure 4e-2.tif]

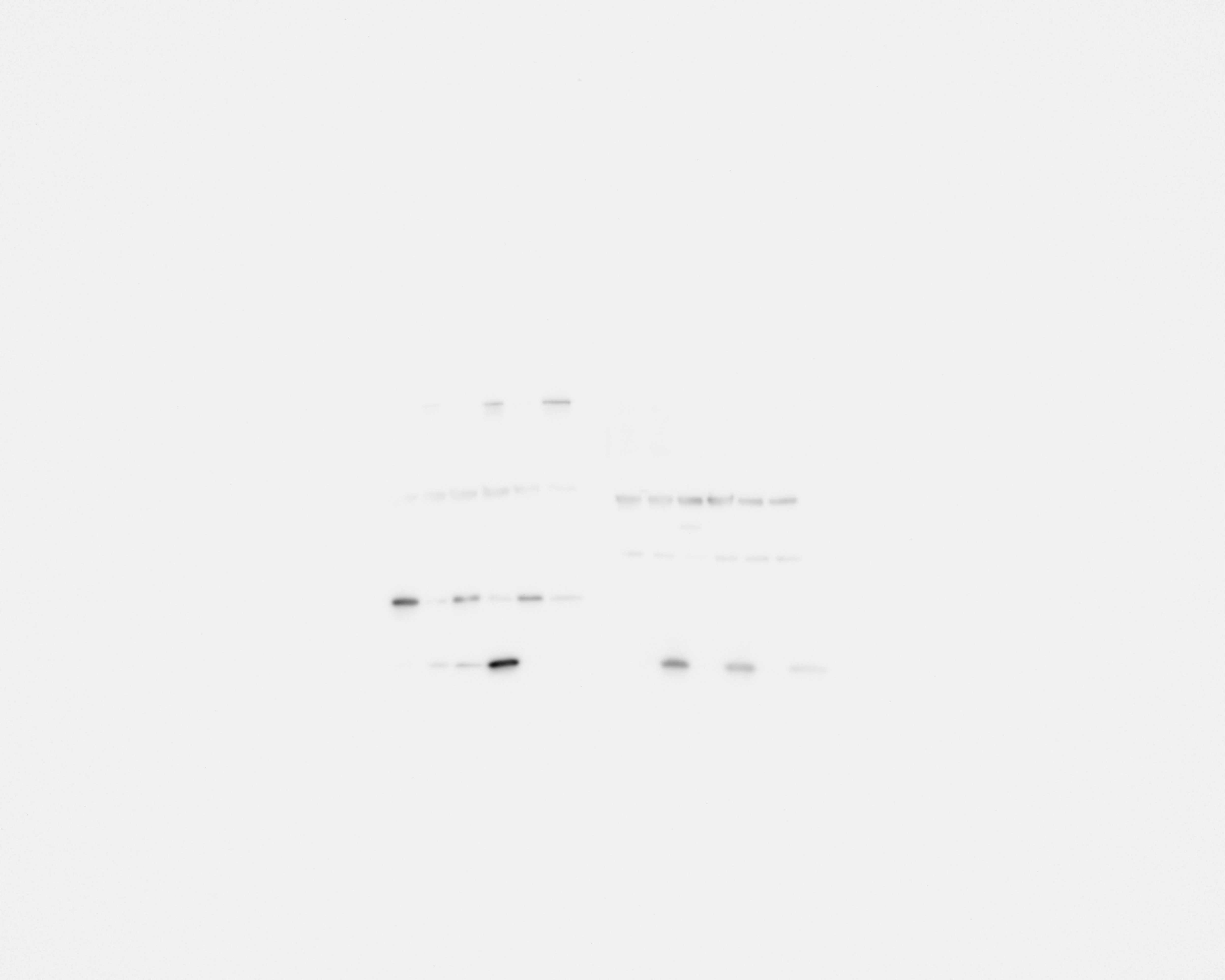

Supplement: Figure 4—source data 1. [file elife-110044-fig4-data1.zip › Figure 4-source data 1/Figure 4c-4.tif]

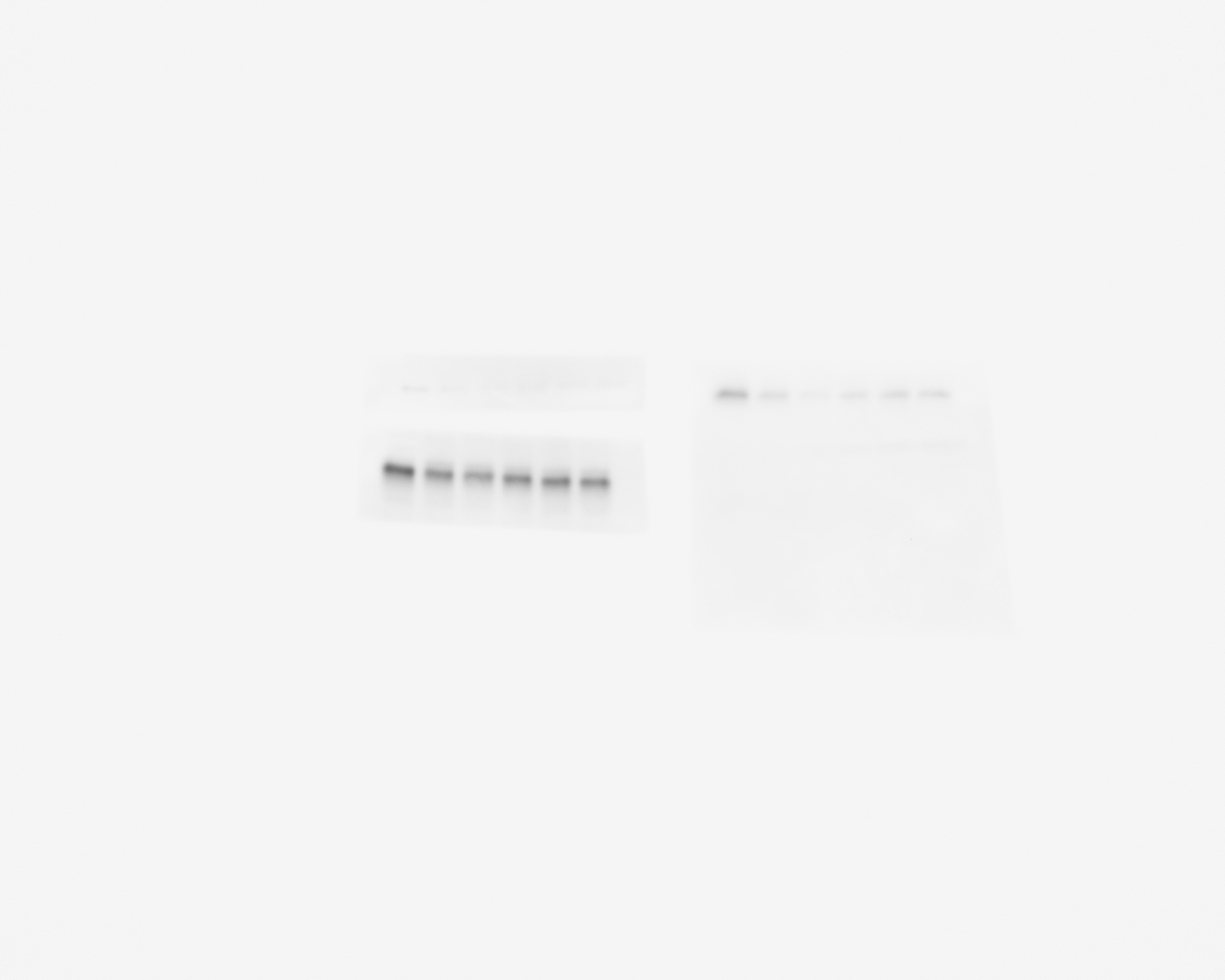

Supplement: Figure 4—source data 1. [file elife-110044-fig4-data1.zip › Figure 4-source data 1/Figure 4a-6.tif]

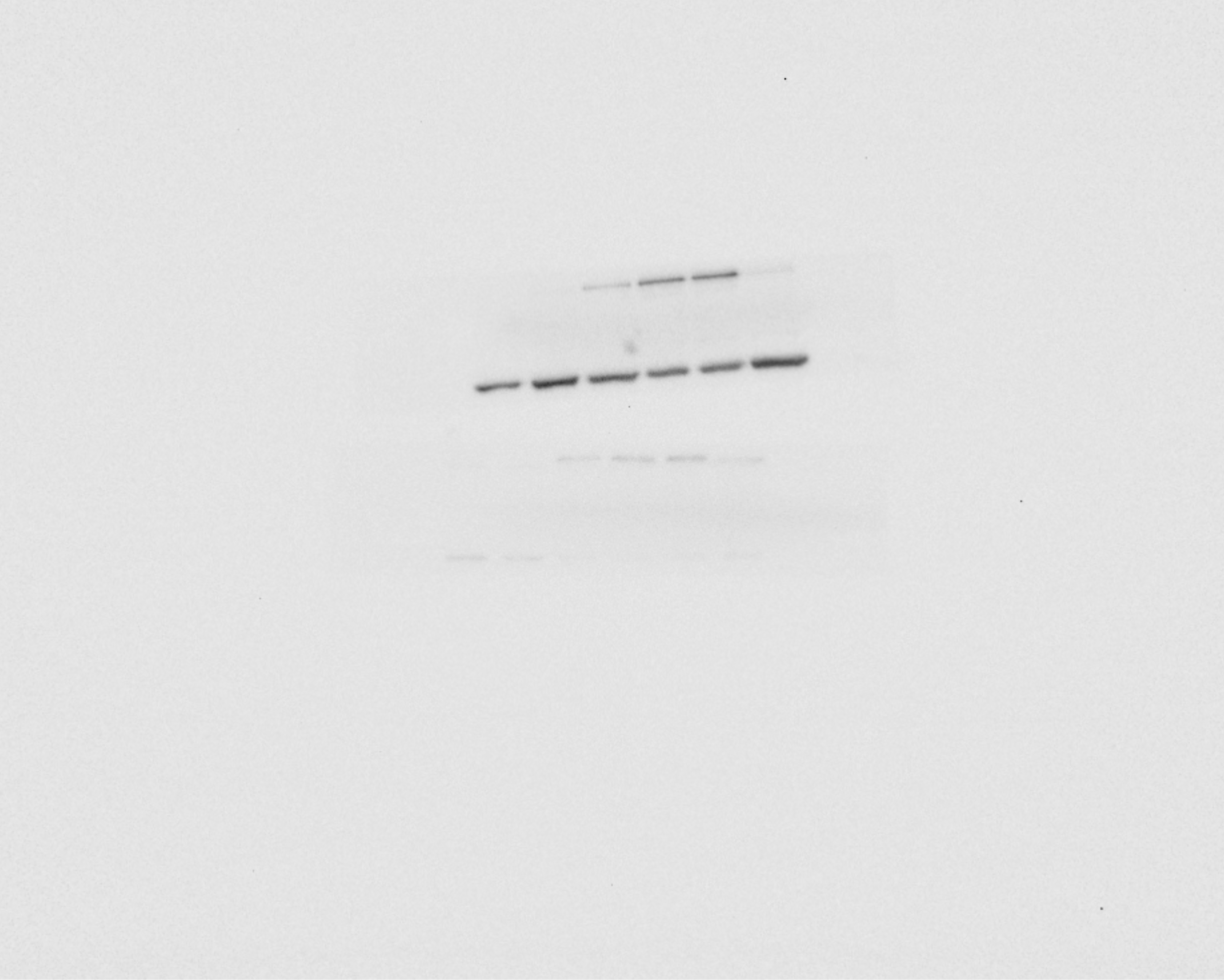

Supplement: Figure 4—source data 1. [file elife-110044-fig4-data1.zip › Figure 4-source data 1/Figure 4d-5.tif]

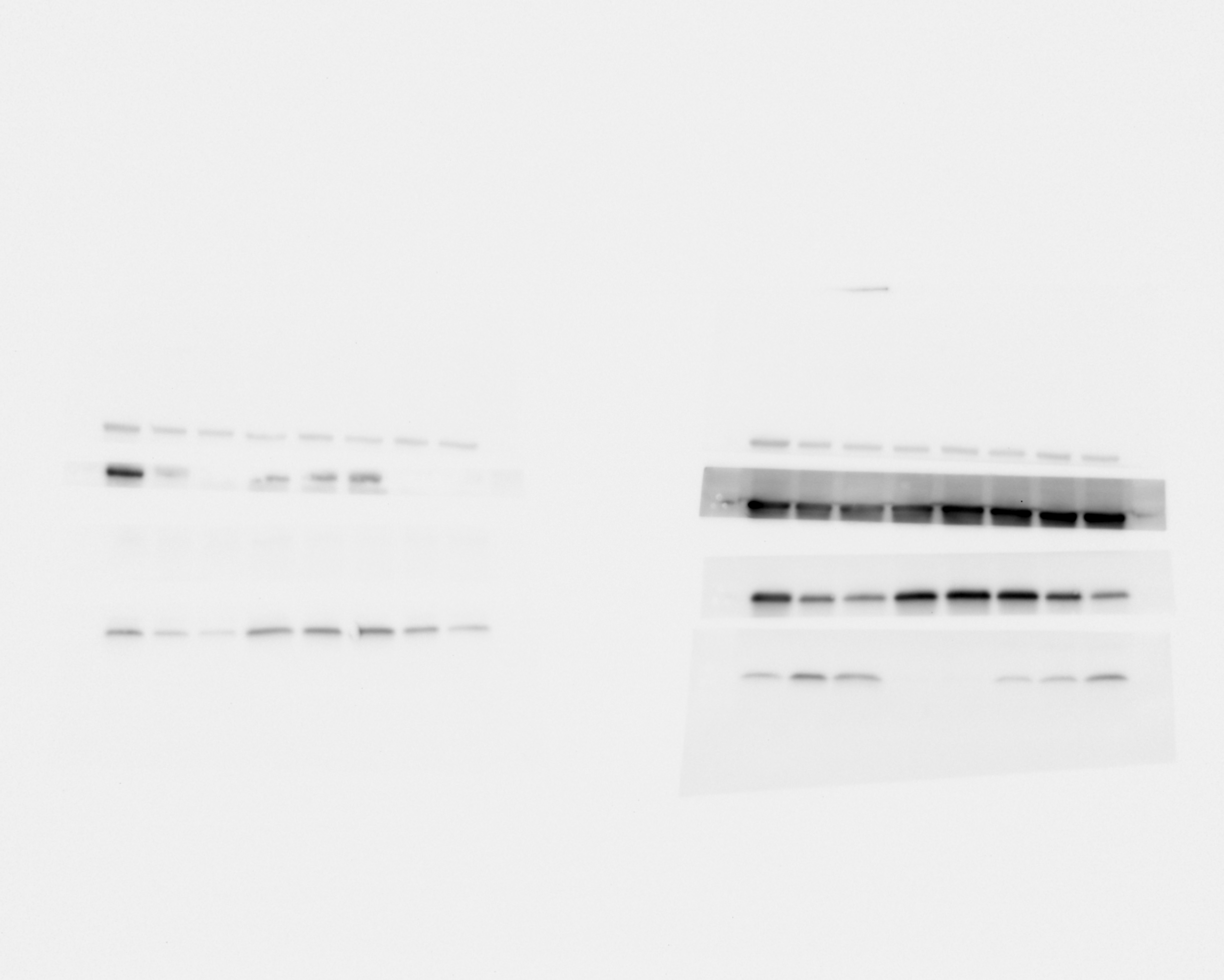

Supplement: Figure 4—source data 1. [file elife-110044-fig4-data1.zip › Figure 4-source data 1/Figure 4b-3.tif]

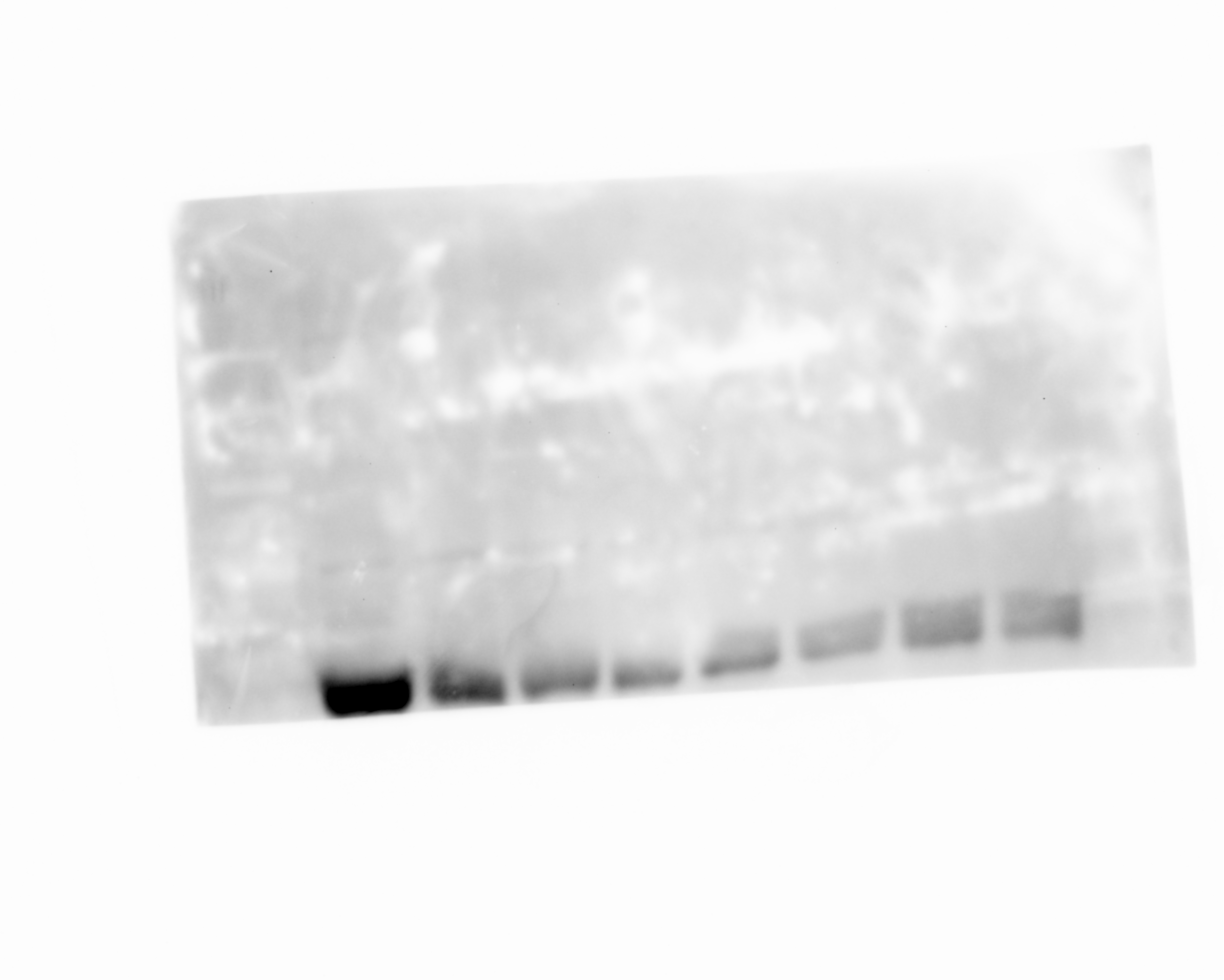

Supplement: Figure 4—source data 1. [file elife-110044-fig4-data1.zip › Figure 4-source data 1/Figure 4b-2.tif]

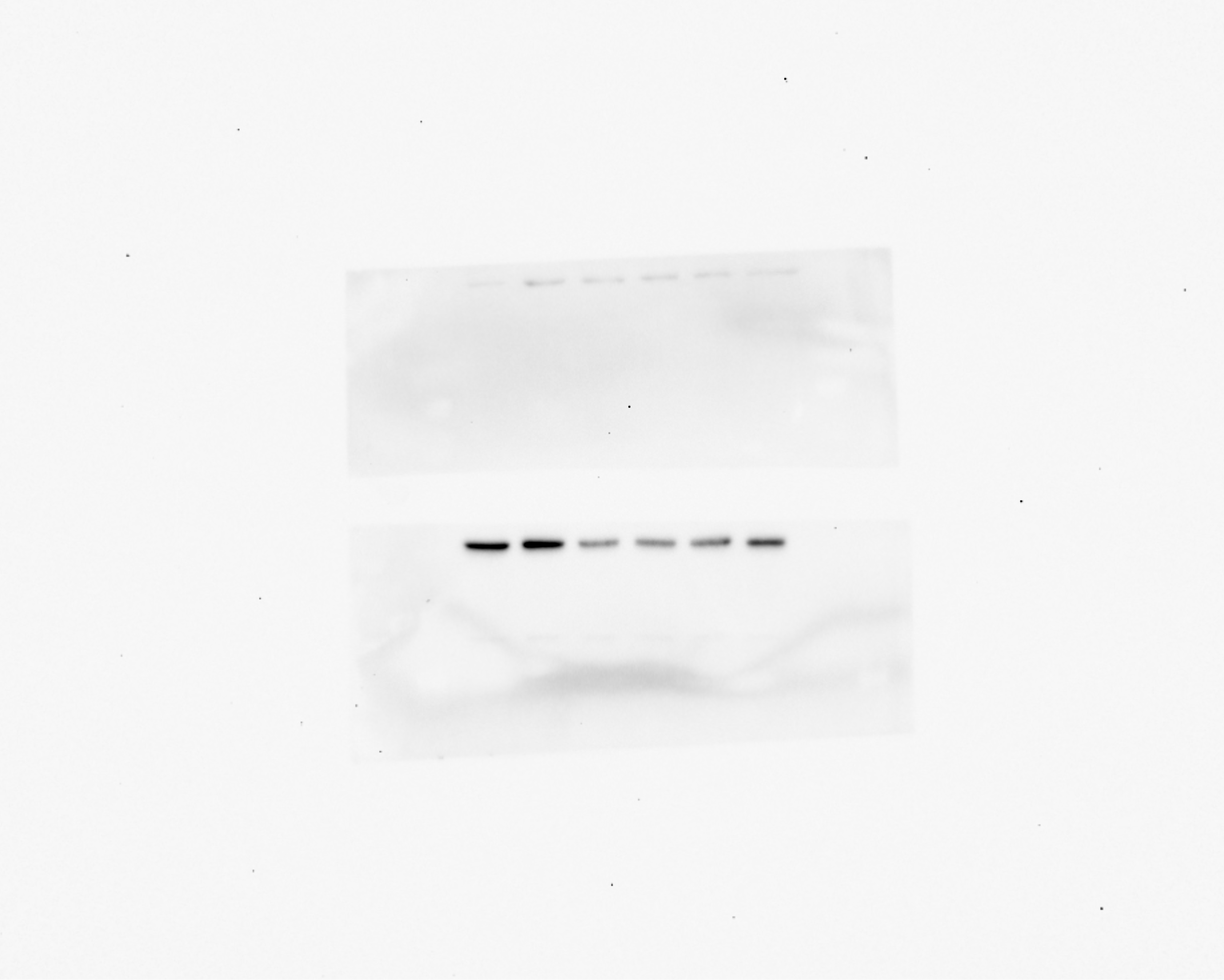

Supplement: Figure 4—source data 1. [file elife-110044-fig4-data1.zip › Figure 4-source data 1/Figure 4d-4.tif]

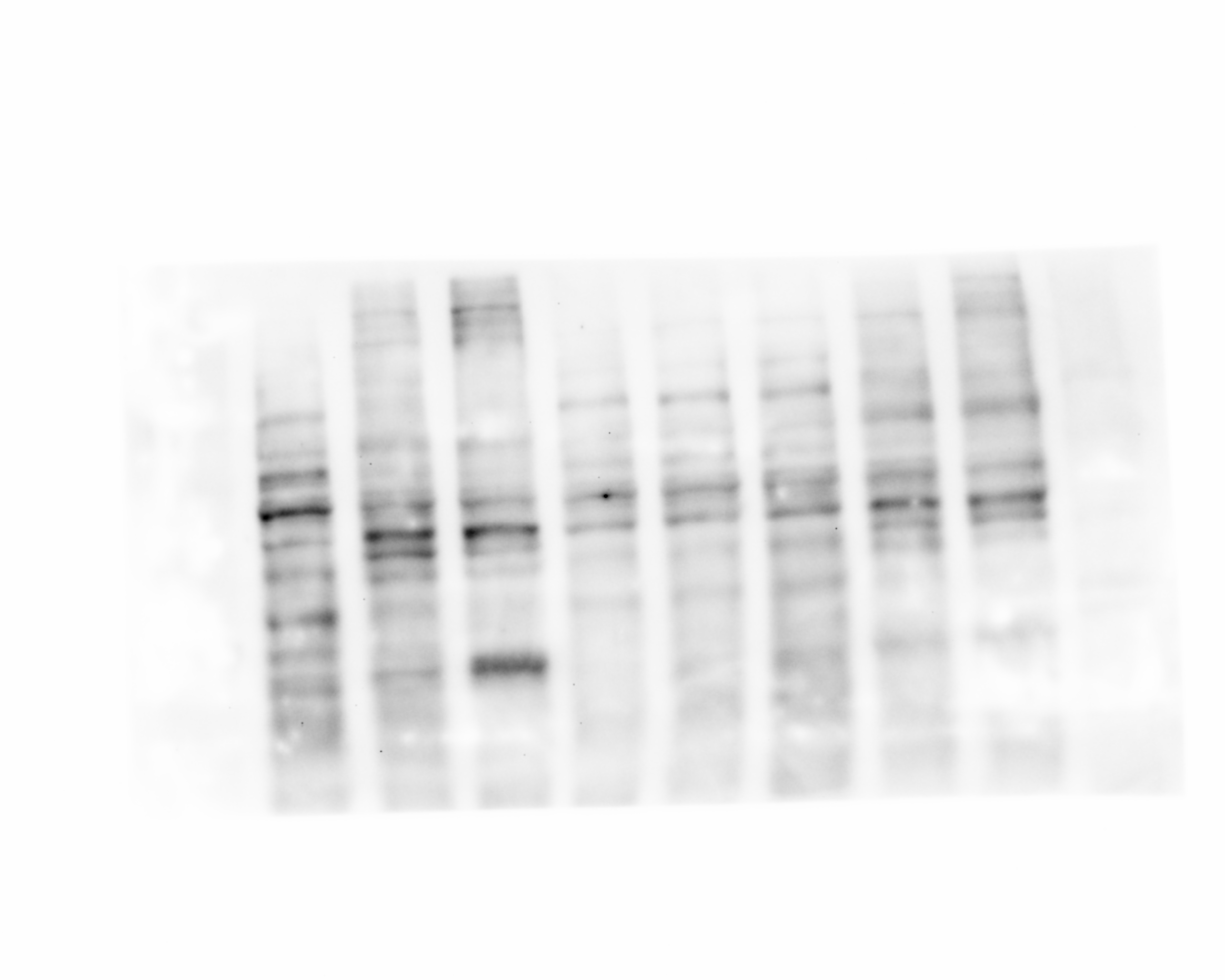

Supplement: Figure 4—source data 1. [file elife-110044-fig4-data1.zip › Figure 4-source data 1/Figure 4b-1.tif]

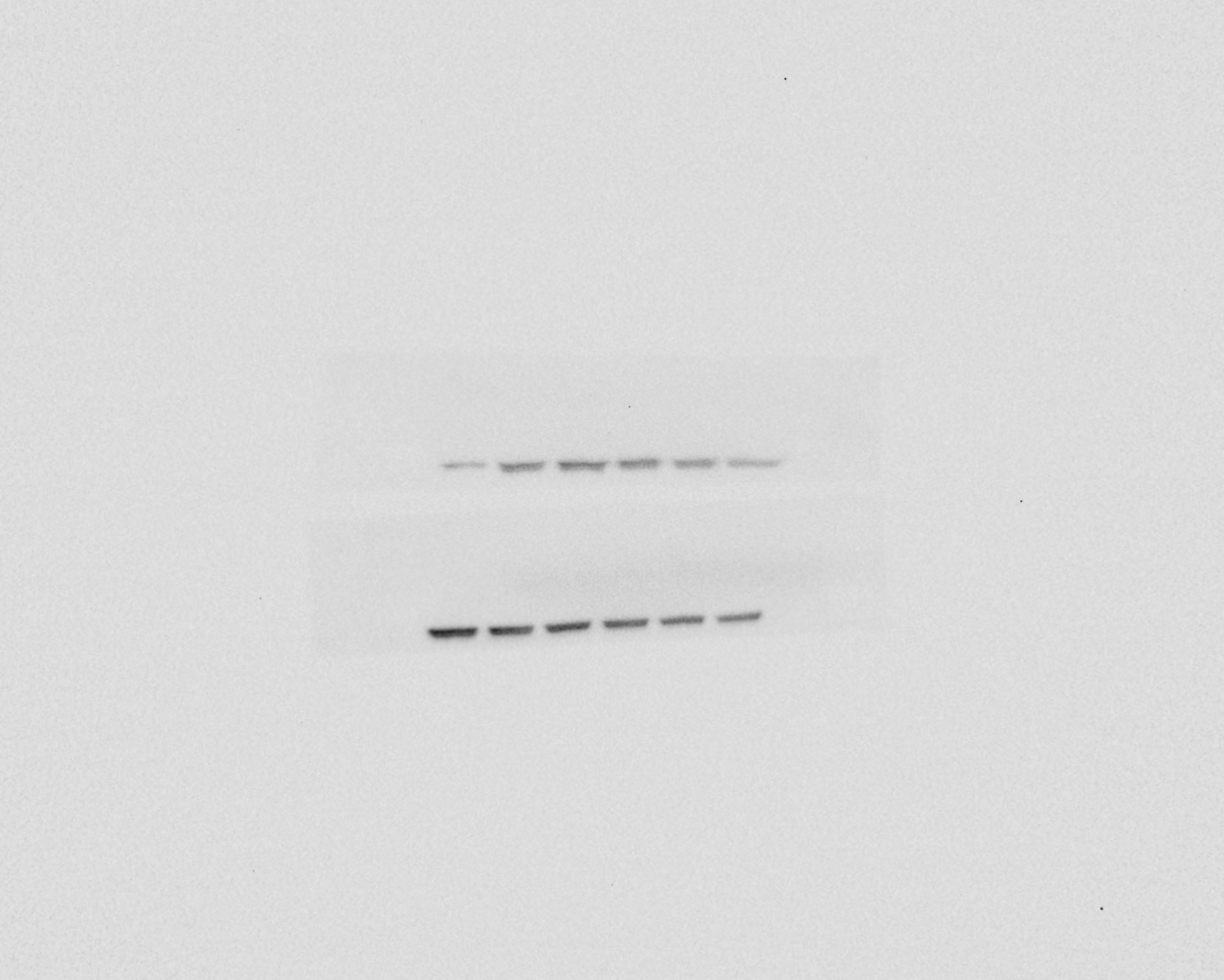

Supplement: Figure 4—source data 1. [file elife-110044-fig4-data1.zip › Figure 4-source data 1/Figure 4d-3.tif]

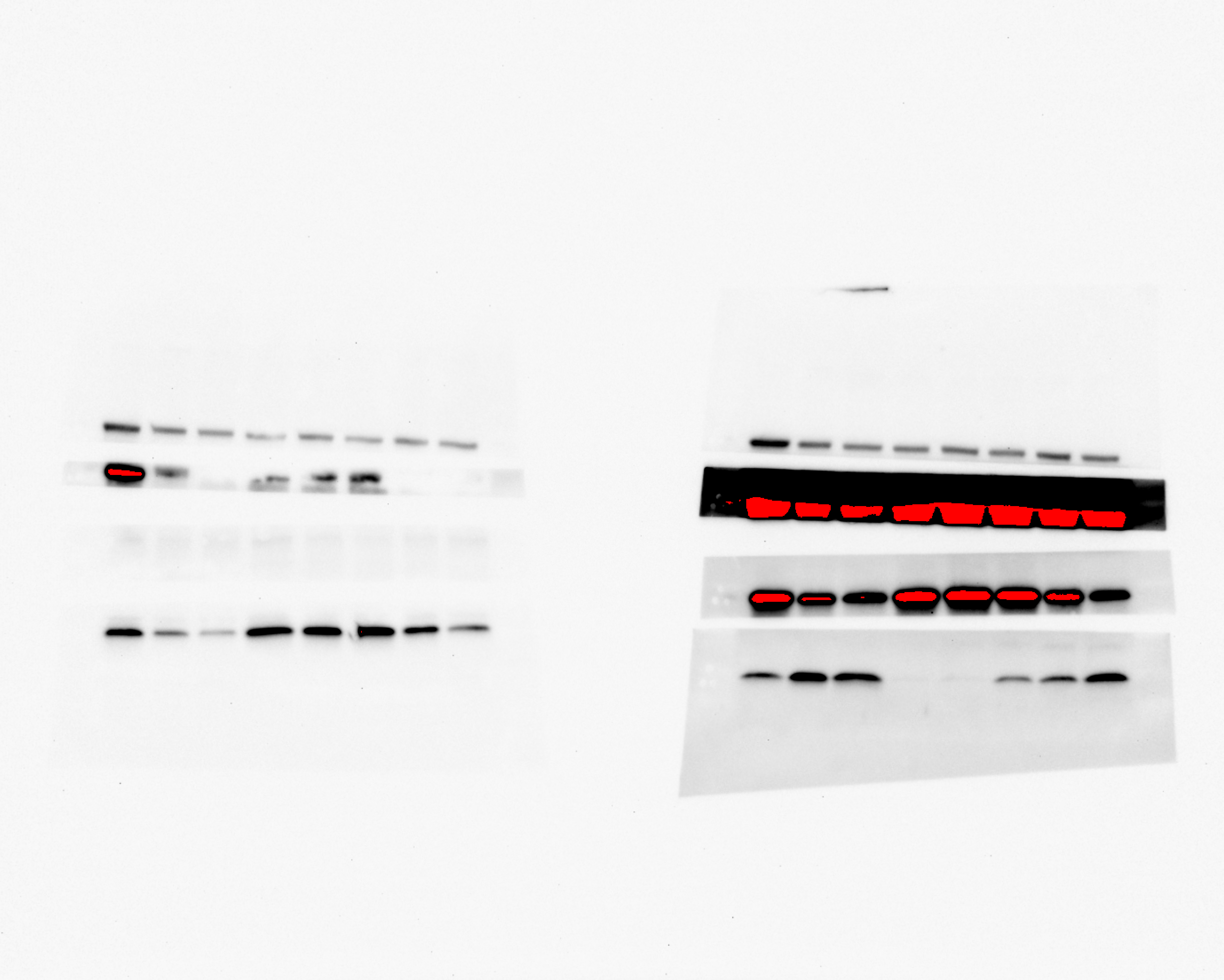

Supplement: Figure 4—source data 1. [file elife-110044-fig4-data1.zip › Figure 4-source data 1/Figure 4b-4.tif]

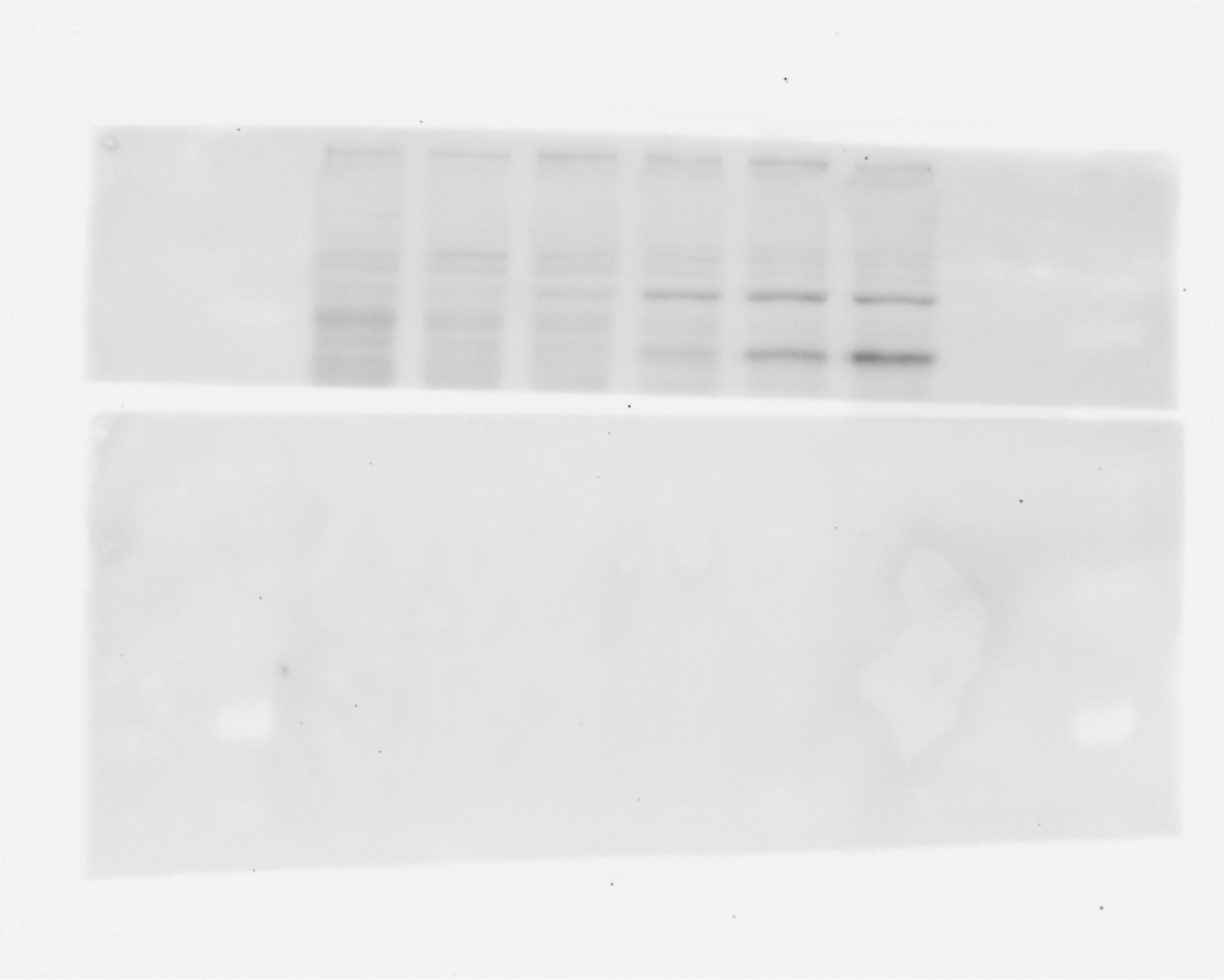

Supplement: Figure 4—source data 1. [file elife-110044-fig4-data1.zip › Figure 4-source data 1/Figure 4d-2.tif]

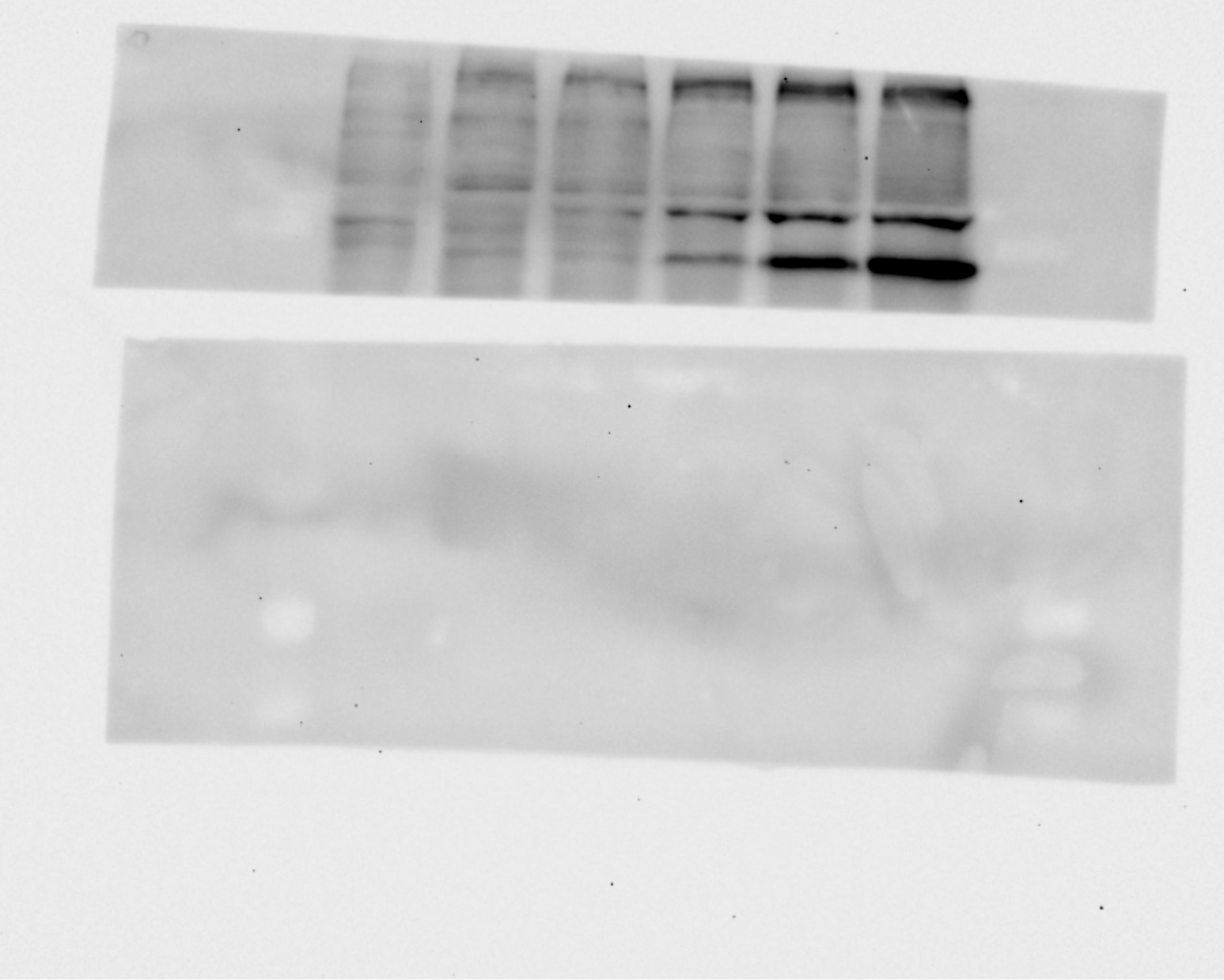

Supplement: Figure 4—source data 1. [file elife-110044-fig4-data1.zip › Figure 4-source data 1/Figure 4d-1.tif]

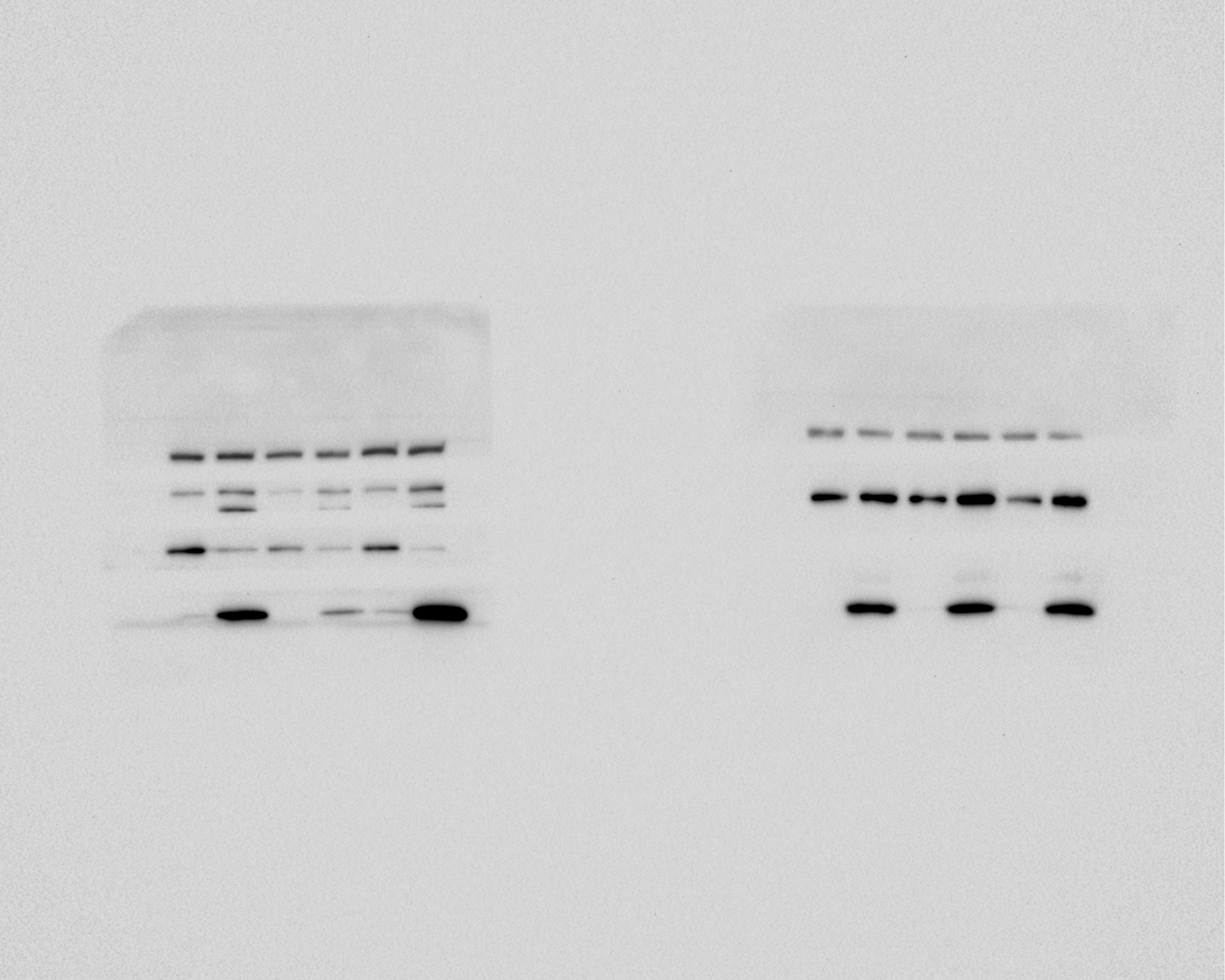

Supplement: Figure 4—figure supplement 1—source data 1. [file elife-110044-fig4-figsupp1-data1.zip › Figure 4 Supplement 1-source data 1/Figure 4 Supplement 1-a4.tif]

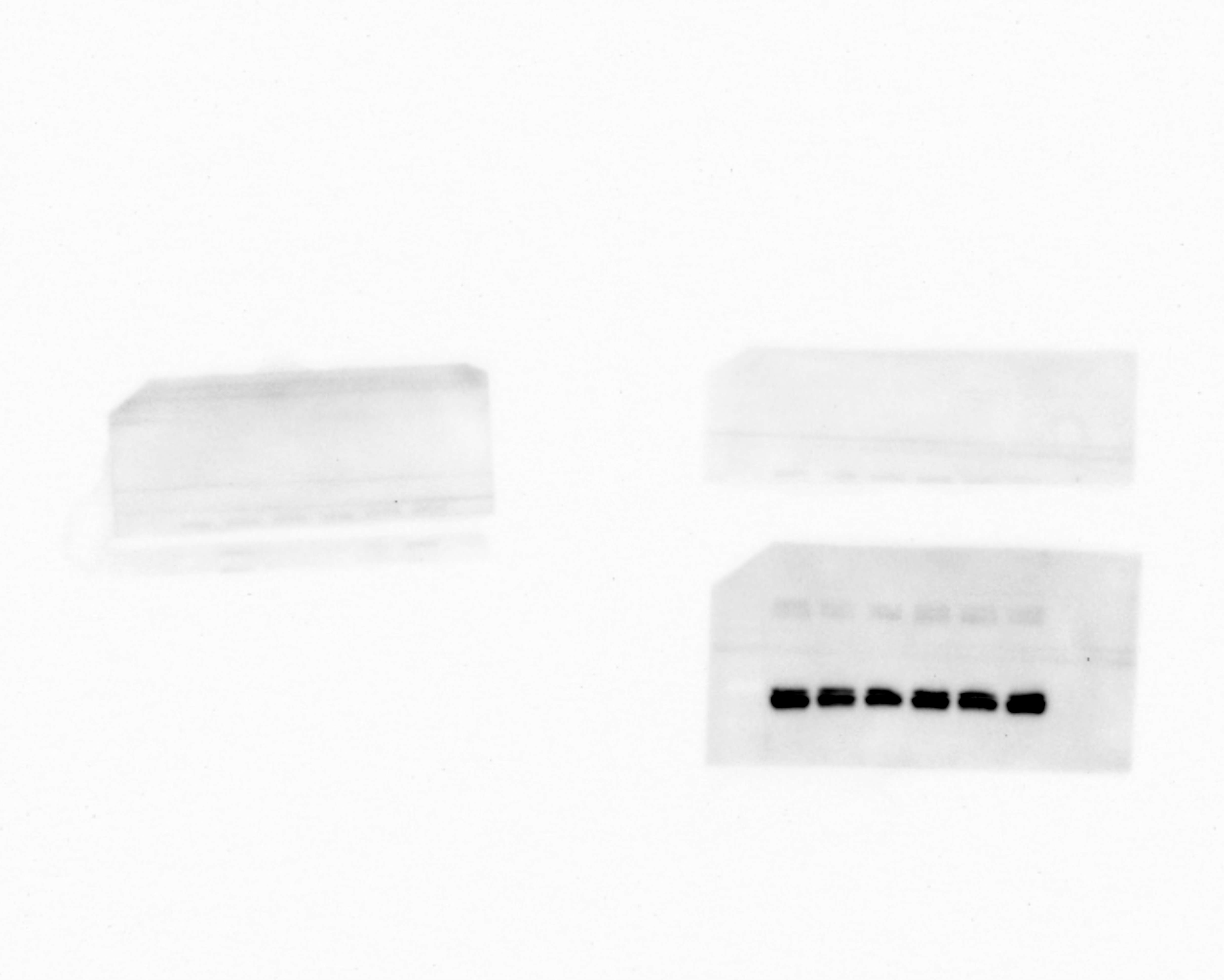

Supplement: Figure 4—figure supplement 1—source data 1. [file elife-110044-fig4-figsupp1-data1.zip › Figure 4 Supplement 1-source data 1/Figure 4 Supplement 1-a3.tif]

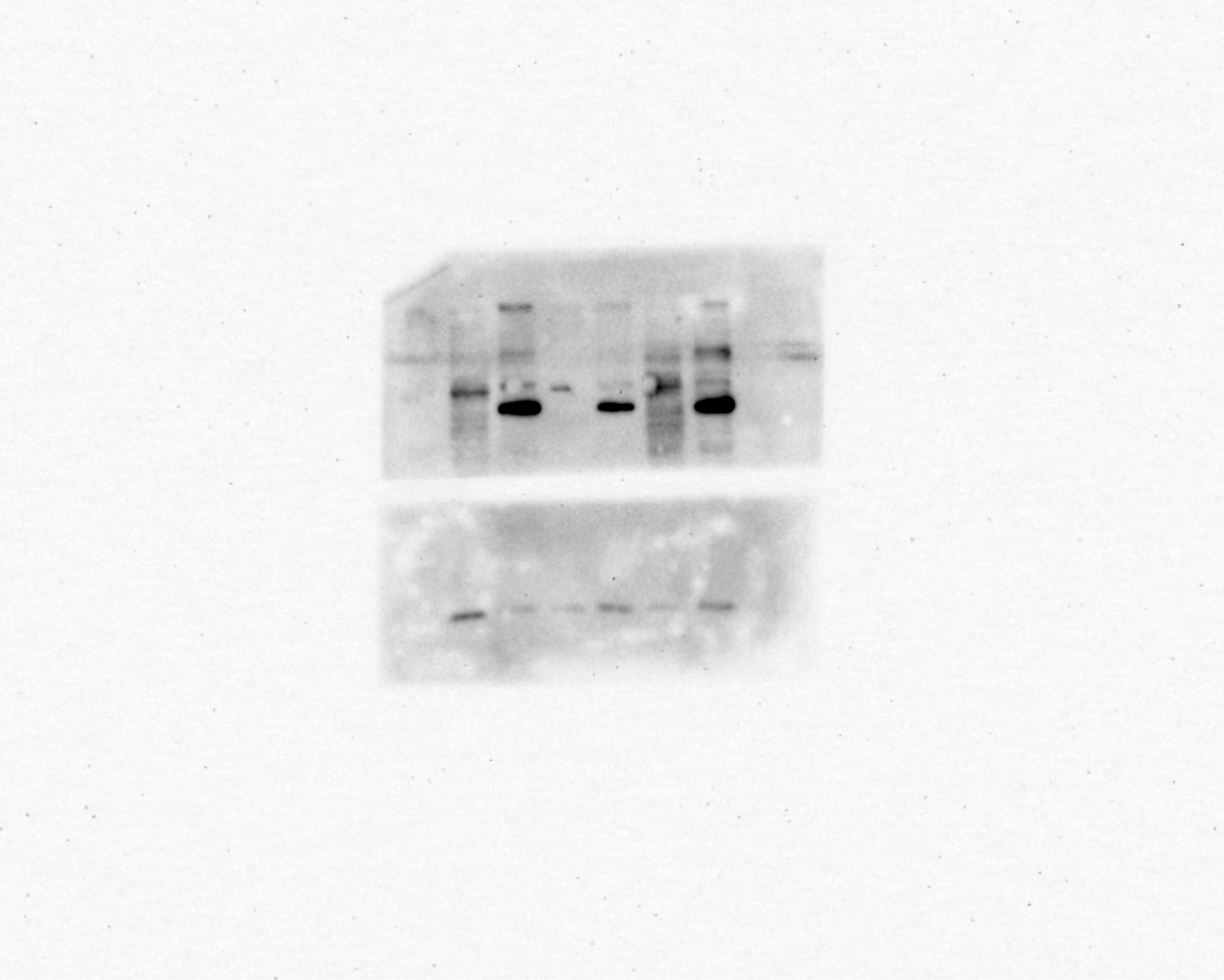

Supplement: Figure 4—figure supplement 1—source data 1. [file elife-110044-fig4-figsupp1-data1.zip › Figure 4 Supplement 1-source data 1/Figure 4 Supplement 1-a2.tif]

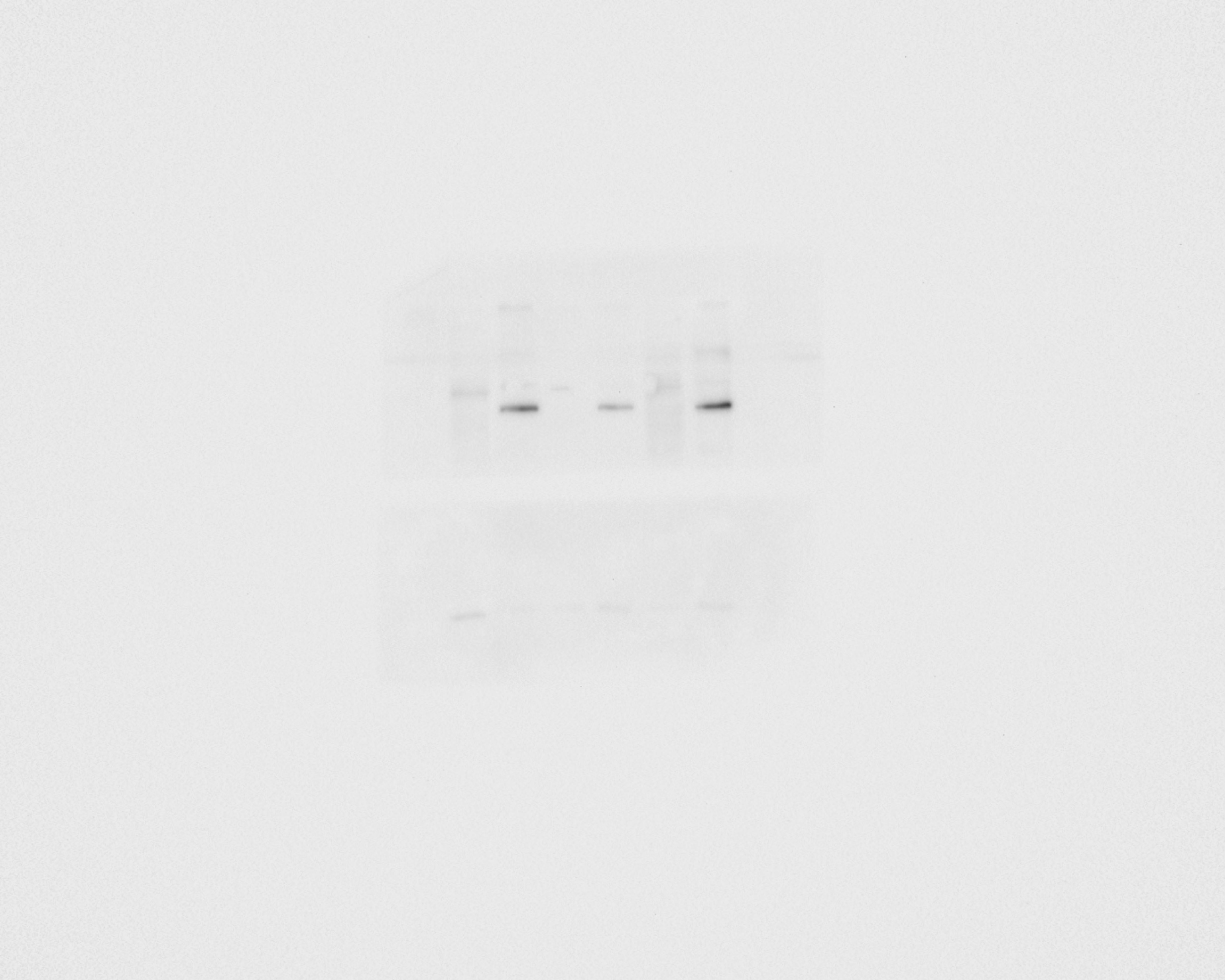

Supplement: Figure 4—figure supplement 1—source data 1. [file elife-110044-fig4-figsupp1-data1.zip › Figure 4 Supplement 1-source data 1/Figure 4 Supplement 1-a1.tif]

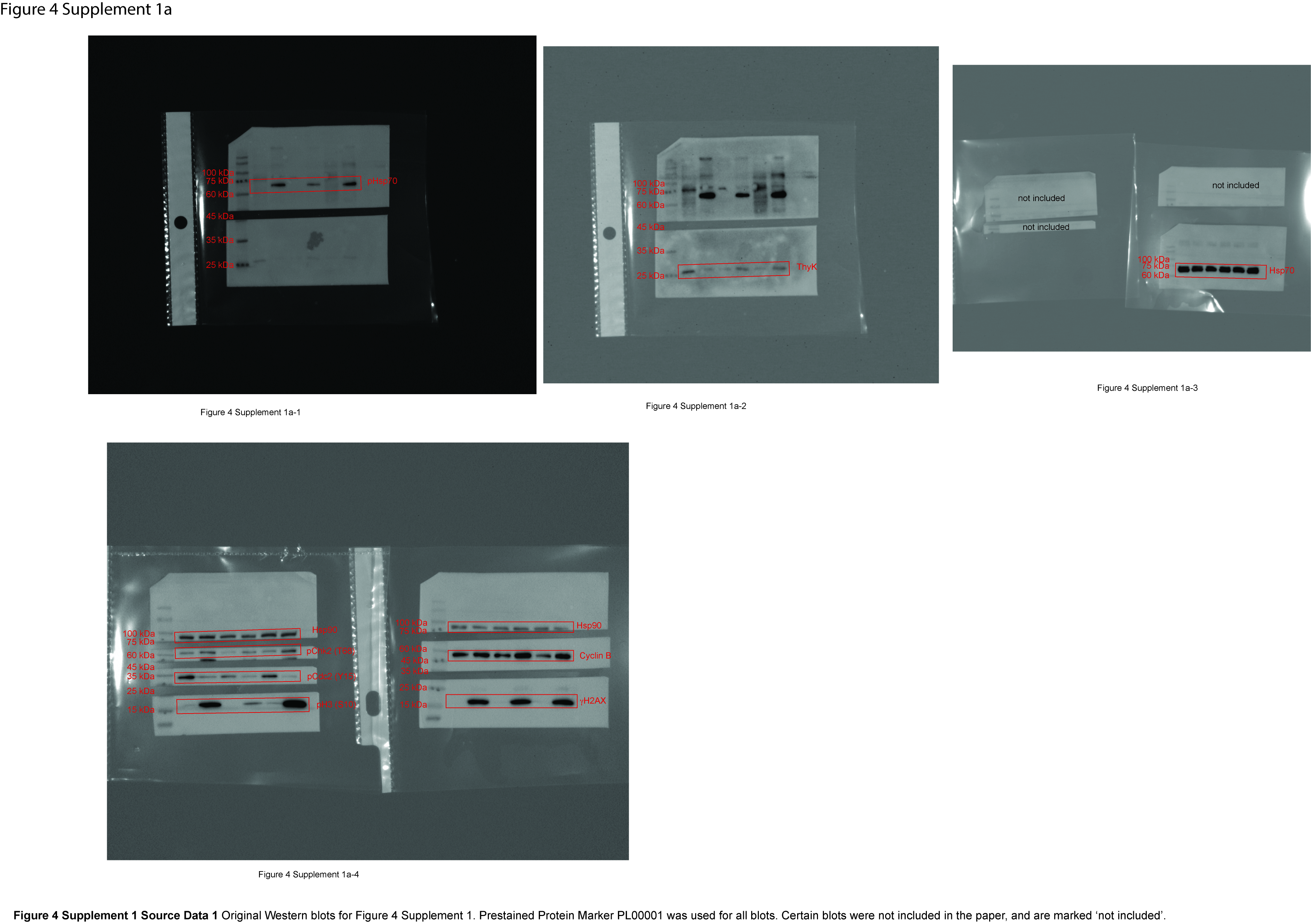

Supplement: Figure 4—figure supplement 1—source data 2. [file elife-110044-fig4-figsupp1-data2.zip › Figure 4 Supplement 1-source data 2/Figure 4 Supplement 1 - Source Data 2.tif]

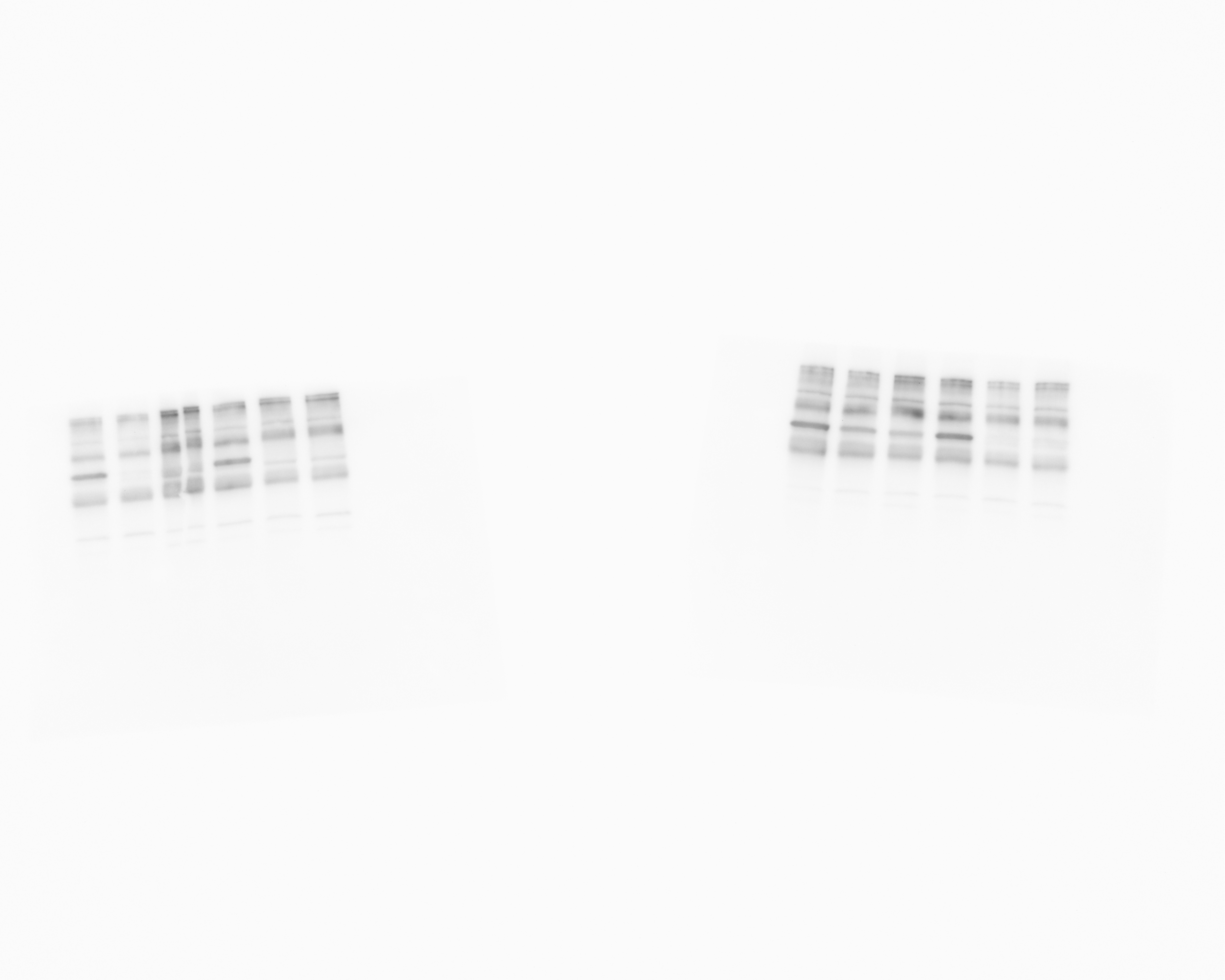

Supplement: Figure 5—source data 1. [file elife-110044-fig5-data1.zip › Figure 5-source data 1/Figure 5a-1.tif]

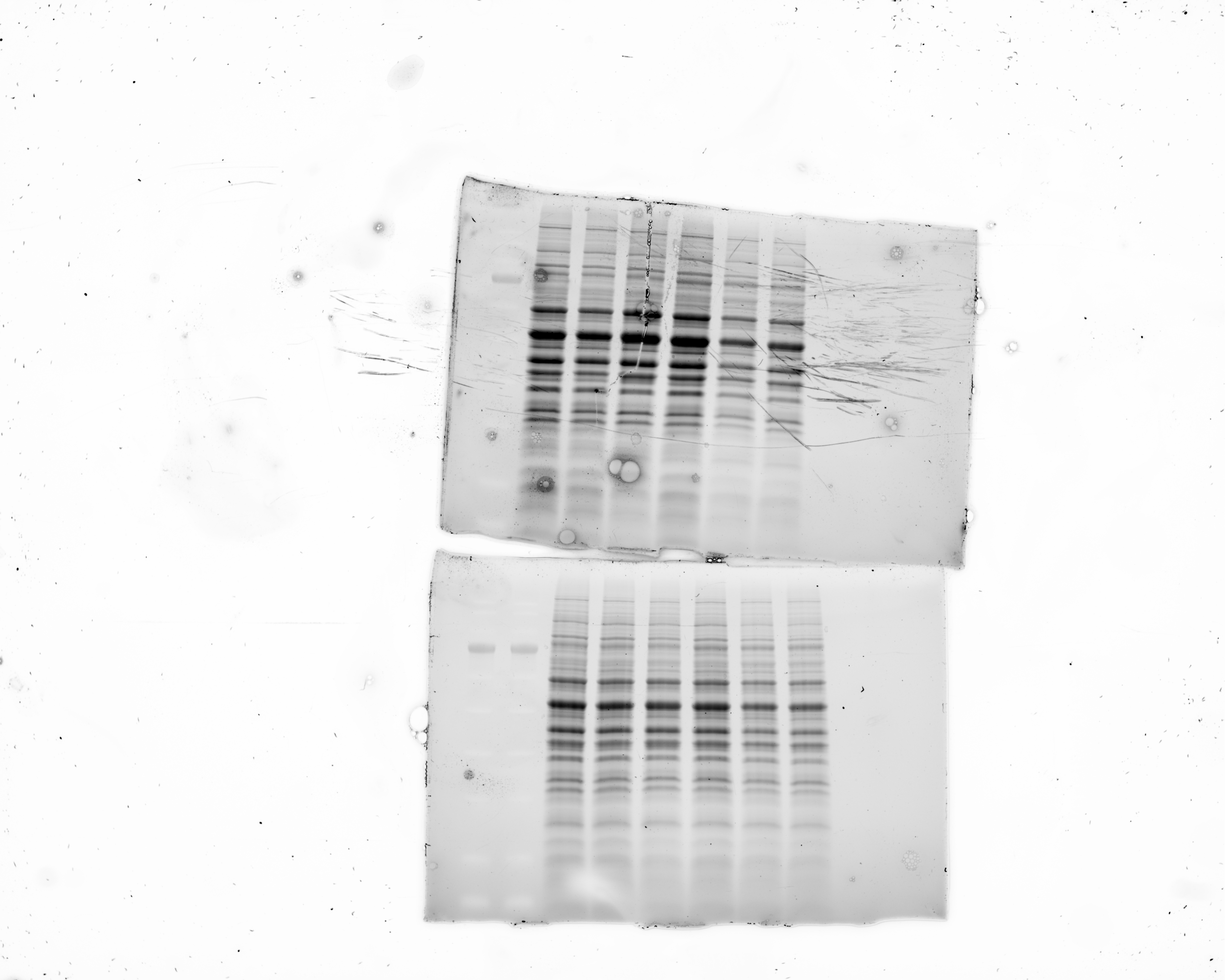

Supplement: Figure 5—source data 1. [file elife-110044-fig5-data1.zip › Figure 5-source data 1/Figure 5a-2.tif]

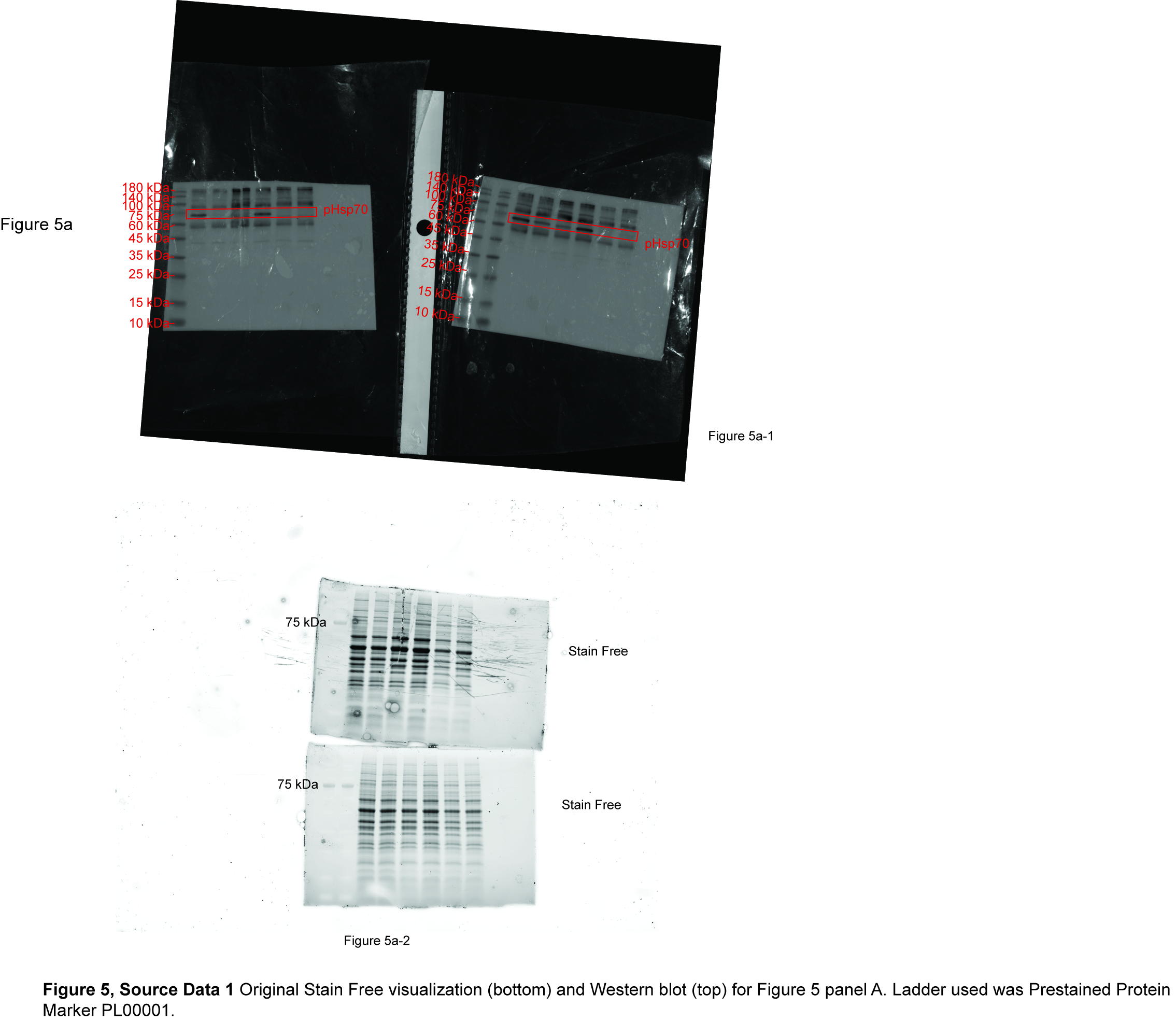

Supplement: Figure 5—source data 2. [file elife-110044-fig5-data2.zip › Figure 5-source data 2/Figure 5 source data 2.tif]
